# Allosteric regulation of the 20S proteasome by the Catalytic Core Regulators (CCRs) family

**DOI:** 10.1038/s41467-023-38404-w

**Published:** 2023-05-30

**Authors:** Fanindra Kumar Deshmukh, Gili Ben-Nissan, Maya A. Olshina, Maria G. Füzesi-Levi, Caley Polkinghorn, Galina Arkind, Yegor Leushkin, Irit Fainer, Sarel J. Fleishman, Dan Tawfik, Michal Sharon

**Affiliations:** Department of Biomolecular Sciences, Weizmann Institute of Science, Rehovot, 7610001 Israel

**Keywords:** Cryoelectron microscopy, Proteasome, Mass spectrometry

## Abstract

Controlled degradation of proteins is necessary for ensuring their abundance and sustaining a healthy and accurately functioning proteome. One of the degradation routes involves the uncapped 20S proteasome, which cleaves proteins with a partially unfolded region, including those that are damaged or contain intrinsically disordered regions. This degradation route is tightly controlled by a recently discovered family of proteins named Catalytic Core Regulators (CCRs). Here, we show that CCRs function through an allosteric mechanism, coupling the physical binding of the PSMB4 β-subunit with attenuation of the complex’s three proteolytic activities. In addition, by dissecting the structural properties that are required for CCR-like function, we could recapitulate this activity using a designed protein that is half the size of natural CCRs. These data uncover an allosteric path that does not involve the proteasome’s enzymatic subunits but rather propagates through the non-catalytic subunit PSMB4. This way of 20S proteasome-specific attenuation opens avenues for decoupling the 20S and 26S proteasome degradation pathways as well as for developing selective 20S proteasome inhibitors.

## Abstract (web summary)

A family of regulators named Catalytic Core Regulators (CCRs) oversees the function of the 20S proteasome. Here, the authors show that CCRs function through an allosteric mechanism, coupling the physical binding of the PSMB4 β-subunit with attenuation of the proteasome three proteolytic activities.

## Introduction

The cellular proteome requires continuous adaptation and modification to properly respond to changing conditions^1^. As a result, protein synthesis, folding, and degradation are tightly coordinated and regulated processes^2^. Degradation is carried out mostly by the proteasome complex, which eliminates intracellular misfolded, damaged, or unneeded proteins by two complementary degradation strategies^3–7^. In both degradation routes, proteolysis occurs within the 20S proteasome, which is made up of two outer α-rings and two inner β-rings. In eukaryotic organisms, each of the rings is composed of seven distinct α- and β-subunits^8^. To degrade folded proteins that are targeted for degradation by ubiquitin tagging, the 20S proteasome associates with one or two 19S regulatory complexes, thereby forming the 26S proteasome^9–11^. The 19S regulatory complex unfolds the ubiquitinated protein substrate and delivers it into the 20S proteolytic chamber. The second strategy, which is the focus of this manuscript, involves proteins that contain partially unfolded regions that can enter directly into the narrow 20S aperture. Although free 20S complexes are often found in a closed-gate latent state, these protein substrates activate their own degradation by gating the 20S proteasome directly in a ubiquitin, 19S and ATP-independent manner^6,7^. It should be noted that feeding these unstructured regions into the 20S proteasome is not restricted to the proteins’ termini since evidence also exists for the endoproteolytic activity of internal disordered regions^12–14^.

Two groups of substrates are mainly susceptible to direct 20S proteasome degradation. The first consists of proteins that have lost their native structure due to aging, mutations, or oxidative damage^6,7^. These proteins are prone to aggregation and may lead to cytotoxicity, and, therefore, should be rapidly removed to prevent cell malfunctions that have been associated with pathologies, such as cardiovascular disease and neurodegenerative disorders^1,2^. The second group comprises substrates with unfolded regions as an intrinsic feature^6,7^. It is estimated that more than 40% of human proteins contain intrinsically disordered regions (IDRs)^15^, making them susceptible to 20S degradation. This group includes numerous signaling and regulatory proteins, such as the tumor suppressors p53, p73 and the retinoblastoma protein, the proto-oncoprotein c-Fos, the cell cycle regulators p27 and p21, and the neurodegenerative disease-related proteins tau and α-synuclein^7,16,17^. Considering that these flexible proteins experience various conformations depending on their binding partners or cellular status, some of which can be degraded at any time and apparently, without the regulatory mechanism of ubiquitin tagging^18^, degradation through the 20S proteasome must be tightly regulated in order to prevent proteasome clogging^19^ and maintain optimal cell function and viability.

**Fig. 1:**
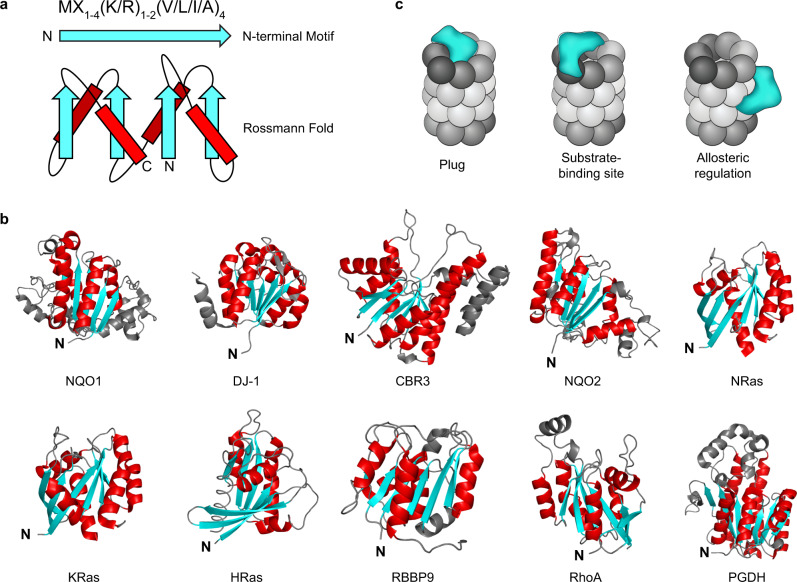
Several models may explain the CCR family mode of action. **a** A conserved N-terminal sequence motif and a Rossmann fold are common features of the CCR family. β-strands and α-helices are shown in cyan and red, respectively. **b** All experimentally verified CCRs are presented, highlighting the common Rossmann fold with β-sheets (cyan) sandwiched by α-helices (red). **c** Possible models considered in this work that may underlie the CCR (cyan) mode of action. They may (i) act like a plug, preventing substrate degradation by blocking the entrance to the 20S chamber; (ii) bind and mask a 20S proteasome substrate-binding site, thereby preventing the interaction between substrate and 20S proteasome; (iii) or induce allosteric transitions within the 20S proteasome, inducing a conformational change into a proteolytically inactive state.

Recently, we discovered a novel family of 20S proteasome regulators. This family, which we have named Catalytic Core Regulators (CCRs), consists of 17 small proteins of 20–30 kDa, many of which are enzymes (such as CBR3, NQO2, DJ-1, PGDH, and RBBP9) or key signaling proteins (as NRas, KRas, HRas, and RhoA) (Fig. 1a, b)^20^. We showed that in addition to their primary functions, these proteins have a moonlighting activity—they specifically interact with the 20S, inhibit protein degradation and thereby influence the cellular levels of 20S proteasome substrates. Bearing in mind that the CCRs were discovered based on structural and sequence similarities, namely, they all share an N-terminal sequence motif and a Rossmann fold (Fig. 1a, b), it is likely that the mechanism by which they inhibit the 20S proteasome is similar. However, their mode of function remained enigmatic.

Here, we set out to determine the molecular basis of CCRs’ ability to inhibit the 20S proteasome. Using various CCRs and 20S proteasomes from different organisms, we found that CCRs bind the proteasome through an internal β-strand that is exposed upon interaction with the 20S. Binding by the 20S proteasome is mediated by the β- rather than the α-ring, specifically by the PSMB4 subunit. Moreover, we demonstrate that the interaction formed by PSMB4 and the CCR leads to allosteric inhibition of the proteasome’s three enzymatic activities. To verify the structural underpinnings of inhibition, we showed that an artificial protein that recapitulates the basic sequence motif and structural elements of the CCRs is capable of inhibiting the 20S proteasome. The identification of this 20S proteasome-specific regulatory path offers unique opportunities for the design of new classes of inhibitors for the treatment of proteasome-related diseases.

## Results

### The CCRs CBR3 and PGDH inhibit the 20S proteasome in a noncompetitive mode

Bearing in mind that the CCRs physically bind the 20S proteasome^20^, several models could potentially explain their ability to inhibit the 20S (Fig. 1c). They can: (i) act like a physical plug, preventing substrate entry to the 20S chamber^21^; (ii) bind and mask a 20S proteasome substrate-binding site^22^; (iii) induce allosteric transitions within the 20S proteasome, stabilizing it in an inactive state^23–25^. In principle, CCRs may also act at the substrate level, i.e., capturing substrates before they are engaged for degradation. Previous studies, however, did not suggest an interaction between various CCRs and 20S substrates whose degradation they affect^20,26^. We therefore discounted this last possibility as a major path of 20S inhibition since the association between CCRs and 20S substrates is probably transient and functionally irrelevant.

**Fig. 2:**
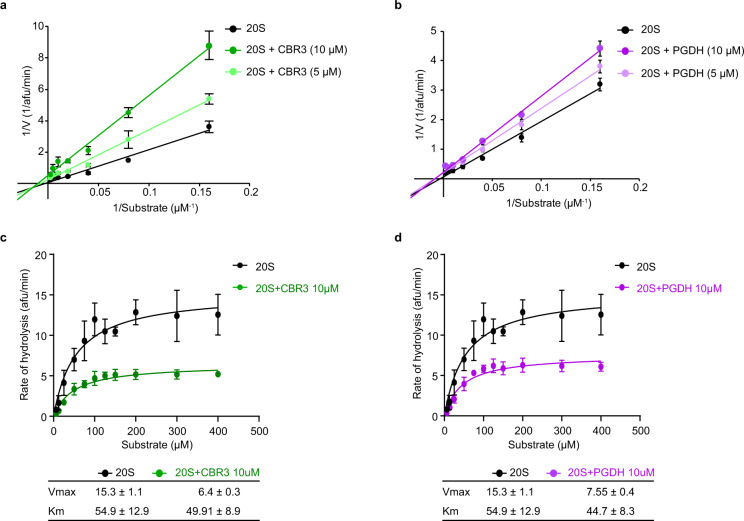
CBR3 inhibits the 20S proteasome noncompetitively. Lineweaver–Burk double reciprocal plot of the enzyme velocity (1/*V*) of the 20S proteasome versus concentration of the fluorogenic peptide substrate Suc-LLVY-AMC (1/[*S*]) in the absence and presence of different concentrations of **a** CBR3 and **b** PGDH. Data are shown as arbitrary fluorescence units per minute (afu/min). The *x*-axis intercepts for CBR3 are −0.0020 ± 0.005, −0.0066 ± 0.003, −0.0096 ± 0.003, and for PGDH −0.0027 ± 0.0035, −0.0088 ± 0.0029, −0.0082 ± 0.0020, for free 20S, and 20S with 5 and 10 μM of CCR, respectively. These values indicate that for CBR3 and PGDH, the lines intercept with the *x*-axis at the same point within error. Initial reaction velocities of 20S-dependent hydrolysis of increasing concentrations of Suc-LLVY-AMC in the presence of CCRs **c** CBR3 and **d** PGDH. Each data point represents the mean values from three independent experiments. Error bars represent standard deviation (SD). *V*_*max*_ and *K*_*m*_ values are presented in the bottom ± standard error (SEM). Source data are provided with this paper.

In order to investigate the mechanism of 20S proteasome regulation, we first aimed to determine the mode of CCR inhibition. For this, we performed kinetic assays, in which we titrated the fluorogenic substrate Suc-LLVY-AMC and measured the chymotrypsin-like activity of the 20S proteasome. The degradation rate was evaluated in the absence and presence of two CCRs, CBR3 and PGDH. We then used the Lineweaver–Burk plot, in which the reciprocal of the reaction rate (1/*V*) is plotted against the reciprocal of the substrate concentration (1/[*S*]), to determine the type of CCR inhibition. CBR3 and PGDH exhibited noncompetitive inhibition, as revealed by the change in the *y*-axis intercept for increasing the concentration of the CCRs (Fig. 2a, b). Next, we performed substrate saturation experiments for 20S proteasome in the presence of both CCRs and used the Michaelis–Menten equation and nonlinear regression fit to determine the *K*_*m*_ and *V*_*max*_. In the presence of CBR3 and PGDH, *V*_*max*_ was clearly reduced, while the *K*_*m*_ value remained the same within the error of measurement as in the presence of CCRs, a feature characteristic for a noncompetitive inhibition, although mixed inhibition cannot be ruled out (Fig. 2c, d). These results suggest that the CCRs do not compete with substrate binding or block the entrance into the 20S proteasome chamber, contradicting models (i) and (ii) (Fig. 1c). Rather, CCRs shift the 20S complex into a proteolytically repressed conformational state, thus possibly regulating the 20S proteasome allosterically. Overall, the results support our previous analysis using native MS that demonstrated that various CCRs physically bind the 20S proteasome irrespective of the presence or absence of the 20S substrate^20^.

### A balance between structural rigidity and flexibility is required for the CCR function

The CCR family is characterized by both sequence and structural features, namely an N-terminal sequence motif [MX_1-4_(K/R)_1-2_(V/L/I/A)_4_] and a Rossmann fold (Fig. 1a). We therefore asked what the functional relevance of these two elements is? Focusing initially on the CCR DJ-1, we generated a mutant that lacks the seven residues of the conserved N-terminal motif, DJ-1_ΔN_ (Supplementary Fig. 1a). Native MS analysis revealed a monomeric rather than dimeric composition of DJ-1_ΔN_. Moreover, examination of the spectrum indicated that in addition to the folded monomeric protein, it includes a charge state envelope with a broad distribution of high charge states in comparison to wild-type (WT) DJ-1, implying a partially unfolded conformation (Supplementary Fig. 1a). Further, DJ-1_ΔN_ displayed a lower melting temperature (Tm) in comparison to the WT protein, emphasizing its reduced stability (Supplementary Fig. 1b). These results are in agreement with this N-terminal motif forming the Rossmann’s first β-strand, which is buried at the domain’s core and may lead to reduced stability upon removal. Degradation assays revealed that, unlike the WT protein, DJ-1_ΔN_ serves as a substrate for the 20S proteasome, as do other intrinsically unstructured/disordered proteins (Supplementary Fig. 1c). Thus, deletion of the N-terminal region, which forms the core β-strand^27^, will impede its proper folding as expected from its interior position.

Next, we examined if the N-terminal motif is sufficient for the CCR function. Initially, we fused the 15-residue N-terminal motif of CBR3 to the cerulean fluorescent protein (Cer) and performed degradation assays (Supplementary Fig. 1d). In contrast to CBR3, the CBR3_N-term_-Cer fusion could not efficiently inhibit the 20S proteasome function. We continued by examining the major carbonyl reductase, CBR1, which shares 72% sequence similarity with CBR3^28^ (Supplementary Fig. 1e) but does not contain the N-terminal motif (CBR1 is missing the positively charged residue in the motif). We found that, unlike CBR3, CBR1 does not inhibit the 20S proteasome (Supplementary Fig. 1f). Exchanging the N-termini of CBR3 and CBR1 did not show a robust impact on CBR1 or vice versa (Supplementary Fig. 1f). Taken together, these results suggest that the N-terminal motif per se is necessary but insufficient for CCR activity.

To identify the additional regions within the Rossmann fold that are involved in 20S proteasome binding, we applied peptide-array screening^29,30^. The peptide-array library included peptides from five different CCR proteins: DJ-1 from human, yeast (*Saccharomyces cerevisiae*, Hsp32) and archaea (*Thermoplasma acidophilum*, Ta0465, TA DJ-1), and human CBR3 and NQO1 (Supplementary Fig. 2 and Supplementary Data 1). Binding experiments were conducted with three 20S proteasome orthologs isolated from archaea (*T. acidophilum*), yeast (*S. cerevisiae),* and human (HEK293T) cells. These 20S proteasome orthologs were already shown to be inhibited by human CCRs^20^, and we therefore used them in order to find conserved CCR binding sites. To rule out the non-specific binding of the probing antibodies, a control experiment was carried out without the addition of the 20S proteasome (Supplementary Fig. 2b, d). The anti-His antibody did not show any unspecific binding to the peptide spots (Supplementary Fig. 2b), whereas the anti-FLAG antibody did bind to a few spots (Supplementary Fig. 2d), and these were disregarded in further analysis when 20S proteasomes from yeast and human were used.

As a positive control, C-terminal peptides from the 19S ATPase subunits, PSMC1 and PSMC3, that are known to bind the eukaryotic 20S proteasome were used, whereas a peptide derived from the PAN ATPase was used for the archaeal 20S proteasome^31^ (Supplementary Fig. 2f).

**Fig. 3:**
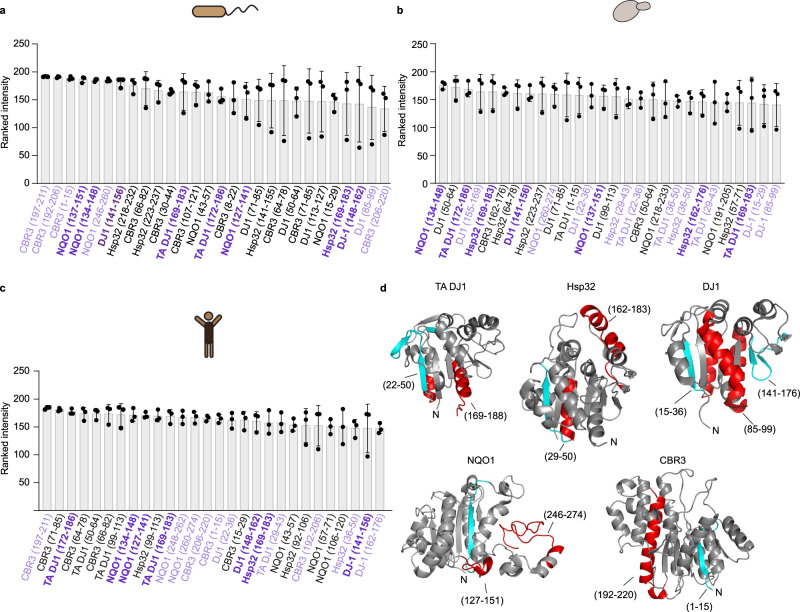
Evidence that CCRs undergo conformational changes upon binding to the 20S proteasome. **a**–**c** Peptide-array screening for 20S proteasome binding to CCR-derived peptides. The bar graphs present the relative binding of the various CCR peptides to 20S proteasome derived from **a** archaea, **b** yeast, and **c** human. Peptides highlighted in lilac and purple are bound by two and three proteasome orthologs, respectively. Averaged values from three independent experiments are presented, and error bars represent SD. **d** Consensual binding CCR peptides to at least two proteasomes are mapped and highlighted in red for the exposed α-helices or loops and in cyan for the buried β-strand on the various CCR structures (see table in Supplementary Fig. 2g). Source data are provided with this paper.

Each type of proteasome reacted with 25–35 peptides, with the yeast proteasome being the most prominent binder (Supplementary Figure 2a, c, e). For each of the proteasome orthologs, we focused only on the top 15% of all the reactive peptides (see the Methods section and Fig. 3a–c). Of these, we considered only those peptides that interacted with at least two of the three 20S proteasome orthologs (Supplementary Fig. 2g). This analysis led to the identification of a binding region within each of the CCRs (Fig. 3d). Although we could not identify a common sequence motif among these binding sites, we noticed that for all the CCRs, the binding site is mapped to an unexposed interior β-strand of the Rossmann fold along with exposed α-helices and loops (Fig. 3d). Thus, these results suggest that CCRs probably bind to the 20S proteasome with the exposed α-helices or loops, followed by conformational transitions that expose a buried β-strand. Such conformational transition would demand a protein core that can be rearranged, namely a structured yet marginally stable protein.

**Fig. 4:**
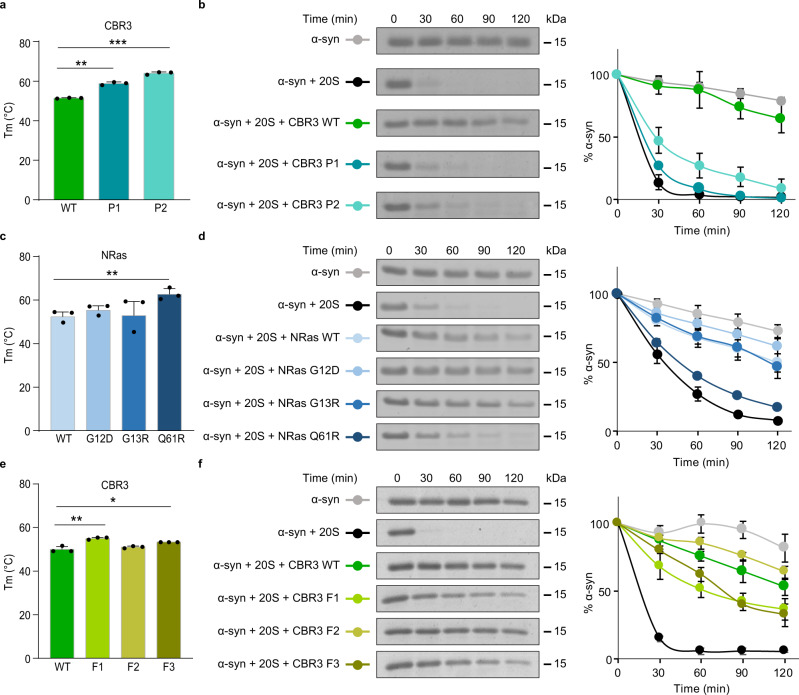
CCRs function requires a balance between structural rigidity and flexibility. **a**, **c,** **e** Melting temperatures (Tm) measured for CBR3 PROSS (P) and FuncLib (F) designs and NRas cancer-associated mutants. Each bar represents the mean values of three independent experiments. Measurements were subjected to one-tailed Student’s *t*-test analysis, *p < 0.05, **p < 0.01, ***p < 0.001. (**a** ** represents *p-*value = 0.0017, *** represents *p-*value = 0.0004). (**c** ** represents *p-*value = 0.0043). (**e** ** represents *p-*value = 0.0078, * represents *p-*value = 0.0229). Error bars represent SD. **b**, **d**, **f** Time-dependent degradation assays using α-synuclein (α-syn) as the 20S proteasome substrate in the presence of CBR3 **b** PROSS and **f** FuncLib designs, and **d** cancer-associated NRas mutants. Averaged quantification of three independent experiments is displayed on the right; error bars represent SD. 20S proteasomes were purified from rat livers. Given the variability that occurs due to age, sex, genetics, and health conditions of the animals, batch effects are observed. Therefore, the same batch of purified 20S proteasomes was used for all repeats of a particular experiment (for each panel). In spite of the batch effect that influences degradation kinetics, the difference in the CCR activity of the WT proteins and mutational variants is clearly detected. Source data are provided with this paper.

To validate the above assumption, we designed two CBR3 variants using the PROSS (P) stability-design algorithm^32^ (Supplementary Fig. 3a, b). The rationale was to generate CBR3 variants that on the one hand maintain the Rossmann fold but on the other hand display improved core packing relative to the WT protein, thus hindering the structural transformation that is required for 20S proteasome binding and inhibition. We first verified that the two CBR3-P designs displayed increased thermal stability in comparison to WT CBR3 (Fig. 4a) as predicted by the design method. Degradation assays indicated that as expected, the CBR3 P1 and P2 designs significantly lost the ability to regulate the 20S proteasome (Fig. 4b). Nevertheless, the enzymatic activity of the CBR3 variants was increased relative to WT CBR3 (Supplementary Fig. 4a).

Since catalytic activity depends on fine structural details, the conservation (and in fact, improvement) of CBR3’s primary catalytic activity verified that its structure was conserved despite the introduction of 14 and 20 mutations in P1 and P2 designs, respectively. Furthermore, the results demonstrated that the primary catalytic activity of a CCR and its 20S proteasome inhibition activity could be decoupled through the design of stabilizing mutations. Similar results were obtained when we analyzed the known cancer-associated mutations of the CCR NRas, G12D, G13R, or Q61R^33,34^ (Supplementary Fig. 3c). Like the CBR3 P1 and P2 designs, the melting point of the NRas-Q61R mutant is higher than that of G12D, G13R, and WT NRas proteins (Fig. 4c), an observation that is correlated with the reduced ability of Q61R to regulate the 20S proteasome (Fig. 4d). Taken together with the DJ-1_ΔN_ results (Supplementary Fig. 1a–c), the data suggest that CCR functionality depends on a balance between structural stability and flexibility: on the one hand, protein flexibility is required for conformational transition upon 20S proteasome binding, and on the other hand, structural stability is needed to protect the CCRs themselves from 20S proteolysis.

Next, to confirm the relevance of the identified CBR3 β-strand (^5^SRVALVTGANR^15^) (Fig. 3 and Supplementary Fig. 2g), which includes the N-terminal motif, as the 20S proteasome binding site, we used the FuncLib (F) algorithm^35^ to design mutants in this region without disrupting the scaffold of the protein. Whereas the PROSS stability-design algorithm preferentially introduces mutations to the protein surface, FuncLib design calculations can be focused on a small region of the protein core and compute dense constellations of amino acid residues that improve native-state energy^36–38^. Three CBR3-FuncLib mutants (F1-F3) were designed and expressed (Supplementary Fig. 3d, e). WT and F2 CBR3 displayed similar melting points, whereas F1 and F3 exhibited higher thermal stability (Fig. 4e). Degradation assays revealed the reduced ability of F1 and F3 to inhibit proteasome activity (Fig. 4f), as observed for P1 and P2. F2 CBR3, however, demonstrated increased capacity of 20S proteasome regulation. Nevertheless, all FuncLib mutants were catalytically inactive (Supplementary Fig. 4b), probably due to their inability to bind the NADPH cofactor (Supplementary Fig. 3e), again emphasizing that the primary enzymatic activity of CCRs and their 20S inhibition activities can be decoupled. Sequence analysis of the CBR3-F designs revealed that F1 and F3, unlike F2, contain more hydrophobic residues in comparison to the WT protein (Supplementary Fig. 3d), suggesting that hydrophobicity may have a role in CBR3’s capacity to regulate the 20S proteasome. Support for this view comes from our previous study in which we investigated the Parkinson’s disease-associated mutational variant of DJ-1, D149A, which is located in the identified β-strand binding region (Supplementary Fig. 2g). This mutant exhibits decreased thermal stability, which is correlated with increased capacity to inhibit the 20S proteasome^39^. Taken together, these observations (Figs. 3, 4 and Supplementary Figs. 1–4), lend support to our working model that modulation of the β-strand 20S binding segment affects the ability of the CCR to regulate the 20S proteasome activity, regardless of the CCR’s primary catalytic activity.

### CCR activity is independent of the 20S proteasome gate conformation

**Fig. 5:**
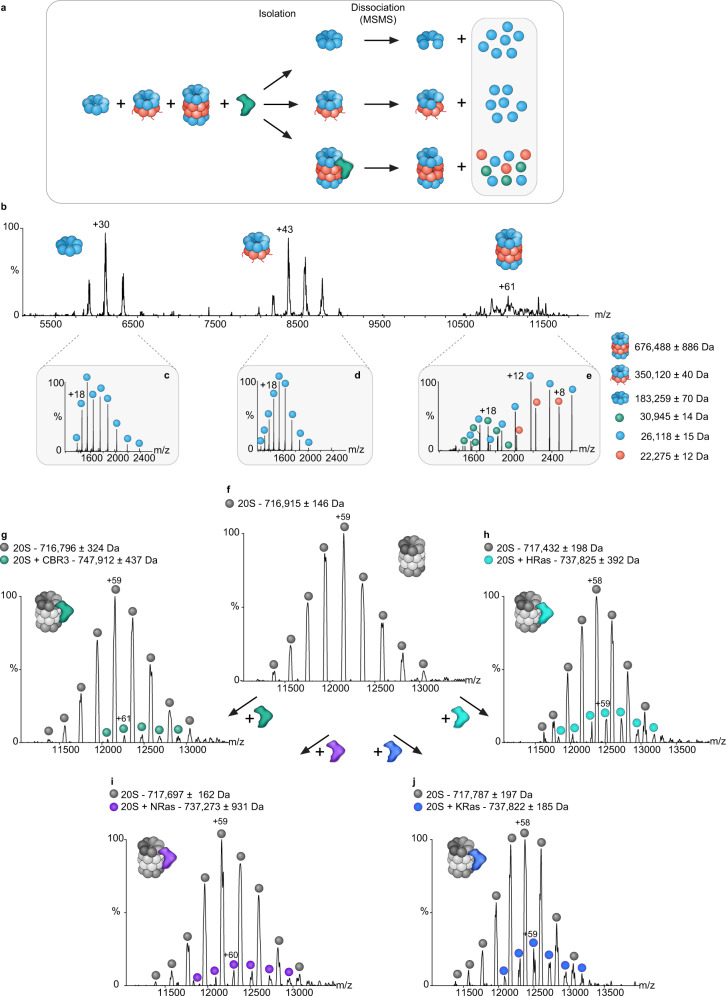
CCRs bind to the 20S proteasome. **a** Schematic representation of the native MS methodology (applied in (**b**–**e**)), in which the CCR CBR3 is incubated with a mixture containing the *T. acidophilum* α-ring (α_7_), an unprocessed half proteasome (α_7_β_7_) and the fully assembled proteasome (α_7_β_7_β_7_α_7_). The different complexes are isolated and subjected to increased collision energy. This leads to the dissociation and detection of 20S proteasome subunits and bound CBR3 (indicated by the gray box), leaving a stripped complex. **b** Native MS spectrum of the archaeal 20S proteasome mixed with CBR3. The α-ring (α_7_), half proteasome (α_7_β_7_), and fully assembled 20S proteasome (α_7_β_7_β_7_α_7_) are detected. For each of these complexes, the most intense charge state obtained in the MS spectrum was subjected to MS/MS analysis. Comparison of the data revealed additional peaks that correspond in mass to CBR3 only in the MS/MS spectrum of the fully assembled proteasome (**e**) but not in the half proteasome (**d**) or α-ring assemblies (**c**). Blue and red circles correspond to 20S proteasome α- and β-subunits, respectively. CBR3 is labeled with green circles. **f** Native MS spectrum of the rat 20S proteasome. **g**–**j** 20S proteasomes were pre-incubated with different CCRs (CBR3, NRas, KRas, and HRas) and subjected to native MS analysis to identify the stoichiometry of CCR binding. In addition to the free 20S proteasome, unique charge series corresponding in size to the 20S proteasome bound to a single CCR were detected (colored balls), indicating a 1:1 stoichiometry.

We next focused on identifying the CCR binding region within the 20S proteasome. To this end, we used the *T. acidophilum* complex, which is a simpler form of the 20S proteasome that was shown to bind human CCRs^20^. Unlike the eukaryotic complex, the archaeal proteasome contains only one type of α- and β- subunits^40^, and it assembles spontaneously^41^, making the analysis more feasible. Initially, we incubated the purified His tagged α- and β-subunits individually with CBR3, loaded them on a Ni-NTA column, and monitored the elution profile. CBR3 failed to bind the column, suggesting that it does not interact with the α_7_ ring, which forms spontaneously, and non-assembled β-subunits (Supplementary Fig. 5a, b). We then expressed both the α- and β-subunits of the *T. acidophilum* complex and induced complex assembly under non-optimal conditions that led to the co-existence of the α-ring (α_7_), an unprocessed half proteasome (α_7_β_7_) containing the β−subunit propeptides, and the fully assembled proteasome (α_7_β_7_β_7_α_7_)^42^ (Fig. 5a, b and Supplementary Fig. 6a). CBR3 was then added to the mixture and tandem MS/MS analysis was carried out (Fig. 5c–e). These experiments involve the isolation of specific charge series corresponding to each of the proteasome assemblies. The isolated complexes are subjected to high collision energies, leading to the dissociation of any bound protein as well as individual proteasome subunits. Peaks corresponding in mass to CBR3 were detected only in the MS/MS spectrum containing the full proteasome (Fig. 5e) but not in the tandem MS spectrum of the half-proteasome species and α-ring (Fig. 5c, d). These results suggest that CBR3 preferably binds to the fully assembled 20S proteasome complex.

To determine the stoichiometry of the CCR/20S proteasome interaction, we mixed four different CCRs with the rat 20S proteasome: CBR3, HRas, NRas, and KRas. Native MS measurements indicated the co-existence of two populations, the free 20S proteasome as well as the 20S proteasome bound to one CCR (Fig. 5f–j and Supplementary Fig. 6b–e). These results are in accord with our previous observation that one NQO1 dimer binds the 20S proteasome complex^43^. However, given the symmetrical architecture of the 20S proteasome, we cannot exclude the possibility that two CCRs bind the complex; however, assemblies containing two CCR proteins were not detected, either due to their absence or their low levels, which are below the sensitivity of the instrument.

**Fig. 6:**
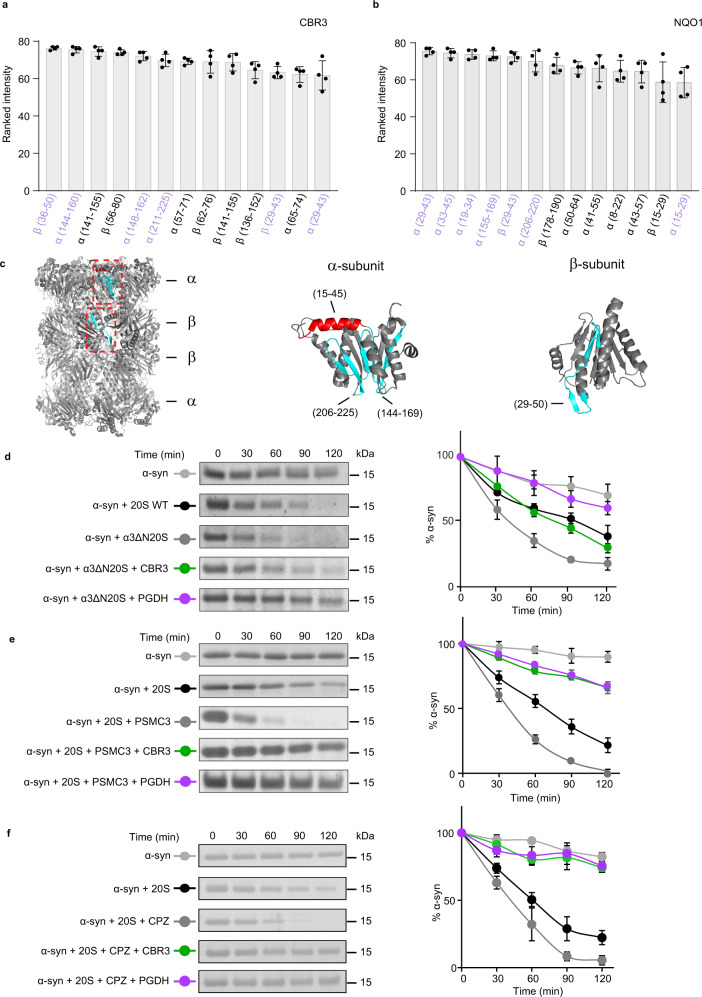
CCRs do not bind to the 20S proteasome orifice. **a**, **b** Peptide-array screening for CCR binding region to archaeal 20S proteasome derived peptides. The bar graphs present the average of the ranked intensities from four independent experiments for the relative binding of **a** CBR3 and **b** NQO1 to the various 20S proteasome peptides. Regions that are bound to both CBR3 and NQO1 are highlighted in lilac. Error bars represent SD. **c** 20S proteasome peptide sequences bound to both CBR3 and NQO1 are highlighted in red for α-helices and in cyan for β-strands, on α- and β-subunits of the 20S proteasome structure. **d** Representative in vitro degradation assays using the yeast open-gate mutant (α3_Δ_N) and the wild-type (WT) 20S proteasome. Assays were performed with the model substrate α−synuclein (α-syn) and the mammalian CCRs CBR3 and PGDH. **e**, **f** In vitro degradation assays using the rat 20S proteasome and α-syn, in the presence of CBR3 and PGDH with the gate opening peptide from the C-terminus of PSMC3 (**e**) and chlorpromazine (**f**). Averaged quantification of three independent experiments is displayed on the right. Error bars represent SD. Source data are provided with this paper.

To map the binding site region of the CCRs on the 20S proteasome, we again used peptide-array screening. This time the array was designed based on peptide sequences of the single *T. acidophilum* α- and β-subunits (Supplementary Data 2) and probed with the two human CCRs, NQO1 and CBR3 (Supplementary Fig. 5c, d). As a control, the anti-NQO1 and CBR3 antibodies were incubated with the array; however, neither of them showed any relevant binding (Supplementary Fig. 5c, d, right panels). Considering peptide spots with intensities within the top 17%, four common sequences from eleven overlapping 20S proteasome peptides bound to both CBR3 and NQO1 (Fig. 6a, b and Supplementary Fig. 5e), were mapped to a β-loop-β secondary structure within the α- and β-subunits (Fig. 6c in cyan) and an α-helix within the α-subunit (Fig. 6c in red).

To further characterize the CCR binding site and probe whether it depends on the status of the substrate entry gate, we took advantage of established methods to induce gate opening in 20S proteasomes. Specifically, we used a mutational variant of the yeast (*S. cerevisiae*) 20S proteasome in which nine residues at the N-terminus of the PSMA4 (α_3_) subunit were deleted, generating a constitutively open gate (α3ΔN)^44^, or added the 20S proteasome gate-opening peptide from the C-terminus of PSMC3, a 19S subunit (^421^KANLQYYA^428^)^31^. In addition, chlorpromazine (CPZ), a small-molecule enhancer of the 20S proteasome activity^45^, was used to induce gate opening. Despite the induction of gate-opening, as indicated by the enhanced degradation capacity of the proteasome, the CCRs CBR3 and PGDH protected α-synuclein from degradation. PGDH inhibition capacity was not dependent on the gate status (Fig. 6d–f). Reduced activity was detected for CBR3 for the yeast open-gate mutant; however, gate opening induced by the PSMC3 peptide and CPZ did not affect the inhibition capacity.

These results suggest that CCRs regulate the 20S proteasome independent of the conformation of the substrate entry gate. Taken together with the native MS analysis and peptide-array screening, the results suggest that CCRs do not bind to the orifice of the proteasome but rather to an exposed region on the barrel surface.

### CBR3 binds to the PSMB4 subunit of the eukaryotic 20S proteasome

**Fig. 7:**
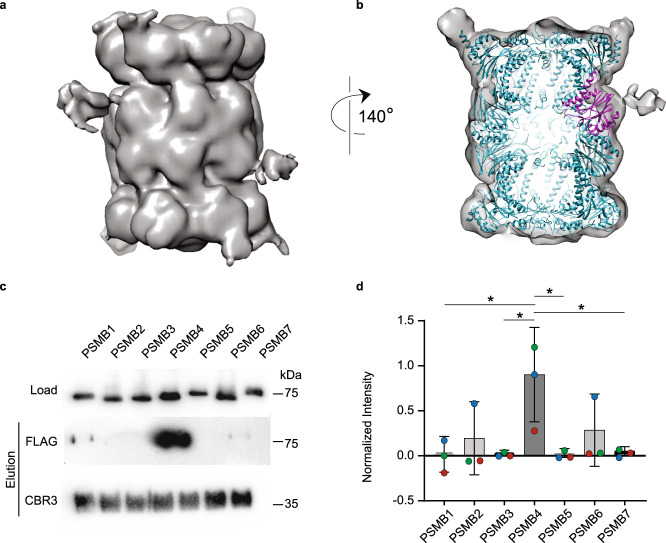
CBR3 binds the 20S proteasome β-ring and specifically to the PSMB4 subunit. **a** Cryo-EM structure of the rat 20S proteasome/CBR3 complex. A prominent extra density is displayed at the β-ring region. **b** Atomic model of the 20S proteasome structure (PDB:6TU3) (cyan) was fitted into the electron density map. The extra electron density near the PSMB4 subunit (magenta) reveals the binding site of CBR3. **c** *Escherichia coli* cell lysates overexpressing FLAG-tagged human β-subunits PSMB1 to PSMB7 were incubated with His_6_-CBR3 bound to Ni-beads for pull-down experiments. Bound subunits were detected using an anti-FLAG antibody. **d** Densitometry quantification indicated that the pull-down of PSMB4 greatly exceeds the levels of the other β-subunits. The bar graph shows the average values of each PSMB subunit intensities in the pull-down, normalized to the corresponding lysate, from three independent replicates; each replica is color-coded (red, green, and blue). Error bars represent SEM. Significance was calculated using one-way ANOVA (*p-*value = 0.0309), with Dunnett’s post-hoc test (* represents *p-*value = 0.0145, 0.0168, 0.0163, 0.0191 for the significances between PSMB4 and PSMB1, PSMB3, PSMB5, and PSMB7, respectively). Source data are provided with this paper.

To gain further insight into the 20S proteasome/CCR interaction, we applied single particle cryo-electron microscopy (EM) analysis using the rat 20S proteasome and human CBR3. A dataset of ∼55,000 proteasome particles was collected, wherein more than 50% of the particles showed extra density, comparable to the size of CBR3 near the β-ring (Fig. 7a). All particles used for 2D classification (Supplementary Fig. 7, Supplementary Table 2), were also used for 3D classification generating a moderately resolved cryo-EM map at 12 Å, implying a high degree of conformational heterogeneity in the system. The cryo-EM data indicated additional density on both PSMB4 subunits, one on each ring. However, because particle-averaging methods were employed to generate these images, it is not possible to determine unambiguously whether the two CBR3 proteins are occupied simultaneously.

In spite of the moderate resolution of the CBR3/20S proteasome structure, it provided sufficient quality for docking the atomic map of the free rat 20S proteasome^46^ as a rigid body (Fig. 7b, Supplementary Data 3). This enabled us to confidently determine that CBR3 specifically binds to the PSMB4 subunit of the proteasome, which is a non-catalytic β-ring subunit. While the binding region within CBR3 is not clear, we could determine that the binding region within PSMB4 includes residues 15–20, 115–120, and 192–196. This region is characterized mainly by β-loop-β secondary structures, supporting our peptide array results (Fig. 6c). In addition, the implied conformational heterogeneity of CBR3 suggests that upon binding to PSMB4, the protein undergoes structural rearrangements and therefore may interact with the adjacent β-loop-β secondary structure identified in the archaeal α-subunit (Fig. 6c).

To confirm the association between CBR3 and PSMB4, we independently overexpressed in *E. coli* all human β-subunits (PSMB1 to PSMB7) of the 20S proteasome fused to a solubility tag and a C-terminal FLAG tag and performed pull-down experiments with His tagged CBR3 (Fig. 7c). Among all the β-subunits, the PSMB4 subunit was repeatedly pulled down with CBR3 at the highest levels, suggesting that this subunit indeed harbors an interacting site for CBR3 within the 20S proteasome (Fig. 7d). We also noticed that the exposed β-loop-β secondary structures of PSMB4 and the first β-strand of CBR3, which was identified as the 20S proteasome binding region (Fig. 3d), display electrostatic complementarity (Supplementary Fig. 8), supporting an interaction at these regions.

**Fig. 8:**
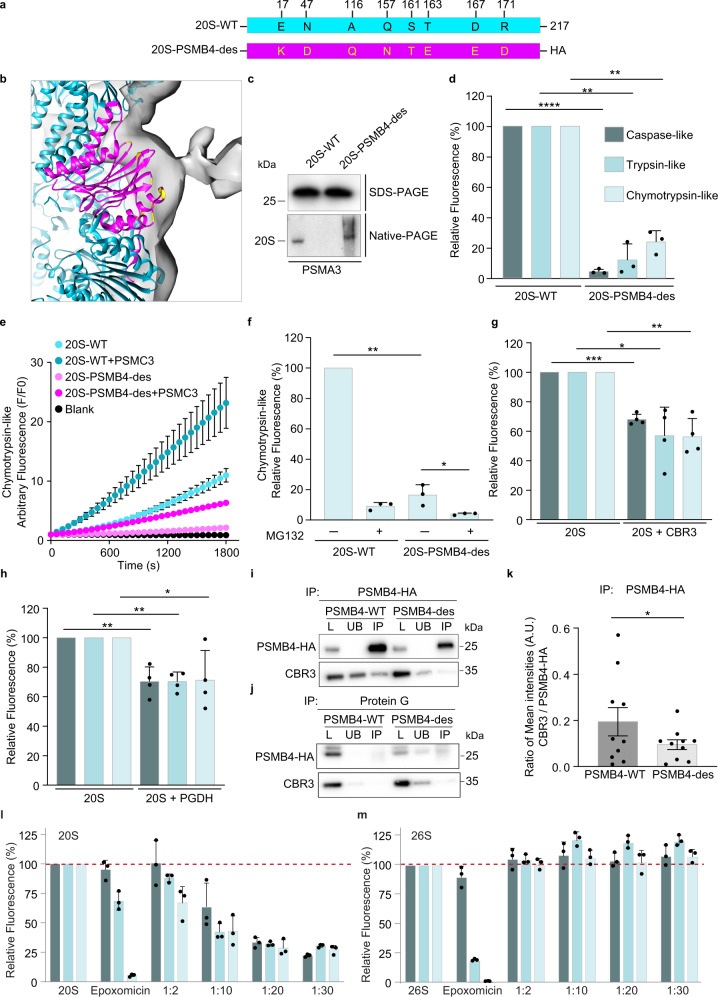
CBR3 binds the PSMB4 subunit of the 20S proteasome. **a** Schematic representation of human PSMB4-WT and 20S-PSMB4-des engineered to attenuate CBR3 binding. The mutated residues are highlighted in yellow. **b** Zoom-in view of the PSMB4 subunit (magenta) with mutated residues highlighted in yellow. **c** Purified proteasomes were analyzed by SDS- and native-PAGE and blotted with an anti-PSMA3 antibody, indicating the reconstitution of holo-20S proteasomes consisting of the HA-tagged PSMB4-WT or des subunit. **d** The chymotrypsin-, trypsin- and caspase-like activities of the purified WT 20S proteasome and those incorporating the PSMB4-des subunit were measured using fluorogenic peptide substrates. A pronounced reduction in all three enzymatic activities was detected for the 20S-PSMB4-des complex. Averaged quantification of three independent experiments; error bars represent SD. **e** The chymotrypsin-like activity of WT 20S proteasome and the 20S-PSMB4-des was measured in real-time in the presence and absence of the gate-opening PSMC3 C-terminus peptide. Averaged quantification of three independent experiments; error bars represent S.D. **f** The influence of the proteasome inhibitor, MG132, on WT 20S proteasome and on 20S-PSMB4-des was measured by using the fluorogenic peptide substrate suc-LLVY-AMC. The graph represents averaged values from three independent repeats; error bars represent SD. The impact of the CCRs **g** CBR3 and **h** PGDH on the chymotrypsin-, trypsin-, and caspase-like activities of the purified rat 20S proteasome was monitored using fluorogenic peptide substrates. Similar to the 20S-PSMB4-des complex, CBR3 and PGDH significantly reduced all three enzymatic activities of the proteasome. Bars and scatter plots represent mean values from three or four independent experiments (in **f** and **g**, **h**, respectively), and error bars represent SD. Measurements were subjected to two-tailed (**d**) and one-tailed (**f**–**h**) Student’s *t*-test analysis (**d** **** represents *p-*value = 0.00007, ** for trypsin-like represents *p-*value = 0.0047, ** for chymotrypsin-like represents *p-*value = 0.0031). (**f** ** represents *p*-value = 0.0011, * represents *p-*value = 0.0433). (**g** *** represents *p-*value = 0.0002, * represents *p-*value = 0.0108, ** represents *p-*value = 0.0027). (**h** ** for caspase-like represents *p-*value = 0.0048, ** for trypsin-like represents *p-*value = 0.0015, * represents *p-*value = 0.0326). **i**, **j** PSMB4-WT-HA or PSMB-des-HA and CBR3 were overexpressed in HEK293T cell. Lysates were subjected to IP using either anti-HA agarose (**i**) or uncoupled protein G beads as a control (**j**). Total starting lysate (L), unbound proteins (UB), and IP samples were analyzed by Western blot using anti-HA or anti-CBR3 antibodies. **k** Bands corresponding to the pull-down of CBR3, WT-, and des- PSMB4-HA were quantified, and the ratio of CBR3 to PSMB4-HA (WT and des) are presented as bar graph. Mean values from 10 independent experiments were subjected to paired one-tailed Student *t*-test analysis, * represents *p-*value = 0.05. Error bars represent SEM. The catalytic activities of the human **l** 20S and **m** 26S proteasomes were measured in the presence of increasing concentrations of the CCR PGDH. A concentration-dependent increase in inhibition of the 20S proteasome is detected, while the 26S proteasome is not inhibited at any concentration of PGDH tested. Bars represent mean values from three independent experiments. Error bars represent SD. Source data are provided with this paper.

To further validate the PSMB4 binding site, we initially applied the PROSS stability-design algorithm to design a PSMB4 variant in which the CBR3 interaction region is modified but without disrupting the other structural regions and overall subunit fold (Fig. 8a, b). The latter aspect is especially important as PSMB4 contributes to the assembly of the 20S proteasome complex^47^. The 20S-PSMB4 design (20S-PSMB4-des), which included eight mutations, was fused to an HA tag (Fig. 8a, b), transiently overexpressed in HEK293T cells, and purified using anti-HA resin. Native-PAGE and MS analyses confirmed the assembly of PSMB4-des into the 20S proteasome (Fig. 8c, Supplementary Figs. 9 and 10). However, unlike the WT 20S proteasome, at equimolar concentrations, the complex containing the PSMB4-des displayed a significant decrease in all three enzymatic activities, caspase, chymotrypsin, and trypsin-like activities (Fig. 8d). The addition of the gate-opening PSMC3 C-terminal peptide^31^ enhanced the chymotrypsin-like activity of the PSMB4-des (Fig. 8e), validating that the proteasome gate is unaltered and functional. The addition of MG132 further reduced the chymotrypsin-like activity, indicating that the decrease in activity induced by the PSMB4-des is limited yet significant relative to the WT proteasome complex (Fig. 8f). Taken together, it is likely that the mutations stabilize the same conformation of PSMB4 as when bound to CBR3, consequently leading to a CCR-like inhibition effect on the 20S proteasome.

We then examined the impact of CBR3 and PGDH on the three different enzymatic activities of the 20S proteasome. Like the 20S-PSMB4-des, both CCRs significantly reduced all three enzymatic activities of the proteasome (Fig. 8g, h). PSMB4 resides beside the caspase-like proteolytic subunit, PSMB6 (Supplementary Fig. 11), likely explaining the reduction in its catalytic activity upon CCR binding. PSMB5 and PSMB7, which have chymotrypsin- and trypsin-like activities, respectively, are both separated from PSMB4 by a single β-subunit within the same ring. However, PSMB4 is proximal to the PSMB7 in the trans-ring (Supplementary Fig. 11). The long distance of PSMB4 from PSMB5 in both cis and trans rings suggests that allosteric transitions within PSMB4 are the cause for the chymotrypsin activity inhibition. Moreover, the fact that the three enzymatic activities are not influenced in the same manner by CCR binding supports our working model that they do not function as a plug. Overall, the data suggest that allosteric transitions influence the entire β-ring conformation, which in turn affects three proteolytic activities.

To provide additional evidence for the binding of CBR3 to PSMB4, we performed immunoprecipitation (IP) experiments (Fig. 8i–k). PSMB4-WT-HA or PSMB4-des-HA and CBR3 were transiently co-expressed in HEK293T cells. Whole cell lysates were IP’d with anti-HA resin. A control IP using uncoupled Protein G beads was performed in parallel to ascertain the background levels of the proteins binding to the beads during the IP. The level of bound CBR3 was then analyzed by Western blotting (Fig. 8i, j). Quantification of the levels of the CBR3 being pulled down with PSMB4-WT-HA in comparison to PSMB4-des-HA confirmed that the structural transition implied by PSMB4-des-HA perturbs its interaction with the CBR3 (Fig. 8k).

Previously, we showed that the CCRs bind specifically to the 20S proteasome but not to the 26S complex^20,26^. However, given that the binding sites in PSMB4 are exposed both in the 20S and 26S proteasomes, we wished to validate that the CCR functional impact is exclusively directed toward the 20S proteasome rather than to both proteasome complexes. We therefore performed a concentration-dependent experiment measuring CCR impact on the three catalytic activities of the human 20S and 26S proteasomes. To generalize the results, we choose to use PGDH rather than CBR3, which was utilized in the cryo-EM analysis. Compared with the proteasome inhibitor expoxomicin, which drastically reduced the chymotrypsin activity and partly the trypsin activity of both 20S and 26S proteasome (Fig. 8l, m), a dose-dependent reduction of all three proteolytic activities was only detected for the 20S proteasome in the presence of PGDH. Regardless of the increased levels of PGDH, no reduction in any of the catalytic activities was observed for the 26S proteasome. This result further strengthens the view that the CCRs are specific regulators of the 20S proteasome, probably binding a PSMB4 conformation that is accessible only in the free 20S proteasome form and not the 26S complex.

### A de novo-designed protein mimicking the CCR features inhibits the 20S proteasome

Having established that (1) the N-terminal motif is necessary yet insufficient for CCR function (Supplementary Fig. 1) and that (2) a balance between structural rigidity and flexibility of the Rossmann fold is required for CCR function (Fig. 4), we directed our efforts toward obtaining an artificial minimal protein construct that possesses CCR activity.

To dissect the minimal structural elements that are required for CCR function, we turned our attention to a recently designed family of simple (only ~100 residues) and functional polypeptides harboring tandem repeats of the P-loop Walker-A motif^48^. This motif, a β-strand connected to an α-helix via a phosphate-binding loop (P-loop), is a key element of both the P-loop NTPase and the Rossmann folds. Further, both folds are in fact tandem repeats of the β/α/β element arranged in a three-layered α/β/α sandwich architecture^49^. Considering that the Rossmann fold is common to all CCRs^20^ and that many of the de novo-designed P-loop proteins possess a Rossmann-like α/β/α sandwich architecture and contain the CCR N-terminal motif, we were motivated to examine whether these simple proteins can act as CCRs.

Seven designed P-loop proteins were expressed, purified, and subjected to degradation assays (Supplementary Table 1 and Supplementary Fig. 12). While most of the constructs were partially unfolded and, as a result, degraded by the 20S proteasome, three polypeptides named C-PLoop, E-PLoop, and 2N3Z, were protected from degradation, indicating their folded character (Supplementary Fig. 12a). Notably, 2N3Z that lacks the P-loop but contains four tandem β-α repeat, represents the ideal Rossmann-like fold scaffold on which the C-PLoop was based^48^. Moreover, in comparison to C-PLoop, which exhibits conformational diversity, 2N3Z was shown to adopt a rigid and monomeric structure^48^.

**Fig. 9:**
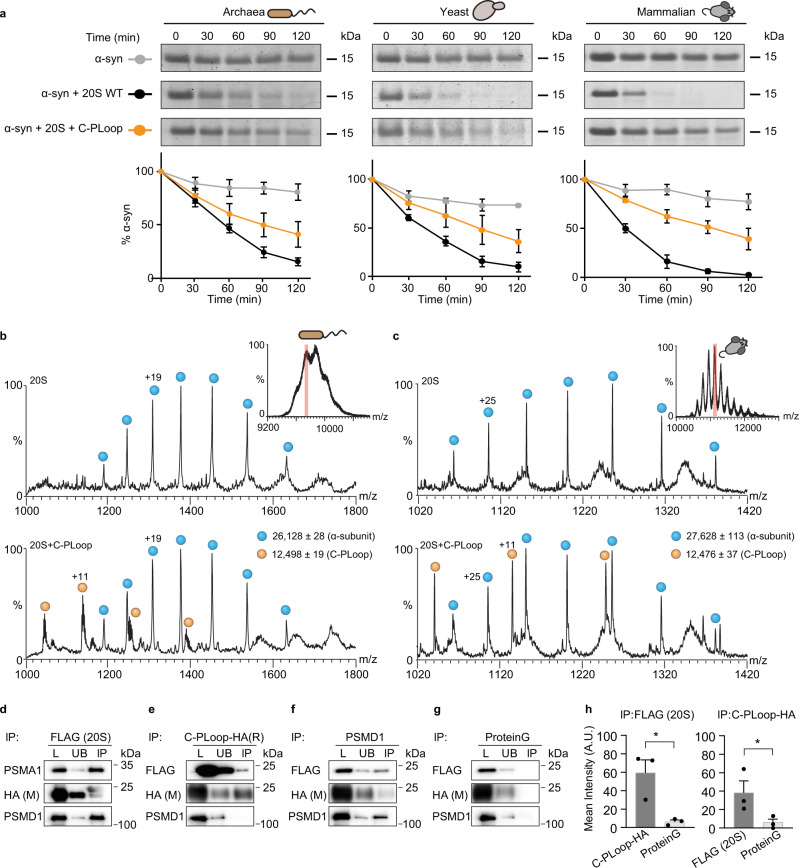
A de novo-designed C-PLoop protein binds the 20S proteasome. **a** To examine whether C-PLoop can protect α-synuclein (α-syn) from the 20S-mediated proteolysis, we incubated a mixture of α-syn and 20S proteasomes from archaea, yeast, and rat with C-PLoop to perform time-dependent degradation assays. Regardless of the 20S proteasome species, the addition of C-PLoop rescued the degradation of α-syn. Quantification of α-syn levels from three independent experiments is displayed on the bottom as mean intensities; error bars represent SD. Free **b** archaeal and **c** rat 20S proteasomes and 20S complexes pre-incubated with C-PLoop, were examined by native MS. For each sample, the most intense charge state obtained in the MS spectrum was subjected to MS/MS analysis (inset in (**b**) and (**c**) shows the MS spectrum of the free 20S proteasomes; the 65^+^ charge state highlighted in red was subjected to MS/MS analysis). Comparison of the free 20S spectrum (top panels **b**, **c**) with a mixture of the 20S proteasome and C-PLoop (lower panels **b**, **c**) revealed additional peaks that corresponded in mass to C-PLoop. By extrapolation, we can therefore conclude that prior to MS/MS analysis, C-PLoop binds to the 20S proteasome. Blue circles correspond to α-subunits of the archaea and rat 20S proteasomes; orange circles represent C-PLoop. **d**–**g** For cellular experiments, HA-tagged C-PLoop was overexpressed in HEK293T cells, stably expressing the FLAG-tagged PSMB2 subunit of the 20S proteasome. Lysates were subjected to IP using either **d** anti-FLAG-affinity gel, **e** anti-HA, or **f** anti-PSMD1 antibodies, or **g** uncoupled protein G beads as a control. Total starting lysate (L), unbound proteins (UB), and IP samples were analyzed by Western blot using anti-PSMA1, anti-FLAG, anti-HA, or anti-PSMD1 antibodies. **h** Bands corresponding to HA (i.e., C-PLoop) in the FLAG (20S) IP and FLAG (20S) in the HA IP were quantified and compared with the protein G control. Quantifications demonstrate the average of three independent experiments. Measurements were subjected to a one-tailed Student *t*-test analysis. Error bars represent SEM. * for IP:FLAG (20S) and IP:C-PLoop-HA represents *p-*values = 0.0350 and 0.0380, respectively. Source data are provided with this paper.

We continued by examining the ability of the three designed constructs, i.e., C-, E-PLoop, and 2N3Z, to protect the model substrate α-synuclein from 20S-mediated proteolysis. Only C-PLoop, and not 2N3Z, was able to act as a CCR and efficiently reduce the rate of α-synuclein degradation, whereas E-PLoop partially regulated the 20S proteasomal degradation (Supplementary Fig. 12b). Therefore, we considered only C-PLoop for further analysis, and showed that C-PLoop inhibited the degradation of α-synuclein by three different 20S proteasome orthologs isolated from rat (*Rattus norvegicus*), yeast (*S. cerevisiae*) and archaea (*T. acidophilum*) (Fig. 9a). This result not only indicates that C-PLoop seems to exhibit a generic CCR-like function, but it is also in accordance with our working model that a balance between structural flexibility/rigidity is required for it. A degree of structural stability is required for preventing self-proteolysis by the 20S proteasome (as seen for A-, B-, D-, and F-Loop proteins), whereas enhanced rigidity (as 2N3Z) precludes the structural transitions that are required for functionality.

The C-PLoop design is only 12.5 kDa rather than 20–31 kDa as the canonical CCR proteins. We therefore next examined whether this “partial-Rossmann” design can function in the same manner as CCRs. Initially, we determined whether C-PLoop physically binds the 20S proteasome. Tandem native MS analysis of C-PLoop mixed with either archaeal or rat 20S complex confirmed the physical binding of the construct to the proteasomes (Fig. 9b, c). This observation was further validated by immunoprecipitation (IP) experiments. HEK293T cells stably expressing the 20S proteasome PSMB2-FLAG subunit were transiently transfected with a C-terminal HA-tagged C-PLoop construct. Reciprocal IP with anti-HA and anti-FLAG confirmed the interaction, where the amount of 20S proteasome and C-PLoop being pulled down was significantly increased compared with the Protein G control (Fig. 9d–h and Supplementary Fig. 13a). Moreover, binding of C-PLoop to the 26S proteasome (PSMD1- a subunit of the 19S regulatory particle of the 26S proteasome) only occurred at low levels, indicating a preference for C-PLoop binding to the 20S proteasome, or to singly capped 26S proteasomes (Fig. 9e, f and Supplementary Fig. 13a). Overall, these results suggest that C-PLoop, like the CCR family, specifically binds to the 20S proteasome, rather than its 26S counterpart.

**Fig. 10:**
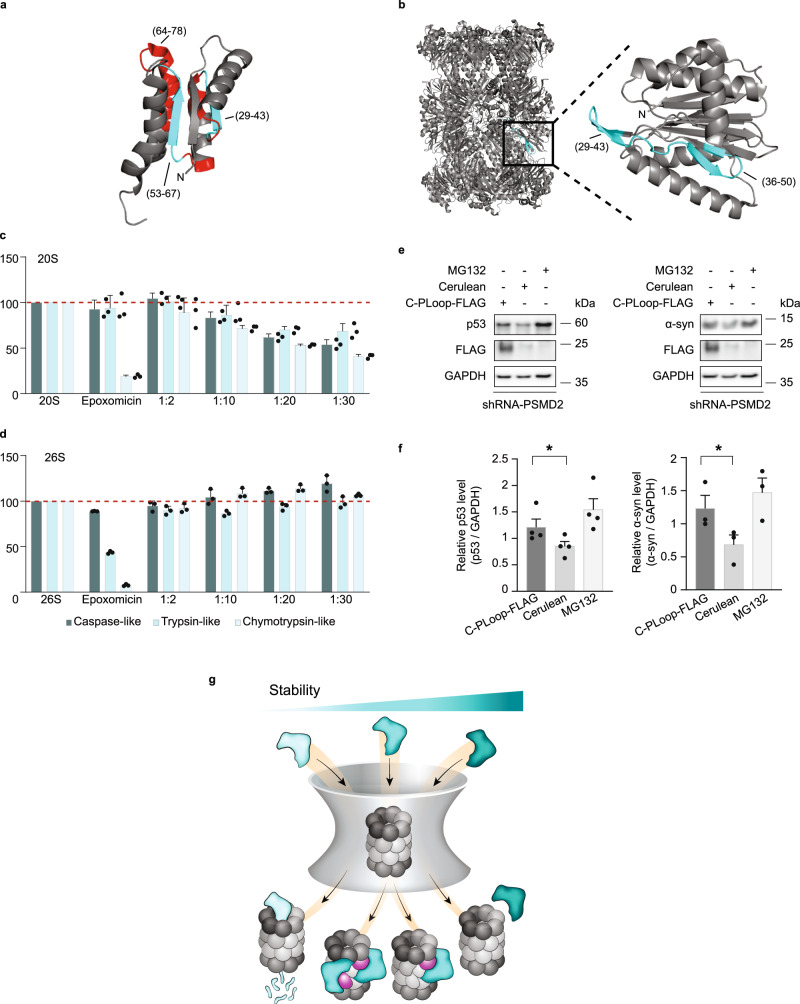
C-PLoop function similar to other CCRs. **a** 20S proteasomes binding region derived from peptide array is highlighted on the C-PLoop structure (PDB-6C2U). β-strands and α-helices are shown in cyan and red, respectively. **b** Peptide array-based C-PLoop binding peptides are mapped on the structure of the archaeal 20S proteasome β-subunit. An enlargement of the β-subunit binding site is shown in the inset. The catalytic activities of the human **c** 20S and **d** 26S proteasomes were measured in the presence of increasing concentrations of the C-PLoop. A concentration-dependent increase in inhibition of the 20S proteasome is detected, while the 26S proteasome was not inhibited. Bars represent mean values from three independent experiments; error bars represent SD. **e** Overexpression of C-PLoop-FLAG leads to increase in p53 and α-synuclein (α-syn) levels in T47D-760S cells. Cerulean was used as an overexpression control, and GAPDH as a loading control. **f** Quantification demonstrating the average of at least three independent experiments. Measurements were subjected to a one-tailed Student *t*-test analysis. Error bars represent SEM. * for p53 and α-syn represent *p-*values = 0.0318 and 0.0213, respectively. **g** CCRs act as allosteric regulators of the 20S proteasome. CCR (cyan) functions by binding to the PSMB4 subunit of the 20S proteasome (magenta). This interaction induces an allosteric structural transition within the 20S proteasome that perturbs the three enzymatic activities of the complex. We detected one CCR bound to the 20S proteasome; however, given the symmetrical architecture of the 20S proteasome, we cannot exclude the possibility that two CCRs bind, occupying the two PSMB4 subunits. A balance between structural rigidity and flexibility is required for CCR function. Increased structural flexibility will lead to CCR degradation by the 20S proteasome (bright cyan), like any other intrinsically unstructured protein, whereas a rigid CCR structure (dark cyan) will prevent the CCR/20S interaction. Source data are provided with this paper.

We were motivated to examine whether the interaction sites between C-PLoop and the 20S proteasome are identical to those discovered for CCRs, namely that an internal β-strand within C-PLoop binds to a β-loop-β secondary structure within the 20S proteasome. To explore this, a peptide array encompassing C-PLoop peptides was incubated with different 20S proteasomes from archaea, yeast, and humans (HEK293T cells) (Supplementary Fig. 14a–c, e and Supplementary Data 2). Remarkably, all proteasomes bound to a previously determined segment, namely a buried β-strand and an exposed α-helix of the C-PLoop (Fig. 10a and Supplementary Fig. 14b, c), similar to their binding motif in other CCRs (Fig. 3d).

In a reciprocal experiment, C-PLoop was incubated with an array composed of peptide sequences of the archaeal 20S proteasome (Supplementary Fig. 14d). Although a few peptides interacted with C-PLoop, interaction with peptides C1 and C2 (Supplementary Fig. 14d–g) correlated to the same binding site as identified for the CCRs CBR3 and NQO1 (Supplementary Fig. 5c–e and Fig. 6c), i.e., an exposed loop within the β-subunit (Fig. 10b). To determine whether the C-PLoop activity is specific for the 20S proteasome, or if it can also affect the activity of the 26S proteasome, peptidase activity was measured for both types of proteasomes in the presence of increasing concentrations of the protein. C-PLoop showed a concentration-dependent increase in inhibition of the 20S proteasome, while the activity of the 26S proteasome was not reduced (Fig. 10c, d). Taken together, these results indicate that the designed C-PLoop protein mimics both the CCR mode of interaction and specificity toward the 20S proteasome.

Next, we asked if C-PLoop, like other CCRs, can stabilize the cellular levels of 20S proteasome substrates. Taking into account that partly unfolded substrates can be sent to degradation via both the 20S and 26S proteasomes, it was necessary to clarify that the function of C-PLoop is specifically associated with the 20S and not the 26S proteasome degradation route. We therefore transiently transfected the C-terminal FLAG-tagged C-PLoop into the breast cancer cell lines T47D. Two lines of T47D cells were used, one of which expresses the red fluorescent protein (turboRFP) and harbors a doxycycline-inducible PSMD2 shRNA (a 19S subunit, so-called T47D-760S), and the second expresses the green fluorescent protein and is a control cell line (T47D-GFP)^50^ (Supplementary Fig. 13b, c). Under doxycycline treatment, the expression of PSMD2 is reduced, leading to decreased 26S proteasome levels and an increase in 20S complexes^50^, thus uncoupling the 26 and 20S proteasome degradation pathways. After 48 h of doxycycline treatment, C-PLoop-FLAG and cerulean plasmids were transfected into each T47D cell line. The proteasome inhibitor, MG132, was used as a control. Upon enhancement of the 20S proteasome levels and with the expression of C-PLoop-FLAG, a significant increase in α-synuclein and p53 levels was detected (Fig. 10e, f). Taken together, these data demonstrate that an artificial protein that harbors the CCR structural elements can act as an allosteric regulator of the 20S proteasome and validates our working model that the key determinant of CCR proteasome inhibition activity is a substructure within the Rossmann fold.

## Discussion

Emerging data have highlighted the biological significance of the 20S proteasome degradation route, which bypasses the ubiquitylation process, dependence on ATP, and binding to the 19S regulatory particle^6,7^. Diverse 20S proteasome substrates were identified, including ribosome-associated nascent polypeptides^51^, RNA binding proteins^52^, proteins that are damaged due to mutations or oxidation^17,53^, and proteins that in their native state contain regions that lack a well-defined folded structure^7,14,54^. Even proteins that are conjugated to poly-ubiquitin chains were shown to be degraded by the 20S proteasome^55^. In addition, multiple cellular processes were shown to be influenced by 20S-mediated degradation, such as neuronal stimulation^51,56^, antigenic peptide production^57,58^, hypoxia^55^, and post-translational processing^12,59–62^. Moreover, a variety of stress conditions were found to increase 20S proteasome levels, including oxidation stress^63–65^, nutrient starvation^66^ and hypoxia^55,67^. Up-regulation of the 20S complex, in terms of both abundance and activity, is also associated with extension of health span and life span^68,69^. Nevertheless, the signature activity of the 20S proteasome is not limited to the consequence of stress, as free 20S complexes were shown to outnumber capped 26S proteasome species under basal conditions across many different cell types^70–72^. Overall, these observations suggest that 20S-mediated degradation has a distinctive beneficial role separate from 26S proteasomes, and like any biological pathway, it requires tight regulation, but such a regulatory mechanism has not been studied in detail.

We discovered that the members of the CCR family, which have different cellular localizations and tissue specificities, specifically regulate the 20S proteasome function but not the 26S proteasome. These proteins, which inhibit the 20S proteasome at nanomolar concentrations, coordinate cellular levels of 20S proteasome substrates^20^. Here, we provide insight into the mechanism by which CCRs inhibit 20S proteasome activity. We show that CCRs do not block the proteasome orifice or compete with 20S proteasome substrates; rather, they act as allosteric regulators (Fig. 10g). For functioning, the CCRs require marginal stability of their core that comprises a Rossmann fold. Increased structural flexibility will lead to their degradation by the 20S proteasome, like any other intrinsically unstructured protein^43^, whereas a rigid core will prevent the CCR/20S interaction, as it is mediated by an internal β-strand that is exposed upon binding to the 20S proteasome β-ring. In particular, our results indicate that CCRs interact with a β-strand-loop structural element within the PSMB4 subunit. Although this is not a catalytic subunit of the proteasome, the interaction with PSMB4 allosterically reduces the caspase, chymotrypsin, and trypsin-like activities of the proteasome. We also show that this mode of inhibition can be recapitulated by a de novo-designed protein, C-PLoop, with a “partial-Rossmann” fold, opening up research opportunities for uncoupling the 26S and 20S proteasome degradation pathways.

The CCR family was discovered by searching for Rossmann fold proteins that contain a conserved N-terminal motif [MX_1-4_(K/R)_1-2_(V/L/I/A)_4_]. We found, however, that the N-terminal motif is not sufficient for the CCR/20S proteasome interaction (Supplementary Fig. 1). Nonetheless, it is important for maintaining the packing of the Rossmann fold, which in turn affects the CCR ability to inhibit the proteasome. Whether this sequence motif is critical for CCR functionality by means other than direct binding or only acts as a reporter of the Rossmann fold remains unclear at this point. If the latter is correct, however, it is likely that many more CCRs, which belong to folds other than Rossmann, remain unknown.

Allosteric regulation is most probably an integral aspect of the regulation of protein degradation by the proteasome. Prior studies demonstrated an allosteric pathway that traverses from the proteasome regulatory binding site at the outer surface of the α-rings to the proteolytic active sites within the proteasome inner chamber^73–79^. Similarly, cryo-EM structures of the 20S proteasome and the regulator PA200 revealed differential allosteric modulation of the individual proteasome proteolytic activities^80^. In these structures, rearrangements observed at the α-subunits are propagated allosterically into the β-subunits, resulting in different conformational changes at each of the proteolytic active sites. The phenomenon, as shown by a recent study, also extends across the entire 20S proteasome length (150 Å), wherein binding of an ATPase to one α-ring allosterically opens the gate at the opposite α-ring^81^. These structural transitions that propagate allosterically upon regulatory particle binding may explain why CCRs only bind to the free uncapped 20S proteasome. The capped 26S proteasome likely adopts a structural conformation that is not compatible with CCR binding.

Overall, our study unravels an unknown allosteric pathway that extends in a CCR-dependent manner from the non-active PSMB4 β-subunit to the catalytic subunits. This might be through the distinct C-terminal extension of the PSMB4 subunit that is inserted into the groove between the opposing PSMB6 and PSMB7 subunits, which possess the caspase- and trypsin-like activities, respectively^82^. Moreover, the pattern of proteolytic activity modulation upon interaction with PSMB4 may differ from one CCR to another^20^, suggesting that distinctive structural arrangements are triggered by individual CCRs.

Cereblon, a substrate adapter module of the Cullin 4 E3 ubiquitin ligase complex, has been shown to physically bind PSMB4, and like the CCR family, inhibit proteasome activity^24^. Similarly, a recent study demonstrated that the presynaptic scaffolding protein, bassoon, interacts directly with PSMB4 and attenuates the proteolytic activity of endogenous proteasomes^25^. These results may therefore hint at a more general role of PSMB4 as a proteasome regulator. In support of this notion, additional proteins have been shown to bind specifically to PSMB4. This was clearly demonstrated for the ubiquitin ligase SNEV, wherein the interaction with PSMB4 is conserved from yeast to mammalian cells^83^. Likewise, Smad1, which is involved in signaling the transforming growth factor β superfamily, binds PSMB4^84^. Viral proteins were also shown to bind PSMB4 as the HIV-1-derived protein Nef^85^ and the human T-cell leukemia virus protein, Tax^86^. These examples, together with our results, indicate that PSMB4 is a key site for proteasomal regulation and, consequently, a potential therapeutic target.

## Methods

### Plasmids and cloning

The genes for the PROSS and FuncLib designed variants of CBR3 and PROSS variant of PSMB4 subunit of the 20S complex were codon optimized for efficient expression in *E. coli* and mammalian cellular systems, respectively, and were custom synthesized as cDNA by Twist Biosciences. The following plasmids were acquired from Twist Biosciences pET29a-CBR3-P1 and P2, pET29a-CBR3-F1, F2 and F3, and pTWIST-CMV-PSMB4-HA. Plasmids encoding genes for α- and β-subunit of the *T. acidophilum* 20S proteasome pETM-11-alpha and pETM-60-NusA-beta respectively were obtained from Prof. Lewis Kay (University of Toronto). Purified PLoop proteins and the pET29b-C-PLoop plasmid encoding for C-PLoop-His were obtained from Prof. Dan Tawfik (Weizmann Institute of Science).

Following modifications were made to the plasmids: pET-15b DJ-1 was used as a template to delete amino acids ^4^KRALVIL^10^ at N-terminus to generate DJ-1_ΔN_ mutant using the primer pair: forward 5’-GCTAAAGGAGCAGAGGAAATG-3’ and reverse 5’-GGAAGCCATGGTATATCTC-3’.

pET29b-C-PLoop was used as a template to introduce an HA-tag at C-terminus using primer pair: forward 5’-CGCTAGCGCTACCGGTATGCGCGTTATCGTGGTGATC-3’, reverse 5’-GAAGCTTGAGCTCGAGCTACGCATAGTCAGGAACATCGTATGGGTAACCTC CGCCACGTGCTTTCGCAACTG-3’, and a FLAG-tag at C-terminus using primer pair: forward 5’-CGCTAGCGCTACCGGTATGC GCGTTATCGTGGTGATC-3’, reverse 5’-TAGATCCGGTGGATCCTCACTTGTCATCGTCATCCTTGTAGTCTCCTGCTCCTGCGCCACGTGCTTTCGCAACTG-3’ of the C-PLoop. The amplified products were inserted into the pHyg plasmid using an Infusion cloning kit (In-Fusion® HD, Takara).

The pHyg- Cerulean (Cer)plasmid was used as a template to generate CBR3-N-terminal-Cer using primer pair: forward 5’-AAGGAGATATACATATGTCGTCCTGCAGCCGCG TGGCGCTGGTGACCGGGGCCAACAGGGGCGGCGGCAGCGTGAGCAAGGGCGAGGAGCTG-3’, reverse 5’-GTGCGGCCGCAAGCTTTCACTTGTACAGCTCGTCC ATGC-3’. The amplified product was inserted into the pET28-HTEV plasmid.

The CBR3-N-terminus was amplified using primer pairs: forward 5’-GTGACTGGA GGCAACAAGGGCATCGGCTTGGCCATC-3’, reverse 5’-CAGCGCTACATGGA TGCCGG ACGACATGGATTGGAAGTAC-3,’ and the amplified product was inserted into pET28-HTEV-CBR1 to generate CBR1-(CBR3Nterm). The N-terminus of CBR1 was amplified using primer pairs: forward 5’-GTGACCGGGGCCAACAGGGGCATC GGCTTGGCCATC-3’, reverse 5’-CAGCGCCACGCGGCTGCAGGACGACATGG TACCCTG-3,’ and the amplified product was inserted into pNIC28-Bsa4 CBR3 to generate CBR3-(CBR1Nterm).

The pCDF1-NRas plasmid was used to generate NRas cancer-associated mutants using the following primer pairs: NRas-G12D forward 5’-GTTGGAGCAGATGGTGTTGGG-3’ and reverse 5’-CACCACCAGTTTGTACTCAG-3’, NRas-G13R forward 5’-TGGAGCAGGTCGTGTTGGGAA-3’ and reverse 5’-ACCACCACCAGTTTGTAC-3’, NRas-Q61R forward 5’-ACAGCTGGACGAGAAGAGTAC-3’ and reverse 5’-ATCCAGTATGTCCAACAAAC-3’. The site directed mutagenesis generated single-point mutant plasmids were then inserted into pET28-MHL plasmid.

All the NusA-PSMB-FLAG constructs were prepared by restriction-free cloning^87^, as follows. Initially, the beta subunit of the *T. acidophilum* 20S proteasome in pETM-60-NusA was replaced with human PSMB4-FLAG, using the forward 5’- AGGATGACGATGACAAGTAACTAGCTAGCTAGGGATCCGAATTC-3’ and reverse 5’- TGTAGTCTCCTGCTCCTGCTTCAAACCCAGATATCATATGAGCAATA-3’ primers. Following amplification of the plasmid, the linear PCR product was ligated using the KLD kit (M0554S, NEB). Next, PSMB4 was replaced by the same approach with each of the other human PSMB subunits, using the following primers;

PSMB1 forward 5’-CCGCGGGTGAGAATCTTTATTTTCAGGGCGCCATGTTGTCCTCTACAGCCATGTATTCG-3’.

PSMB1 reverse 5’- GTCATCGTCATCCTTGTAGTCTCCTGCTCCTGCGTCCTTCCTTAAGGAAACAGTTTCCTCC-3’.

PSMB2 forward 5’-CCGCGGGTGAGAATCTTTATTTTCAGGGCGCCATGGAGTACCTCATCGGTATCCAAGG-3’.

PSMB2 reverse 5’-GTCATCGTCATCCTTGTAGTCTCCTGCTCCTGCGGAGCCCTGTTTGGGGAAGGAAATG-3’.

PSMB3 reverse 5’-CCGCGGGTGAGAATCTTTATTTTCAGGGCGCCATGTCTATTATGTCCTATAACGGAGGGGC-3’.

PSMB3 reverse 5’-GTCATCGTCATCCTTGTAGTCTCCTGCTCCTGCGTCCATTCGGGCCTTCAGTGTCCTG-3’.

PSMB5 forward 5’-CCGCGGGTGAGAATCTTTATTTTCAGGGCGCCATGGCGCTTGCCAGCGTGTTGGAGAG-3’.

PSMB5 reverse 5’-GTCATCGTCATCCTTGTAGTCTCCTGCTCCTGCGGGGGTAGAGCCACTATACTTCTC-3’.

PSMB6 forward 5’-CCGCGGGTGAGAATCTTTATTTTCAGGGCGCCATGGCGGCTACCTTACTAGCTGCTC-3’.

PSMB6 reverse 5’-GTCATCGTCATCCTTGTAGTCTCCTGCTCCTGCGGCGGGTGGTAAAGTGGCAACGGC-3’.

PSMB7 forward 5’-CCGCGGGTGAGAATCTTTATTTTCAGGGCGCCATGGCGGCTGTGTCGGTGTATGCTCC-3’.

PSMB7 reverse 5’GTCATCGTCATCCTTGTAGTCTCCTGCTCCTGCGGAAGTGTCCATTGTTTGGACTGTTTC-3’.

All the plasmids were transformed into *E. coli* DH5α cells, and the DNA was extracted for sequencing to validate accuracy.

### Cell lines

HEK293T cells stably expressing the PSMB2 subunit of the proteasome, tagged with a C-terminal FLAG tag were obtained from Chaim Kahana (Weizmann Institute of Science). Wild-type HEK293T cells were obtained from Yosef Shaul (Weizmann Institute of Science). T47D cells harboring doxycycline-inducible control (T47D-GFP) and shRNA targeting PSMD2 (Rpn1 subunit of the 19S complex) (T47D-760S) cells were obtained from Peter Tsvetkov (Broad Institute of MIT and Harvard). Cell lines were not authenticated.

HEK293T cells were maintained in Dulbecco’s Modified Eagle’s Medium (Sigma), and T47D cells were maintained in RPMI-1640 (Biological Industries) medium supplemented with 10% fetal bovine serum (Gibco), 100 units/ml penicillin-100 µg/ml streptomycin (Biological Industries), 0.1 mM sodium pyruvate (Biological Industries), MEM-Eagle non-essential amino acids (Biological Industries) and MycoZap Prophylactic (Lonza) according to the manufacturer’s instructions. PSMB2-FLAG HEK293T cells were additionally supplemented with 1 mg/ml puromycin. HEK293T and T47D cells were grown in a humidified incubator at 37 °C with a 5% CO_2_-controlled atmosphere.

Yeast cells: The *S. cerevisiae* strain RJD1144, with a chromosomally FLAG-tagged β4 (PRE1) subunit generated in the lab of Raymond Deshaies, Caltech, California, USA, was used for the purification of yeast 20S proteasome^88^. The *S. cerevisiae* strain SUB544, generated in the lab of Michael Glickman, Technion-IIT, Haifa, Israel, which contains the PSMA4 subunit with deletion of residues (^2^GSRRYDSRT^10^) at the N-terminus, and C-terminus tagged with 6His-5Myc, was used for the purification of yeast open-gate (α3ΔN) mutant of the 20S proteasome^44^.

### Bacterial strains

The DH5α strain of *E. coli* was used for all plasmid cloning experiments. The BL21(DE3) strain of *E. coli* was used for all recombinant protein expression experiments in bacteria.

### Purification of DJ-1_WT_ and DJ-1_ΔN_

BL21(DE3) *E. coli* were transformed with pET-15b vector containing cDNA of human DJ-1 wild-type or DJ-1_ΔN_ mutant. Cells were grown in LB medium supplemented with 100 μg/ml ampicillin or 50 μg/ml kanamycin, respectively, at 37 °C until they reached OD_600_ 0.45. Protein expression was induced by the addition of 0.4 mM isopropyl-b-D-thiogalactoside (IPTG) for 2.5 h. Cells were harvested by centrifugation at 5000×*g* for 10 min and resuspended in 50 ml of 50 mM Tris-HCl pH 7.4, 2 mM EDTA, 1 mM DTT, 1 mM PMSF, and a protease inhibitor cocktail (Complete, Roche). Cells were lysed in a French Press, centrifuged for 10 min at 5000 × *g*, and the lysate was passed through a Source-15Q anion exchange 55 ml column (GE Healthcare) pre-equilibrated with 50 mM Tris-HCl pH 7.4, 1 mM DTT. After lysate loading, proteins were eluted with 200 ml of 50 mM Tris-HCl pH 7.4, 1 mM DTT. Then, 50 ml fractions were collected, and DJ-1-containing fractions (eluted after 150–200 ml) were concentrated using a 3-kDa Amicon Ultra column (Millipore). Concentrated DJ-1 was loaded onto a gel filtration column (Superdex 200, 10/300 GL, GE Healthcare), pre-equilibrated with 50 mM Tris-HCl pH 7.4, 300 mM NaCl, and 1 mM DTT.

### Purification of CBR3

BL21(DE3) was transformed either with pNIC28-Bsa4-CBR3 for wild-type or pET29b-CBR3 for PROSS(P) and FuncLib(F) mutants. Cells were grown in LB medium supplemented with 50 µg/ml kanamycin at 37 °C until they reached OD_600_ 0.6. Protein expression was induced by the addition of 1 mM IPTG for 3 h. Cells were harvested by centrifugation at 5000 × *g* for 10 min and resuspended in 20 mM sodium dihydrogen phosphate pH 7.4, 20 mM imidazole, 150 mM NaCl, 0.26 mM PMSF, 1 mM benzamidine, 1.4 µg/ml pepstatin. Cells were disrupted by the addition of 1 mg/ml lysozyme followed by rolling at 4 °C for 30 mins and sonication (40% amp, 30-s pulses for 7.5 min). The lysed cells were centrifuged at 18,000 × *g* for 45 mins at 4 °C to remove cellular debris. The supernatant was applied to a HisTrapHP column pre-equilibrated in 20 mM sodium dihydrogen phosphate pH 7.4, 20 mM imidazole, 150 mM NaCl, and a linear gradient to 400 mM imidazole over 40 ml was applied to elute the proteins. Fractions were analyzed by SDS-PAGE and Coomassie staining for CBR3-WT, PROSS, and FuncLib mutants. Fractions with proteins were collected, and the buffer was exchanged for 20 mM sodium dihydrogen phosphate pH 7.4 and 50 mM NaCl without cleaving the 6XHis-tag at the N-terminus. The purified proteins (Supplementary Fig. 15) were then concentrated, frozen in liquid N_2_, and stored at −80 °C. The purification of wild-type CBR3 with cleaved 6XHis-tag was purified, as explained before in ref. ^20^.

### Purification of PGDH and Ras proteins

BL21(DE3) transformed with pET28 6xHis-TEV PGDH, pCDF1-NRasA, pET28-MHL containing the NRas mutants, pET28-MHL KrasB (H61), pET28-HRas, were induced and purified as for CBR3 with the following changes. BL21(DE3) bacteria were transformed with pET28-PGDH containing a C-terminal 6xHis-tag. After elution from the first HisTrapHP column, fractions containing PGDH-6xHis were concentrated and loaded onto a gel filtration column (Superdex 200, 10/300 GL; GE Healthcare) pre-equilibrated with 20 mM sodium dihydrogen phosphate pH 7.4, and 50 mM NaCl. Fractions containing PGDH-6xHis were pooled, concentrated, frozen in liquid N_2_, and stored at −80 °C.

### Purification of C-PLoop

BL21(DE3) was transformed with pET29b-C-PLoop-6xHis and grown in LB medium supplemented with 50 µg/ml kanamycin at 37 °C until they reached OD_600_ 0.6^48^. Protein expression was induced by the addition of 1 mM IPTG for 5 h. Cells were harvested by centrifugation at 4000 × *g* for 15 min and resuspended in 50 mM sodium phosphate pH 7.4, 20 mM imidazole, 500 mM NaCl supplemented with 2 µl/ml EDTA-free protease inhibitor cocktail (Calbiochem), and 125 units/ml of benzonase (Merck). The cells were lysed by sonication with 35 % amp, 10-s pulse on and 2 min off until the lysate turned translucent. The lysate was cleared by centrifugation for 30 min, 18,000 × *g* at 4 °C. The supernatant was applied to the HisTrapHP column pre-equilibrated with 50 mM sodium phosphate pH 7.4, 20 mM imidazole, and 500 mM NaCl. C-PLoop-His was eluted with a linear gradient of 500 mM imidazole over 50 ml. Fractions containing C-PLoop-His were identified using 1:2500 anti-His antibody (A00174, Genscript), pooled and concentrated, and stored at −80 °C (Supplementary Fig. 15).

### Purification of archaeal 20S proteasome

The α and β subunits of *T. acidophilum* 20S proteasome were expressed as separate fusion proteins with a TEV-cleavable His tag (α) or with a NusA-His tag (β) in BL21 (DE3) cells. Expression of both subunits was induced with the addition of 1 mM IPTG, 37 °C for 3 h (α) or for 5 h (β) at 37 °C. Cells were collected by centrifugation at 5000×*g* for 20 min. Cells were lysed by sonication in 50 mM sodium phosphate buffer pH 8.0, supplemented with protease inhibitors (0.5 mM benzamidine, 0.1 mg/ml pepstatin A, and 0.1 µM PMSF), 0.88 mg/ml lysozyme, and 250 U benzonase (Millipore). After centrifugation at 40,000×*g* for 30 min, the supernatant was loaded onto a HisTrap FF (GE Healthcare) pre-equilibrated in 50 mM sodium phosphate buffer pH 8.0, 200 mM NaCl, and 10 mM imidazole. The α and β subunits were eluted in 100 mM sodium phosphate buffer pH 7.8, 300 mM imidazole. The fractions containing the fusion protein were pooled and dialyzed overnight with TEV protease against 50 mM Tris pH 7.4, 1 mM EDTA, and 2 mM DTT. Following the overnight TEV cleavage, the α and β subunits were loaded onto a HisTrap FF column, and flow-through fractions were collected. The full proteasome (α_7_β_7_ β_7_ α_7_) was assembled by mixing 1:1 molar ratio of α and β subunit and incubated at 37 °C for 6 h. The mixture was then concentrated to 0.5 ml and incubated overnight at 37 °C, followed by incubation at 60 °C for 1 hour. The assembled 20S proteasome complex was loaded onto a Superdex 200 10/300 GL (GE Healthcare) pre-equilibrated in 50 mM sodium phosphate buffer pH 7.5, 200 mM NaCl (Purity of the complex is shown in Supplementary Fig. 15). The assembly and purification of 20S proteasome complex under non-optimal conditions was performed as explained above without the incubation at 60 °C for 1 hour. The specific peptidase activity of the purified 20S proteasome was further activated by SDS (Supplementary Fig. 16), as documented for the latent form of 20S complexes^89^.

### Purification of yeast 20S proteasome

Yeast (*S. cerevisiae*) cells endogenously expressing PRE-1-FLAG, the yeast homolog for PSMB2 subunit, were grown in 4×700 ml YPD medium for 24 h at 30 °C. Cells were harvested at 5000×*g* for 20 min, the pellets were rinsed in 10 ml water, and centrifuged again at 5000×*g* for 20 min. The pellet was resuspended in 100 ml lysis buffer containing 50 mM Tris-HCl pH 7.5, 150 mM NaCl, 10% glycerol, 5 mM MgCl_2_, and 1 mM PMSF. Cells were lysed using a glass bead beater, pre-chilled with 50% glycerol and dry ice, with 1-min pulses for 7 min total. The lysed cells were separated from the glass beads and centrifuged at 35,000×*g* for 20 min at 4 °C to remove cell debris. The supernatant was collected and incubated with 2 ml anti-FLAG M2 affinity gel (Sigma), pre-rinsed with sequential washes of lysis buffer, glycine pH 3.5, and lysis buffer for 1.5 h at 4 °C while gently rotating. The beads were collected and washed sequentially with lysis buffer containing 0.2 % NP40, lysis buffer, and lysis buffer containing 500 mM NaCl. The last wash was incubated on the beads for 1 h at 4 °C, followed by a final wash in lysis buffer. 20S proteasomes were eluted using 500 μg/ml FLAG peptide in a lysis buffer containing 15 % glycerol. Purification was then validated by SDS-PAGE, and the activity of 20S was analyzed by fluorogenic substrate Suc-LLVY-7-amino-4-methylcoumarin (AMC) (Boston Biochem).

### Purification of α3ΔN open-gate yeast 20S proteasome

Yeast (*S. cerevisiae*) cells endogenously harboring a deletion of N-terminus on α_3_(PSMA4)-subunit were grown in 4 × 700 ml YPD medium for 24 h at 30 °C. Cells were harvested at 5000 × *g* for 20 min, and the pellets were rinsed in 10 ml water and centrifuged again at 5000×*g* for 10 min. Cells were resuspended in lysis buffer containing 20 mM Tris-HCl pH 7.5, 0.5 mM EDTA, 10% Glycerol, and 1 mM DTT and lysed in a pre-cooled French press for three cycles at 20,000 psi. The cell debris was removed by centrifugation at 16,000 × *g* for 15 min, and the resulting supernatant was cleaned by ultracentrifugation at 150,000×*g* for 1 h, and lipids were cleared by passing the supernatant through the glass wool. The collected supernatant was applied to HiTrap DEAE 5 ml column (GE Healthcare) pre-equilibrated with 20 mM Tris-HCl pH 7.5, 0.5 mM EDTA, 10% Glycerol, and 1 mM DTT. The proteasomes were eluted with pre-equilibration buffer supplemented with 250 mM NaCl. Eluted fractions were tested for the hydrolysis activity of the Suc-LLVY-AMC peptide, and fractions with activity were pooled. The pooled fractions were applied to Resource Q column (GE Healthcare) pre-equilibrated with 20 mM Tris-HCl pH 7.5, 0.5 mM EDTA, 10% Glycerol, 1 mM DTT, and 250 mM NaCl, and proteasomes were eluted as 1 ml fractions with 450 mM NaCl. The eluted fractions were tested for proteolytic activity, active fractions were pooled, and buffer was exchanged for lysis buffer with 10% glycerol and concentrated to store at −80 °C.

### Purification of human 20S proteasome

For the purification of human 20S proteasome, HEK293T cells stably expressing the PSMB2-FLAG subunit were plated in 20×15-cm dishes and grown for 48 h with DMEM medium and puromycin (1 µg/ml). Cells were collected by trypsinization, washed in PBS, and stored until the lysis. Cells were resuspended in 5 ml of lysis buffer with 50 mM of Tris pH 7.5, NaCl 150 mM, NP40 0.5%, and 5 mM MgCl_2_ and incubated for 10 min at 4 °C rotating. Cells were then homogenized in a glass-Teflon homogenizer for 10 strokes, and the lysate was cleared by centrifugation at 14,000 × *g* for 10 min. The subsequent purification steps were similar to the yeast 20S proteasome purification, as explained above, using anti-FLAG M2 affinity gel (Sigma) (Purity of the complex is shown in Supplementary Fig. 15). The specific peptidase activity of the purified 20S proteasome was further activated by SDS (Supplementary Fig. 16), as documented for the latent form of 20S complexes^89^.

### Purification of human 26S proteasome

26S proteasomes from HEK293T cells were purified as described for the human 20S proteasome, with some changes. To keep the 26S proteasome intact, all buffers were supplemented with 1 mM ATP, 5 mM MgCl_2_, 10% glycerol, and an ATP regenerating system containing 50 μg/ml creatine kinase and 2.5 mM creatine phosphate. In addition, the wash and incubation in 500 mM NaCl steps were omitted from the protocol. The purity of the complex is shown in Supplementary Fig. 15.

### Purification of mammalian 20S proteasome from rat liver

Rat livers were harvested from *Rattus norvegicus* strain RCS for the purification of the mammalian 20S proteasomes. Purification of the rat 20S proteasome was performed as described previously^26^. In brief, rat livers were homogenized in a buffer containing 20 mM Tris-HCl pH 7.5, 1 mM EDTA, 1 mM DTT, and 250 mM sucrose. The extract was subjected to centrifugation at 1000 × *g* for 15 min. The supernatant was then diluted to 400 ml to a final concentration of 0.5 M NaCl and 1 mM DTT and subjected to ultracentrifugation for 2.2 h at 145,000 × *g*. The supernatant was centrifuged again at 150,000 × *g* for 6 h. The pellet containing the proteasomes was resuspended in 20 mM Tris-HCl pH 7.5 and loaded onto 1.8 L Sepharose 4B resin. Fractions containing the 20S proteasome were identified by their ability to hydrolyze the fluorogenic peptide suc-LLVY-AMC, in the presence of 0.02% SDS. Proteasome-containing fractions were then combined and loaded onto four successive anion exchange columns: Source Q15, HiTrap DEAE FF, and Mono Q 5/50 GL (GE Healthcare). Elution was performed with a 0–1 M NaCl gradient. Active fractions were combined, and the buffer was exchanged to 10 mM phosphate buffer pH 7.4 containing 10 mM MgCl2 using 10 kD Vivaspin 20 ml columns (GE Healthcare). Samples were then loaded onto a CHT ceramic hydroxyapatite column (Bio-Rad Laboratories Inc.); a linear gradient of 10–400 mM phosphate buffer was used for elution. The purified 20S proteasomes were analyzed by SDS-PAGE, activity assays, and MS analysis. The specific peptidase activity of the purified 20S proteasome was further activated by SDS (Supplementary Fig. 16), as documented for the latent form of 20S complexes^89^. It is also noteworthy that the same batch of purified 20S proteasomes was used for all repeats of a particular experiment. Ethical approval to work with rat livers is not required since livers for proteasome purifications were collected only from redundant rats that were terminated since they were no longer required for scientific experiments. The sex of the animals is insignificant to our study.

### Purification of 20S proteasomes with PSMB4-HA WT and des

For this, 15×15-cm dishes with wild-type HEK93T cells were grown up to ~70% confluency and then transiently transfected with 20 µg of pTWIST-CMV-PSMB4-HA plasmid. Media was changed and supplied with hygromycin (0.1 mg/ml) 5 h post-transfection, and cells were collected after 24 h by trypsinization and stored in -80°C. Cells were resuspended in 5 ml of lysis buffer with 50 mM of Tris pH 7.5, NaCl 150 mM, NP40 0.5%, and 5 mM MgCl_2_ and incubated for 10 min at 4 °C rotating. Cells were then homogenized in a glass-Teflon homogenizer for 10 strokes, and the lysate was cleared by centrifugation at 14,000 × *g* for 10 min. The supernatant was collected and incubated with 1 ml anti-HA agarose (Pierce), pre-rinsed with sequential washes with Tris-buffered saline (TBS) and lysis buffer for 2 h at 4 °C while gently rotating. The beads were collected and washed sequentially with TBS containing 0.05% Tween 20, lysis buffer, and lysis buffer containing 500 mM NaCl. The last wash was incubated on the beads for 1 h at 4 °C, followed by a final wash in lysis buffer. 20S proteasomes were eluted with 1 mg/ml HA peptide in TBS buffer after incubating at 30 °C for 10 min. Purification was then validated by SDS-PAGE and Western blot using antibodies against HA-tag. The assembly of the PSMB4-HA variant into the 20S complex was confirmed by SDS-PAGE, followed by Western blot detection and monolithic column-based mass spectrometry analysis (Fig. 8c and Supplementary Figs. 9, 10).

### Native mass spectrometry analysis

For CBR3 and archaeal 20S binding experiments (Fig. 5a–e), 0.3 µM of 20S and 25 µM of CBR3 were buffer exchanged to 150 mM ammonium acetate pH 7.5 separately and then mixed to incubate on ice for ~5 h followed by MS and tandem MS analysis on a modified Q-Exactive Plus Orbitrap EMR (ThermoFisher Scientific). Typically, aliquots of 2 µl of the sample were electrosprayed from gold-coated borosilicate capillaries prepared in-house^90^. Experiments were performed in positive ion mode, and conditions were optimized to ionize and remove the adducts without disrupting the non-covalent interactions of the proteins tested. In MS/MS experiments, the relevant m/z values were isolated, and argon gas was admitted to the collision cell. Spectra are shown without smoothing or background subtraction. The following experimental conditions were used on the Orbitrap EMR: capillary voltage 1.7 kV, MS spectra were recorded at resolution 10,000, and the HCD cell voltage was set to 1–20 V at a trapping gas pressure setting of 3.9. For MS/MS analyses, a wide isolation window for the different assemblies of the archaeal 20S proteasome was set in the quadrupole, allowing the transmission of only high m/z species. Transmitted ions were subjected to collision-induced dissociation in the HCD cell at an accelerating voltage of 150 V, and the trapping gas pressure was set to 1.5.

The C-PLoop binding experiments with rat and archaeal 20S proteasomes (Fig. 9b, c) were performed on a QToF Q-Star Elite instrument (MDS Sciex, Canada), modified for improved transmission of large non-covalent complexes. Then, 25 µM of C-PLoop was mixed with 0.5 µM of the 20S proteasomes and incubated on ice for 1 h in 50 mM HEPES pH 7.5 and buffer exchanged to 500 mM ammonium acetate pH 7.5 before performing native MS experiments. In tandem MS analysis, the relevant m/z values were isolated, and collision-induced dissociation was performed. The following experimental parameters were used: capillary voltage 1.1 kV, declustering potential 200 V, focusing potential 200 V, a second declustering potential of 15 V, and collision energy between 20 and 125 V.

For structural characterization of purified DJ-1 WT, DJ-1 ΔN (Supplementary Fig. 1a), and NRas-WT, NRas-G12D, NRas-G13R, and NRas-Q61R (Supplementary Fig. 3c), each protein was used at 20 µM and buffer exchanged to 500 mM ammonium acetate pH 7.5. Typically, aliquots of 3 µl of the sample were electrosprayed into modified Q-Exactive Plus Orbitrap EMR with the following native conditions: HCD cell at 20 V, inject flatapole 2 V, bent flatapole 1.8, resolution 17,500, and the trapping gas pressure was set to 20 V.

To determine the stoichiometry of CCR binding to the 20S proteasome (Fig. 5e–j, Supplementary Fig. 6), native MS experiments were conducted using the UHMR Orbitrap instrument (Thermo Fisher Scientific). Instrumental parameters were set as follows: Capillary temperature 250 °C, capillary voltage 1.2 kV, trapping gas pressure 7, corresponding to an FV of 1.62 mbar, HV pressure of 2.6 × 10^−4^ mbar, and a UHV pressure of 2.7 × 10^−10^ mbar. The detector m/z optimization was set to low, and the ion transfer target m/z was set to high, with no desolvation and HCD voltages. The bent flatapole DC bias and gradient were set to 2.0 and 30 V. For the detection of the free CCRs, the trapping gas pressure was lowered to 1.

### Direct MS analysis

For direct MS analysis, *E. coli* BL21(DE3) expressing CBR3 wild-type or PROSS(P) and FuncLib(F) mutants (Supplementary Fig. 3b, e) were grown in 30 ml of LB medium supplemented with 50 µg/ml kanamycin at 37 °C until they reached OD_600_ 0.6^91^. Protein expression was induced by the addition of 1 mM IPTG for 3 h. Cells were harvested by centrifugation at 5000×*g* for 5 min, and pellets were washed once with 150 mM ammonium acetate pH 7.5 to remove residual growth medium. Cells were then resuspended in 1 M ammonium acetate pH 7.5 with protease inhibitors 0.26 mM PMSF, 1 mM benzamidine, 1.4 µg/ml pepstatin and lysed by sonication at 35% amp, with 5 s on and 25 s off cycle for 10 min. The lysate was cleared by centrifugation at 15,000×*g* for 10 min at 4 °C to remove cellular debris, and the supernatant was flash-frozen until the direct MS analysis.

MS analysis of the above supernatant was performed on a modified Synapt-G1 instrument with the following parameters: capillary voltage 1.5 kV, sampling cone 25 V, extraction cone 5 V, trap and transfer collision energies 50 and 20 V, respectively. Nitrogen was used as gas, with ion mobility (IM) wave velocity set to 350 m/s and wave height set to 15 V.

### Reversed-phase LC-MS analysis

20S proteasome samples, HA-affinity purified from HEK293T cells (Supplementary Fig. 10), were separated on a home-made reversed-phase monolithic column^92^ and eluted over a gradient of 29–41% acetonitrile, during 10 min, at 60 °C. Eluted proteins were directly sprayed into the UHMR Orbitrap mass spectrometer for accurate mass analysis using the HESI source. Instrumental parameters were set as follows: Capillary temperature 250 °C, capillary voltage 4 kV, sheath gas 3, auxiliary gas 10. Trapping gas pressure was set to 1, corresponding to an FV of 1.7 mbar, an HV pressure of 3.0 × 10^−5^ mbar, and a UHV pressure of 1.0 × 10^−10^ mbar. The detector m/z optimization and the ion transfer target m/z were set to low, with no desolvation and HCD voltages.

### MS data analysis

Data analysis for Fig. 5f–j was performed using the computational suite UniDec v. 4.1.145^93^. Deconvolution was performed in the m/z range between 11,000–13,500, covering all charge states in the series without smoothing. Mass ranges were set to 710,000–760,000 Da, and mass sampling was set to 4 Da, with a peak full width at half-max of 13 m/z, using a Gaussian peak shape function. The peak detection range was set to 200–300 Da, and the peak detection threshold to 0.01. Every measured mass in UniDec was manually inspected to ensure the correct assignment. Reported masses were manually averaged from measured mass values derived from 3–4 spectra for Fig. 5f–j. Errors represent SD. Data analysis for all other spectra was performed using the MassLynx software (Waters V4.2 SCN982). Reported masses were averaged by the software for the entire charge series in each spectrum. Errors represent SD. All theoretical masses, measured masses, and mass errors are shown in Supplementary Table 3.

### Ni-NTA binding assay

For this, 50 µM of CBR3 was incubated with 2 µM purified and non-assembled either α- or β-subunit of the archaeal 20S proteasome (*T. acidophilum*) with 25 mM HEPES pH 7.5 in a final volume of 130 μl for 1 h on ice. The mixture was loaded onto a HisTrap FF (GE Healthcare) pre-equilibrated in 50 mM sodium phosphate buffer pH 8.0, 200 mM NaCl, and 10 mM imidazole through a 100 μl inlet loop using a Hamilton syringe. The bound proteins were eluted using 100 mM sodium phosphate buffer pH 7.8, 300 mM imidazole over 50 ml with 25 fractions. Fractions with UV absorption were separated on 15% SDS-PAGE, followed by the detection of CBR3 and His-tag at the C-terminus of the α- and β-subunits after Western blot transfer.

### Cryo-EM sample preparation and data collection

For this, 6 mg/ml of purified endogenous rat liver 20S proteasome was incubated on ice for 1 h with CBR3 at 1:50 molar ratio. Then, 2.5 µl of this mixture was applied to C-flat 2/2 300 mesh holey carbon grids (Protochips). Grids were blotted for 3 s at 4 °C and 100% humidity and plunge frozen to liquid ethane cooled by liquid nitrogen using a Vitrobot automated plunger (Thermo Fisher Scientific). The sample was applied to the grids 30 min after glow discharge to improve the percentage of side views. Titan Krios G3i electron microscope (Thermo Fisher Scientific) operating at 300 kV was used for the imaging at a nominal magnification of ×47,000, corresponding to a pixel size of 1.7 Å. A total of 596 movies were recorded on a Falcon 3EC direct electron detector (Thermo Fisher Scientific) using automated acquisition in EPU (v 2.5) software (Thermo Fisher Scientific). A nominal defocus range of −1.0 to −2.0 µm was used to collect the movies, and each movie was fractionated into 20 frames. The dose rate was set to ~0.96 e^-^/pixel/s, and the total exposure time was 60 s, corresponding to an accumulated dose of ~20 e^-^/Å^2^.

### Cryo-EM image processing

RELION 3.0 was used for image processing^94^. A total of 596 micrographs were subjected to motion correction with 5 × 5 patches and dose-weighted using MotionCor2, followed by CTF estimation using CTFFIND4^95^. Further processing was performed for images showing well-defined particles and thin ice. Approximately 1122 particles were manually picked and subjected to reference-free 2D classification. The generated class averages were used as a template for autopicking. A total of 55,555 particles were autopicked from the selected images, extracted without binning, and subjected to another round of 2D classification to clean the dataset, resulting in 55,403 particles. This was followed by 3D autorefine without any symmetry using the map of the endogenous human constitutive proteasome from the red blood cells^96^, low-pass filtered to 40 Å. Further, two rounds of 3D classification without any symmetry cleaned the dataset resulting in 53,138 particles, from which 30,273 particles did render ~12 Å map with extra densities at β-rings of the 20S proteasome (Supplementary Fig. 7B).

### Superimposition of the density map

The cryo-EM structure of rat 20S proteasome (PDB:6TU3)^46^ was used to superimpose the electron density map of the 20-CBR3 complex. The C-terminus of PSMA3 and PSMA7 subunits can be distinctively recognized in the 20S-CBR3 complex EM map, and for 6TU3, therefore it was used as an anchor point for the superimposition. The CBR3 interacting subunit on the 20S proteasome was determined after superimposition by proximity to the extra density using UCSF Chimera 1.16^97^. Electron density maps for the 20-CBR3 complex are presented with a soft mask (volume hide dust size=30.1) and displayed at a lower contour level (0.0022) using UCSF Chimera 1.16 in order to visualize the extra densities on the β-rings in Fig. 7a.

### Immunoprecipitation

HEK293T cells stably expressing the PSMB2-FLAG proteasome subunit were plated in four 15-cm dishes at a density of 1.5 × 10^6^ cells per dish. Each plate was transfected with 10 µg of pHyg-C-PLoop-HA and grown for 48 h. Cells were collected by trypsinization, combined, washed in PBS, and resuspended in 1 ml lysis buffer (20 mM HEPES pH 7.4, 10 % glycerol, 10 mM NaCl, 3 mM MgCl_2_, 1 mM ATP) and protease inhibitors (1 mM PMSF, 1 mM benzamidine, 1.4 µg/ml pepstatin). Cells were incubated on ice for 15 min and homogenized in a glass-Teflon homogenizer for 40 strokes. Lysate was cleared by centrifugation at 18,000 × *g* for 10 min at 4 °C. For IP using anti-FLAG affinity gel, 0.5 mg protein was diluted in 500 µl lysis buffer. NaCl concentration was adjusted to 150 mM and rotated overnight at 4 °C in the presence of 45 µl anti-FLAG M2 affinity gel (Sigma). The following morning, beads were washed three times with lysis buffer containing 150 mM NaCl and boiled in 55 µl reducing sample buffer. For IP using anti-HA or anti-Rpn2 antibodies, 0.5 mg protein was diluted in 500 μl lysis buffer. NaCl concentration was adjusted to 150 mM. Proteins were pre-cleared using 40 μl of Protein G Sepharose (GE Healthcare), for 1 h at 4 °C, at a gentle rotation. The beads were discarded, and the lysate was rotated overnight at 4 °C in the presence of 9 μl anti-HA rabbit (ab9110, Abcam) or 9 μl anti-Rpn2 (PSMD1, ab140682, Abcam) antibody. The following morning, 45 μl Protein G Sepharose beads (GE Healthcare) were added, and the lysate was rotated for 2 h at 4 °C. The beads were then washed three times in lysis buffer containing 150 mM NaCl and boiled in 55 μl protein sample buffer.

For the immunoprecipitation experiments with wild-type/PROSS designed PSMB4 and CBR3, HEK293T WT cells were plated at 1.5 × 10^6^ cells in 15-cm dishes. Cells were co-transfected with 20 µg of either pTWIST-CMV-PSMB4-WT-HA (PSMB4-WT) or pTWIST-CMV-PSMB4-des-HA (PSMB4-des) and pCDF1-CBR3-WT plasmids and grown for >24 h with selective resistance. Subsequently, cells were collected by trypsinization, combined, washed in PBS, and resuspended in 1 ml lysis buffer (20 mM HEPES pH 7.4, 10 % glycerol, 10 mM NaCl, 3 mM MgCl_2_, 1 mM ATP) and protease inhibitors (1 mM PMSF, 1 mM benzamidine, 1.4 µg/ml pepstatin). Cells were incubated on ice for 15 min and homogenized in a glass-Teflon homogenizer for 40 strokes. Lysate was cleared by centrifugation at 18,000 × *g* for 10 min at 4 °C. For IP using anti-HA and Protein G resin, 1 mg protein was diluted in 500 µl lysis buffer. NaCl concentration was adjusted to 150 mM and rotated overnight at 4 °C in the presence of 50 µl of anti-HA agarose (Thermo Scientific) and Protein G Sepharose (GE Healthcare). The following morning, beads were washed one time with lysis buffer containing 150 mM NaCl and boiled in 50 µl reducing sample buffer.

### β-subunits/CBR3 pull-down assay

FLAG and NusA-tagged PSMB1-PSMB7 and His_6_-CBR3 were expressed in *E. coli* (BL21) by induction with 1 mM IPTG for 3 h at 37 °C. Cell pellets were washed in buffer A (50 mM PBS pH 7.4, 200 mM NaCl, 20 mM imidazole) and snap-frozen in liquid nitrogen. Cells were resuspended in 1 ml buffer A and lysed by sonication (10 min at 35% amplitude, 5 s on and 25 s off cycles), and the non-soluble fraction was cleared by centrifugation (14 kg, 10 min).

Next, 30 μg CBR3 lysate was coupled to 15 μl Ni-beads in 500 μl buffer A for 1 h at 4 °C, then washed three times in 500 μl buffer A and incubated with FLAG-tagged β-subunit lysate (50 μg in 500 μl) for an additional hour under the same conditions. Unbound proteins were removed by two washes (500 μl each) in buffer A, then with the same buffer containing 50 mM imidazole. CBR3 and bound proteins were co-eluted in 50 μl buffer B (50 mM PBS pH 7.4, 200 mM NaCl, 160 mM imidazole). For non-specific binding control lysates of each β-subunit were incubated with the beads without CBR3.

Elution and load fractions (50 μl) were diluted with 12.5 μl 5x Laemmli sample buffer and run in 12% SDS-PAGE for Western blotting and Coomassie staining, respectively. Blots were then analyzed by densitometry quantification (anti-FLAG 1:5000, ab1162 Abcam). The pulled-down PSMB subunits were compared after subtraction of the non-specific binding of each β-subunit lysate (beads without bound CBR3) and normalized to their levels in the corresponding load fraction.

### Measuring cellular substrate levels with C-PLoop-FLAG overexpression

Human breast cancer T47D cells harboring doxycycline-inducible control (T47D-GFP) and shRNA targeting PSMD2 (Rpn1 subunit of the 19S complex) (T47D-760S) were treated with 1 μg/ml of doxycycline for 48 h to induce knockdown of the PSMD2. Cells were then transfected with 4 μg of C-PLoop-HA and Cerulean control plasmid. Twenty-four hours post-transfection, the cells were collected and lysed in modified RIPA buffer containing 50 mM HEPES pH 7.6, 150 mM NaCl, 1% NP40, 0.25% sodium-deoxycholate, 0.26 mM PMSF, 1 mM benzamidine, and 1.4 µg/ml pepstatin. Cell debris was removed by centrifugation at 10,000 × *g* for 10 min, and the supernatant was collected. Total protein concentration was estimated by Bradford assay, and 30 μg of total protein was loaded for each sample. α-synuclein and p53 levels were detected by anti-α-synuclein (1:500, ab51252, Abcam) and anti-p53 HRP (1:2500, HAF1355, Biotest) antibodies respectively. Band intensities were measured by ImageJ.

### Kinetic assays

Kinetic assays were performed using rat 20S proteasome (0.3 μM) with and without CBR3, PGDH at 5 and 10 μM incubated on ice for 1 h. Subsequently, PSMB5 subunit (chymotrypsin-like) substrate Suc-LLVY-AMC was added at concentrations 6.25, 12.5, 25, 50, 100, 200, and 400 μM, mixed well, and immediately the AMC hydrolysis was measured for 20 min with 1 min interval using a microplate reader (Infinite 200, Tecan Group), using an excitation and emission filter of 360 nm and 460 nm respectively. A similar procedure was followed for substrate saturation curve experiments, except the AMC hydrolysis was measured for additional concentrations 6.25, 12.5, 25, 50, 75, 100, 125, 150, 200, 300, and 400 μM and up to 10 min with 1 min interval. The rate of AMC hydrolysis was plotted and line fit using nonlinear regression and the Michaelis–Menten kinetics equation (GraphPad Prism V6) to derive *V*_*max*_ and *K*_*m*_ values.

For Lineweaver–Burk double reciprocal plot initial enzymatic rate (*V*) of the 20S proteasome was derived as a slope, using arbitrary fluorescence units over the time for each concentration of the substrate in the presence and absence of CCRs. The inverse values of the initial rate (1/*V*) and the substrate concentration (1/*S*) were used to make the plot in Microsoft Excel 2016.

### Proteasome activity assays

Proteasome activity assays were performed as previously described^26^. In brief, for endpoint assays, 0.4 µM rat 20S proteasomes were incubated either alone or with 10 µM CCRs CBR3 and PGDH in 25 mM HEPES pH 7.5 for 1 h on ice. Then, 4 µl of the mixture was combined with 40 µl 25 mM HEPES pH 7.5 containing 50 µM either of Suc-LLVY-AMC, Z-LLE-AMC, and Boc-LRR-AMC substrates for PSMB5, PSMB6, and PSMB7 active subunits respectively. Samples were taken to measure the fluorescence of hydrolyzed AMC groups with a microplate reader for 30 min (Infinite 200, Tecan Group), using an excitation filter of 360 nm and an emission filter of 460 nm. A similar procedure was followed to measure the AMC hydrolysis of Suc-LLVY-AMC by the 20S complexes with PSMB4-WT and PSMB-des subunit at equimolar concentrations incubated either with MG132 at 6.25 µM or PSMC3 C-terminus peptide at 100 µM.

For CCR dose-response assays, 0.2 μM rat 20S proteasomes were incubated either alone or with 0.4 μM (2x), 2 μM (10x), 4 μM (20x), or 6 μM (30x) PGDH or C-PLoop, in 25 mM HEPES, pH 7.5 for 1 h on ice. Then, 4 μl of the mixture was mixed with 40 μl 25 mM HEPES pH 7.5 containing 100 μM either of Suc-LLVY-AMC, Z-LLE-AMC, or Boc-LRR-AMC substrates for PSMB5, PSMB6, and PSMB7 active subunits, respectively. Samples were incubated for 30 min at 37 °C, and then, the reaction was quenched by the addition of 150 μl 1% SDS. The fluorescence of the hydrolyzed AMC groups was measured on a microplate reader (Infinite 200, Tecan Group) using an excitation filter of 360 nm and an emission filter of 460 nm.

### CBR3 activity assay

For this, 10 µg and 50 µg of CBR3 PROSS and FuncLib mutants were used to reduce 4 mg of Isatin (Sigma) respectively. The absorbance of NADPH was measured using a microplate reader (Infinite 200, Tecan Group) at 340 nm as the activity of the CBR3 for 60 min and 15 min with 30 s of interval for PROSS and FuncLib mutants respectively.

### Degradation assays

To monitor the ability of CCRs and their respective mutants to regulate the activity of the 20S proteasome in vitro, 10 µM of the proteins were pre-incubated with 0.3 µM of the rat 20S proteasome or yeast wild-type and open-gate (α3ΔN) mutant 20S complexes for 1 h on ice in 50 mM HEPES pH 7.5. To initiate the assay, α-synuclein (α-syn) was added to 1 µM, and the reaction mixtures were incubated at 37 °C. For experiments to induce the gate-opening, the CCRs and 20S proteasome mixture was incubated with 150 µM of PSMC3 C-terminus peptide for 20 min at 37 °C or 100 µM Chlorpromazine (CPZ) for 1 h on ice before adding α-syn. Then, 10 µl samples were taken every 30 min for 120 min, quenched by the addition of SDS sample buffer, and snap-frozen in liquid N_2_. After all time points were collected, the samples were thawed, boiled for 5 min, and loaded onto a 15 % SDS-PAGE gel. Gels were stained with Coomassie brilliant blue, and changes in the level of α-syn were quantified by band densitometry using ImageJ 1.51k, R v.4, normalized to T0, and plotted using Microsoft Excel 2016.

A similar procedure was followed for C-PLoop, except the mixture was incubated at the following temperatures 60 °C, 30 °C, and 37 °C for archaea (*T.acidophilum*), yeast (*S. cerevisiae*), and rat (*R. norvegicus*) 20S proteasomes respectively. Further stability of other DJ-1_WT_, DJ-1_ΔN_, and P-Loop constructs was analyzed as explained above without adding the substrate α-synuclein.

Activation of the latent proteasome was examined by the addition of SDS. In this experiment, 20S proteasomes (0.2 μM) were incubated with 1.5 μM α-synuclein (α-syn) in 50 mM HEPES, pH 7.5, either with or without 0.02% SDS, at 37 °C. Then, 10 μl samples were taken every 30 min for 120 min, quenched by the addition of SDS sample buffer, and snap-frozen in liquid N_2_. Samples were then boiled for 5 min and loaded onto a 15 % SDS-PAGE gel. Gels were stained with Coomassie brilliant blue.

### Western blot

After separation of protein samples on SDS-PAGE, proteins were transferred to 0.45 µm Immobilon-PVDF membranes (Millipore) pre-activated with methanol in standard Tris-Glycine transfer buffer (pH 8.3) supplemented with 20% methanol for 2 h at 400 mA. Membranes were blocked in 5% skim milk powder in TBS-T for 1 h, followed by incubation with appropriate primary antibodies on an orbital shaker at 4 °C overnight. Membranes were rinsed thoroughly in TBS-T, followed by incubation with appropriate secondary HRP-conjugated antibodies for 1 h on an orbital shaker at room temperature. Membranes were rinsed thoroughly and developed using WesternBright ECL (Advansta) in a MyECL Imager v 2.2.0.1250 (Thermo Scientific) according to the manufacturer’s instructions.

Primary antibodies used for Western blots include anti-HA(R) rabbit (1:6000, ab9110, Abcam), anti-HA(M) mouse (1:1000, ab18181, Abcam), anti-PSMD1 (1:1000, ab2941, Abcam), anti-PSMD2 (1:1000, PAB6715, Abnova), anti-PSMA3 (1:500, sc-58417, Santa Cruz), anti-PSMA1 (1:1000, ab140499, Abcam), anti-FLAG (1:2500, F3165 Clone M2, Sigma), anti-GFP (1:2500, ab290, Abcam), anti-p53 HRP (1:2500, HAF1355, Biotest), anti-α-synuclein (1:500, ab51252, Abcam), anti-GAPDH (1:1000, MAB374, Clone 6C5, Millipore), anti-CBR3 (1:1000, sc-374393, Santa Cruz).

### Peptide array

The peptide-array screening experiments were performed as explained in ref. ^29^. In brief, CelluSpots^TM^ peptide micro-arrays were synthesized by INTAVIS Bioanalytical Instruments AG, Köln, Germany. In general, peptides are 10–15 residues in length and 16–25 residues when a secondary structure like an entire α-helix or a β-strand is considered. Following structures from PDB were used to design peptides: 1D4A (NQO1), 1UCF (Human DJ-1), 2HRB (CBR3), and the structure of the Hsp33 (3MII) was considered for Hsp32 due to similar sequence between the homologs, and 1PMA (*T. acidophilum* 20S). The web interface of the Protein Homology/analogy Recognition Engine Phyre2 portal was used for generating the structure of archaeal DJ-1 (TA-DJ-1) with amino acid sequence from UniProt (Q9HKX8). In the absence of sequence coverage at the N- or C-terminus of proteins in the PDB structures, their corresponding sequences from the UniProtKB database were used. Further, the peptide sequence for control peptides was derived from the UniProtKB database with the following unique identities: Q58576 (PAN ATPase) in spot H22 (Supplementary Fig. 2F) and spot d12 (Supplementary Fig. 14E), P33297 (*S. cerevisiae* Rpt5) in spot H21, Q63569 (Rpt5) and P62193 (Rpt2) from *R. norvegicus* in spot H23 and H24 respectively (Supplementary Fig. 2F).

The peptides are attached in duplicates to the cellulose membrane by C-termini, and their N-termini are acetylated. To perform the experiment for mapping the proteasome binding sites in CCRs and its reciprocal for mapping the CCRs binding site in archaeal 20S proteasome, the array was first incubated for 4 h at room temperature with 20 mM Tris-HCl pH 7.6, 150 mM NaCl, 0.05% Tween 20 (TBS-T), and 5% (W/V) skimmed milk in order to block unspecific binding. 20S proteasomes or CCRs were diluted in the above-mentioned blocking buffer. Then, 300 μl of 1–2 μM of the proteasomes or 5 μM of CCRs were mixed with blocking buffer and incubated under coverslips with the sealing system from HybriWell^TM^ Hybridization System (GRACE Bio-Labs) on the arrays at 4 °C with shaking overnight. After three washes with TBS-T, the array was incubated with anti-His-HRP (1:1500, sc-8036, Santa Cruz Biotechnology), anti-His antibody (1:2500, A00174, Genscript), anti-NQO1 (1:2500, Ab34173, Abcam), anti-CBR3 (1:2500, 15619-1-AP, Proteintech), and anti-FLAG (1:2500, F3165 Clone MS, Sigma) antibody at room temperature for 1 h. The array was washed again three times with TBS-T. Immunodetection was performed using chemiluminescence WesternBright ECL (Advansta) in a MyECL Imager (Thermo Scientific). Peptide spot intensities from three independent experiments were measured using ImageJ 1.51k, R v.4 for determining 20S proteasomes binding sites in CCRs, and all values were ranked from the largest to the smallest, where only the top 15% of all the peptides are displayed in bar graphs. For experiments to determine CCRs CBR3, NQO1, and C-PLoop binding sites in archaeal 20S proteasome and 20S proteasomes binding sites in C-PLoop, a similar experimental procedure as explained above was followed, and peptide intensities from four independent experiments were measured and top 15 up to the top 17% of the peptides are presented in bar graphs.

### NanoDSF measurements

Thermal unfolding experiments for wild-type CBR3, NRas, DJ-1, and their respective mutants were carried out by nanoDSF Prometheus NT.48 (NanoTemper Technologies GMBH). Proteins at 1 mg/ml were filled into capillary tubes in duplicates by capillary force action, and the tubes were heated from 20 to 90 °C with a heating rate of 1 °C/min and the changes in the fluorescence ratio were detected at 330 and 350 nm to derive 350/330 ratio to determine the apparent T_m_. The nanoDSF data analysis was performed using PR.ThermControl v2.1.1 (NanoTemper Technologies GMBH).

### Chimera

UCSF Chimera 1.16^97^ was used to measure the distances between the PSMB4 and PSMB6 (caspase-like), PSMB5 (chymotrypsin-like), PSMB7(trypsin-like) active subunits of the rat 20S proteasome (PDB: 6TU3). Coulombic surface potential was measured for the PSMB4 subunit from the rat 20S proteasome (PDB: 6TU3) and human CBR3 (PDB: 2HRB) using Chimera.

### Statistics and reproducibility

All experiments were performed three times unless specified otherwise. The statistical details of each assay and the number of experiments are given in the relevant figure details, along with the performed statistical test. Data were analyzed with Microsoft Excel 2016 and GraphPad Prism V6 software using one-tailed or two-tailed Student’s *t*-tests and one-way ANOVA (*p ≤ 0.05, **p ≤ 0.01, ***p ≤ 0.001, ****p ≤ 0.0001). All data are presented as mean ± standard deviation (SD) or as ±standard error (SEM) as specified.

### Reporting summary

Further information on research design is available in the Nature Portfolio Reporting Summary linked to this article.

## Supplementary information

Supplementary Information

Description of Additional Supplementary Files

Supplementary Dataset 1

Supplementary Dataset 2

Supplementary Dataset 3

Reporting Summary

## Data Availability

The datasets generated and all constructs (wild-type and mutants) used in the current study are available from the corresponding author on request. Cryo-EM map determined from the 20S proteasome and CBR3 complex dataset has been deposited at the Electron Microscopy Data Bank with accession codes EMD-17118. The human constitutive 20S proteasome structure 4R3O was used as a reference for initial refinement, and rat 20S proteasome structure 6TU3 was used to superimpose the electron density map of the 20S-CBR3 complex. A Chimera session presenting the 20S-CBR3 map with corresponding angular distribution is provided as Supplementary Data 3. Source data (uncropped images, blots, and data points underlying graphs) are provided with this paper.

## Source data

Source Data
