# Supplementary material for: Allosteric regulation of the 20S proteasome by the Catalytic Core Regulators (CCRs) family
Source: Nat Commun. 2023 May 30;14:3126. doi: 10.1038/s41467-023-38404-w (PMC10229617; doi:10.1038/s41467-023-38404-w)
Supplement: Supplementary file 1 — Supplementary Information [file 41467_2023_38404_MOESM1_ESM.pdf]

# Supplementary information

# Allosteric regulation of the 20S proteasome by the Catalytic Core Regulators (CCRs) family

Fanindra Kumar Deshmukh[1], Gili Ben-Nissan[1], Maya A Olshina[1], Maria G. Füzesi-Levi[1], Caley Polkinghorn[1], Galina Arkind[1], Yegor Leushkin[1], Irit Fainer[1], Sarel J Fleishman[1], Dan Tawfik[1] and Michal Sharon[1*]

[1]Department of Biomolecular Sciences, Weizmann Institute of Science, Rehovot 7610001, Israel.

[*]Correspondence:

Michal Sharon

michal.sharon@weizmann.ac.il

Tel: +972-8-934-3947

# Supplementary Figures

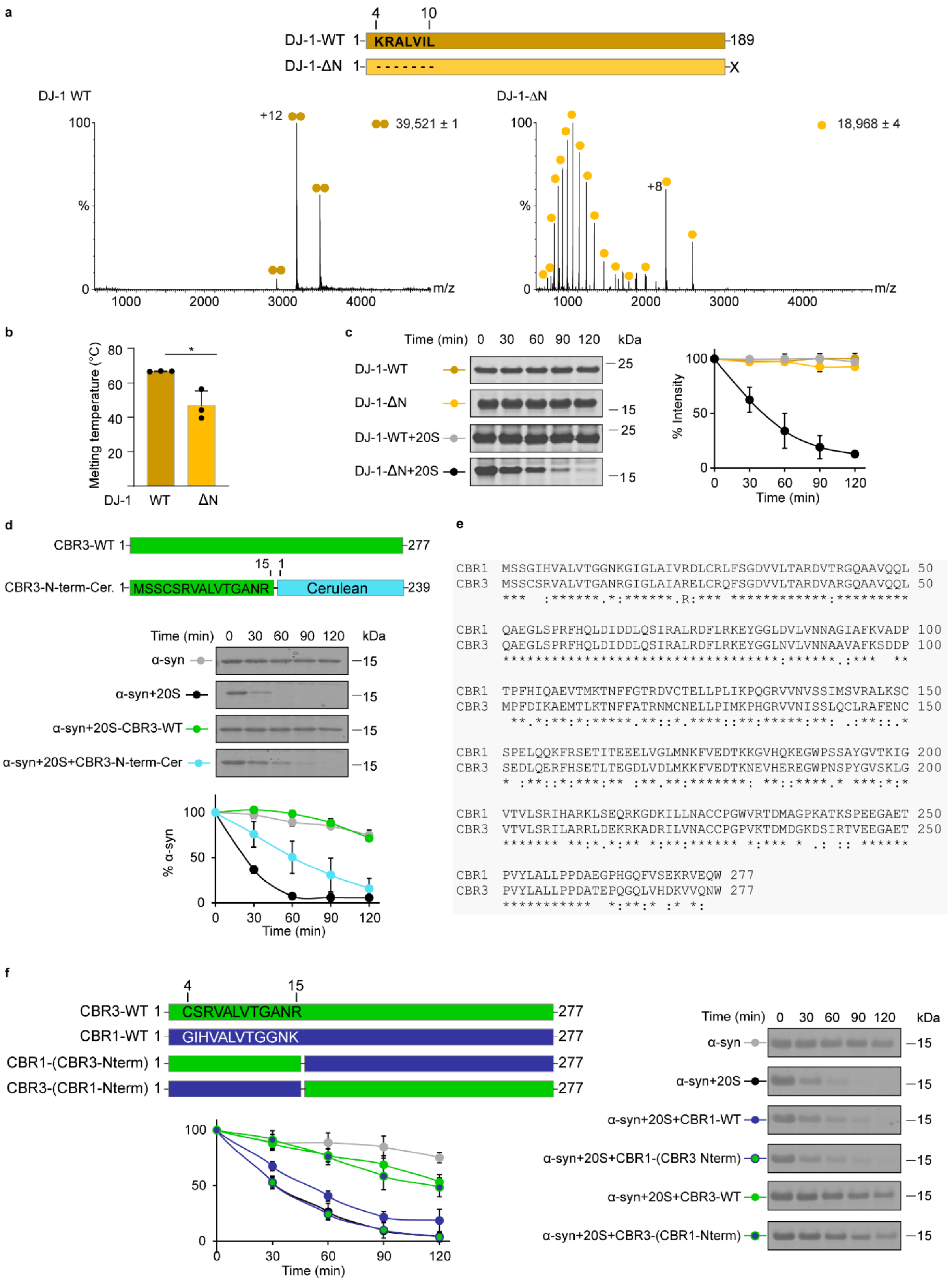

**Supplementary Figure 1. The conserved N-terminus sequence motif is not sufficient for CCRs activity.** (**a**) MS spectra of DJ-$1_{WT}$ (left) and DJ-$1_{\Delta N}$ (right). The removal of the N-terminal region converts the dimeric DJ-1 into a monomer and give rise to a wide distribution of charge states, which indicates the coexistence of the monomeric protein and partially unfolded conformers. The schematic representation of DJ-$1_{WT}$ and the N-terminal deleted region in DJ-$1_{\Delta N}$ is shown on the top. (**b**) Melting temperature (Tm) measured by NanoDSF experiments reveal that the thermal stability of DJ-$1_{\Delta N}$ is lower than that of the WT protein. Bars represent mean values from three independent experiments, error bars represent SD. Measurements were subjected to one-tailed Student *t*-test analysis, * represents *p* value=0.0297. (**c**) Time dependent *in vitro* degradation assays. DJ-$1_{\Delta N}$ was degraded by the 20S proteasome, confirming that it adopts a partially unfolded conformation. (**d**) Schematic representation of CBR3 N-terminal sequence fused to Cerulean (Cer) to generate $CBR3_{N\text{-}term}$-Cer. Time dependent *in vitro* degradation assays revealed that unlike CBR3, the N-terminus CBR3 fragment fused to Cerulean only partially protected $\alpha$-synuclein ($\alpha$-syn) from 20S proteasome mediated degradation (**d**-lower panel). (**e**) Clustal Omega sequence alignment of the paralog CBR3 and CBR1 proteins. (**f**) Schematic representation of the CBR3 and CBR1 generated constructs. Time dependent *in vitro* degradation assays of the CBR3 and CBR1 constructs (**f**). Switching between the N-terminal region of CBR3 and CBR1 only slightly deactivated CBR3 and vice versa for CBR1 as presented in the degradation assays. All bar and scatter plot represent average of three independent experiments. Error bars represent SD. Source data are provided with this paper.

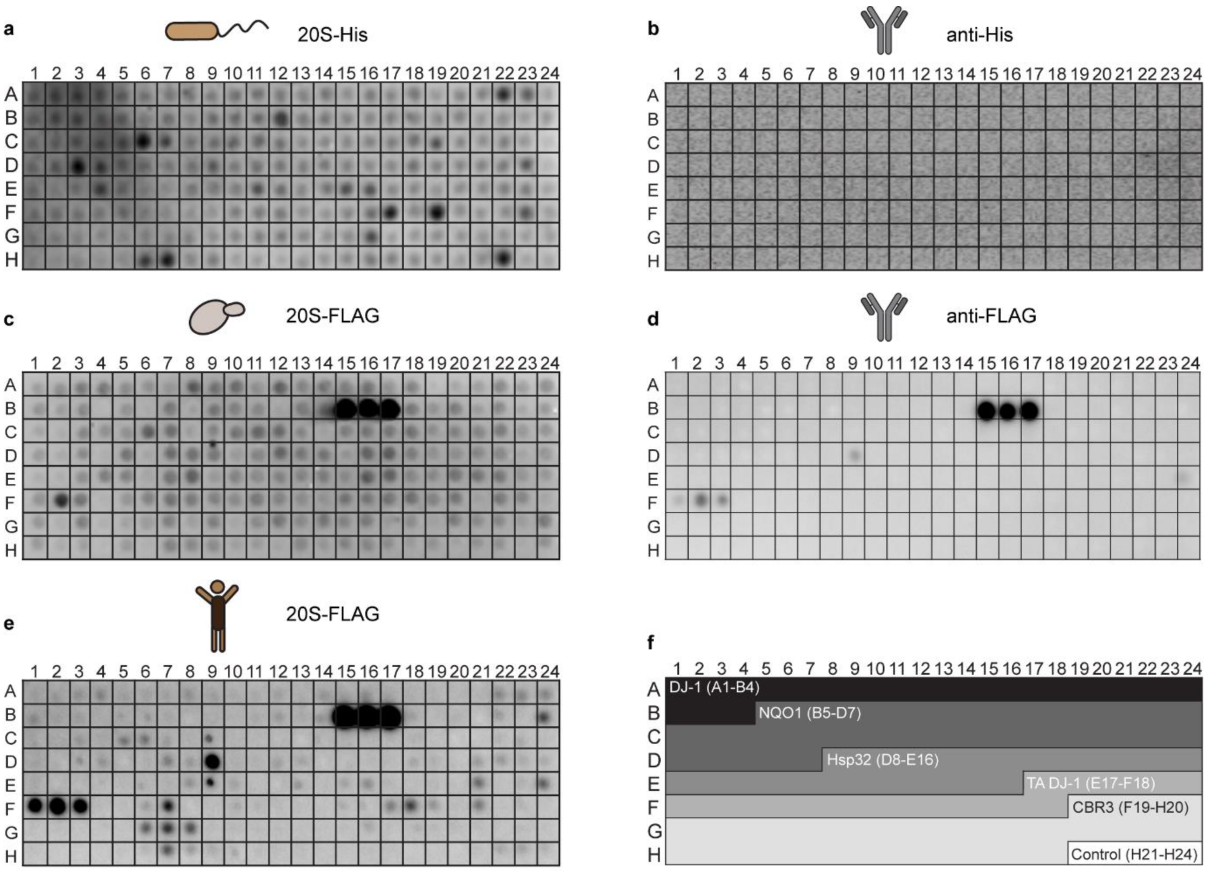


g

| Peptide spot | Protein | Residues | Amino acid sequence |
|---|---|---|---|
| A1 | DJ-1 | 1-15 | MASKRALVILAKGAE |
| A3 | DJ-1 | 15-29 | EEMETVIPVDVMRRA |
| A4 | DJ-1 | 22-36 | PVDVMRRAGIKVTVA |
| A14 | DJ-1 | 85-99 | SAAVKEILKEQENRK |
| A22 | DJ-1 | 141-156 | YSENRVEKDGLILTSR |
| A23 | DJ-1 | 148-162 | KDGLILTSRGPGTSF |
| A24 | DJ-1 | 155-169 | SRGPGTSFEFALAIV |
| B1 | DJ-1 | 162-176 | FEFALAIVEALNGKE |
| C5 | NQO1 | 127-141 | YTYAAMYDKGPFRSK |
| C6 | NQO1 | 134-148 | DKGPFRSKKAVLSIT |
| C7 | NQO1 | 137-151 | PFRSKKAVLSITTGG |
| D3 | NQO1 | 246-260 | EEKNKKFGLSVGHHL |
| D4 | NQO1 | 248-262 | KNKKFGLSVGHHLGK |

| Peptide spot | Protein | Residues | Amino acid sequence |
|---|---|---|---|
| D7 | NQO1 | 260-274 | LGKSIPTDNQIKARK |
| D12 | Hsp32 | 29-43 | VEILRSFDTFEKHGF |
| D13 | Hsp32 | 36-50 | DTFEKHGFEVDFVSE |
| E7 | Hsp32 | 162-176 | ITGFPLEGEIALGVD |
| E8 | Hsp32 | 169-183 | GEIALGVDDILRSRK |
| E20 | TA DJ-1 | 22-36 | QRMKEEGFEVDVAAP |
| E21 | TA DJ-1 | 29-43 | FEVDVAAPEKKKIQL |
| E22 | TA DJ-1 | 36-50 | PEKKKIQLVVHDFEE |
| F17 | TA DJ-1 | 169-183 | HPEWMRAFIKLLKEK |
| F18 | TA DJ-1 | 172-186 | WMRAFIKLLKEKVKK |
| F19 | CBR3 | 1-15 | MSSCSRVALVTGANR |
| H6 | CBR3 | 192-206 | SPYGVSKLGVTVLSR |
| H7 | CBR3 | 197-211 | SKLGVTVLSRILARR |
| H8 | CBR3 | 206-220 | RILARRLDEKRKADR |

**Supplementary Figure 2. Mapping of CCR binding sites to the 20S proteasome.** The peptide array was screened for 20S proteasome binding to CCR derived peptides. Representative peptide array results demonstrating binding to 20S proteasome isolated from (**a**) archaea, (**c**) yeast and (**e**) human. To detect unspecific binding each array was incubated with the secondary antibody (**b**) anti-His and (**d**) anti-FLAG without prior incubation of 20S proteasomes. (**f**) Peptide array design of the different CCRs and control peptides. Three independent experiments in duplicates were performed and top 15% peptide spots that bound to at least two out of three proteasomes species are presented in the table (**g**).

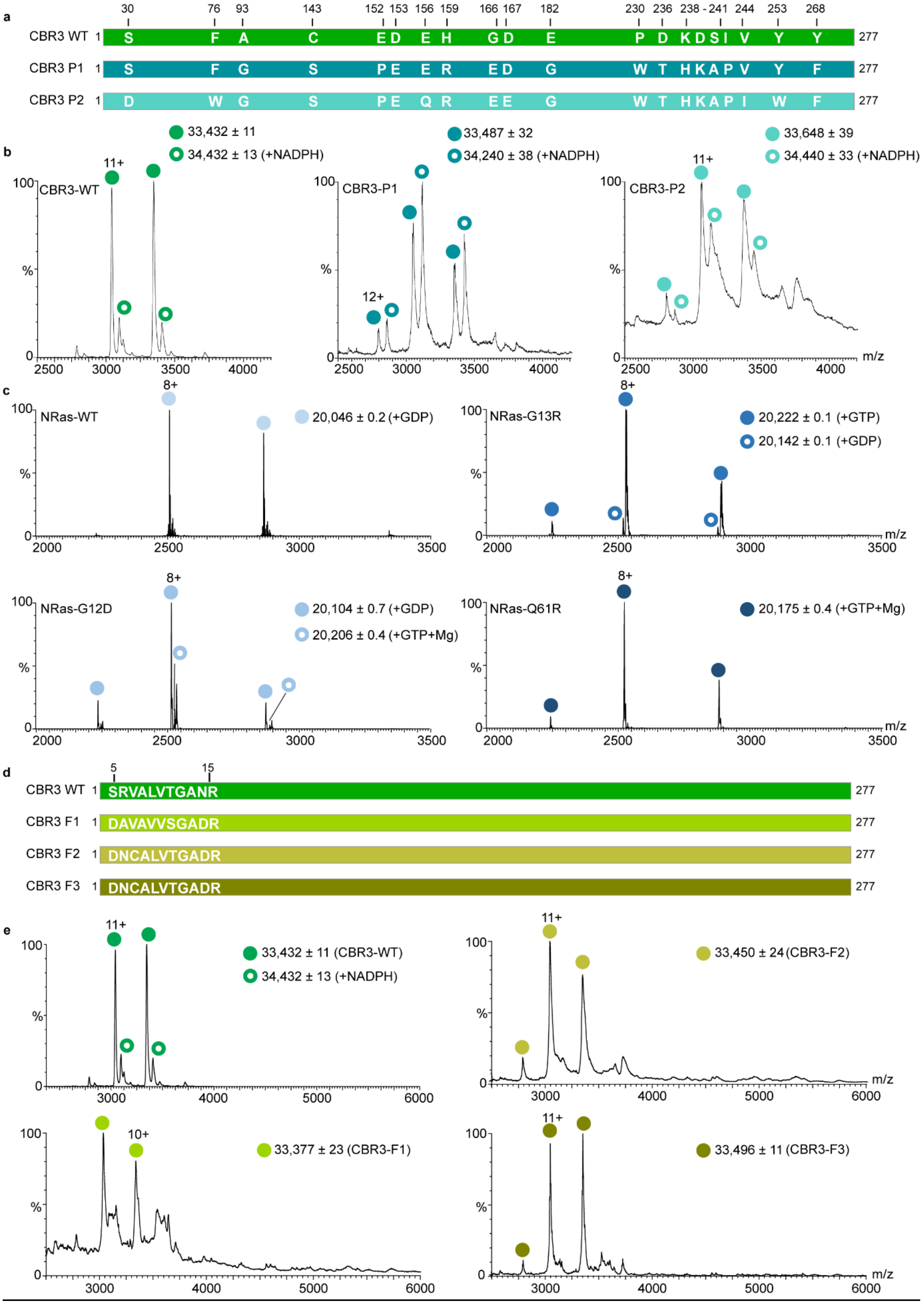

a
CBR3 WT
CBR3 P1
CBR3 P2
30 76 93 143 152 153 156 159 166 167 182 230 236 238 - 241 244 253 268
S F A C E D E H G D E P D K D S I V Y Y
S F G S P E E R E D G W T H K A P V Y F
D W G S P E Q R E E G W T H K A P I W F
277
b
33,432 ± 11
34,432 ± 13 (+NADPH)
33,487 ± 32
34,240 ± 38 (+NADPH)
33,648 ± 39
34,440 ± 33 (+NADPH)
CBR3-WT
CBR3-P1
CBR3-P2
11+
12+
m/z
c
NRas-WT
20,046 ± 0.2 (+GDP)
NRas-G13R
20,222 ± 0.1 (+GTP)
20,142 ± 0.1 (+GDP)
NRas-G12D
20,104 ± 0.7 (+GDP)
20,206 ± 0.4 (+GTP+Mg)
NRas-Q61R
20,175 ± 0.4 (+GTP+Mg)
8+
d
CBR3 WT
CBR3 F1
CBR3 F2
CBR3 F3
5 15
SRVALVTGANR
DAVAVVSGADR
DNCALVTGADR
DNCALVTGADR
277
e
33,432 ± 11 (CBR3-WT)
34,432 ± 13 (+NADPH)
33,450 ± 24 (CBR3-F2)
33,377 ± 23 (CBR3-F1)
33,496 ± 11 (CBR3-F3)
11+
10+
m/z

**Supplementary Figure 3. Structural characterization of CBR3 and NRas mutants.** (**a**) Schematic representation of WT CBR3 and its P1 and P2 variants generated using the PROSS algorithm. (**b**) Native MS analysis directly from crude cell lysates of recombinant CBR3 WT and P variants. The CBR3 WT and P variants are detected in their apo- and NADPH bound forms (white spotted). (**c**) Native MS analysis of purified NRas and its missense mutants, G12D, G13R and Q61R. The mutants display a similar charge state distribution as the WT protein. The GTP and GDP bound forms are labeled. (**d**) Schematic representation of WT CBR3 and its mutational variants, F1-F3, designed within the internal β-strand ($^{5}$SRVALVTGANR$^{15}$) using the FuncLib algorithm. (**e**) Native MS characterization of recombinant CBR3 WT and its F1, F2 and F3 variants analyzed directly from crude cell lysates. The charge state distribution of CBR3 F1-F3 variants indicated that they are folded, however, they lost their ability to bind the NADPH cofactor.

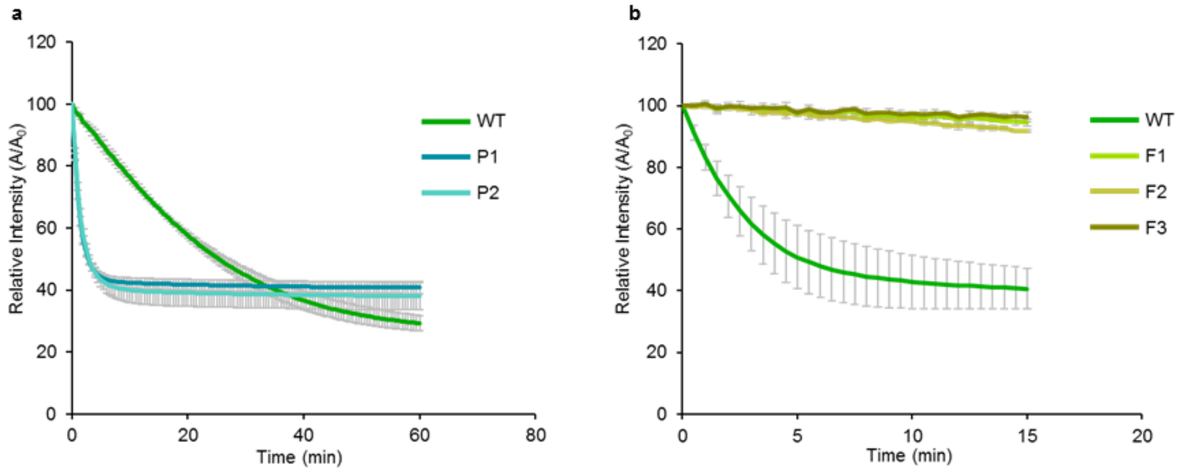


**Supplementary Figure 4. Enzymatic activity of the CBR3 PROSS and FuncLib mutants.** NADPH absorbance was measured as enzyme activity for CBR3 PROSS (**a**) and FuncLib (**b**) mutants using Isatin as the substrate. Scatter plots represent mean values of three independent experiments. Error bars represent SD. Source data are provided with this paper.

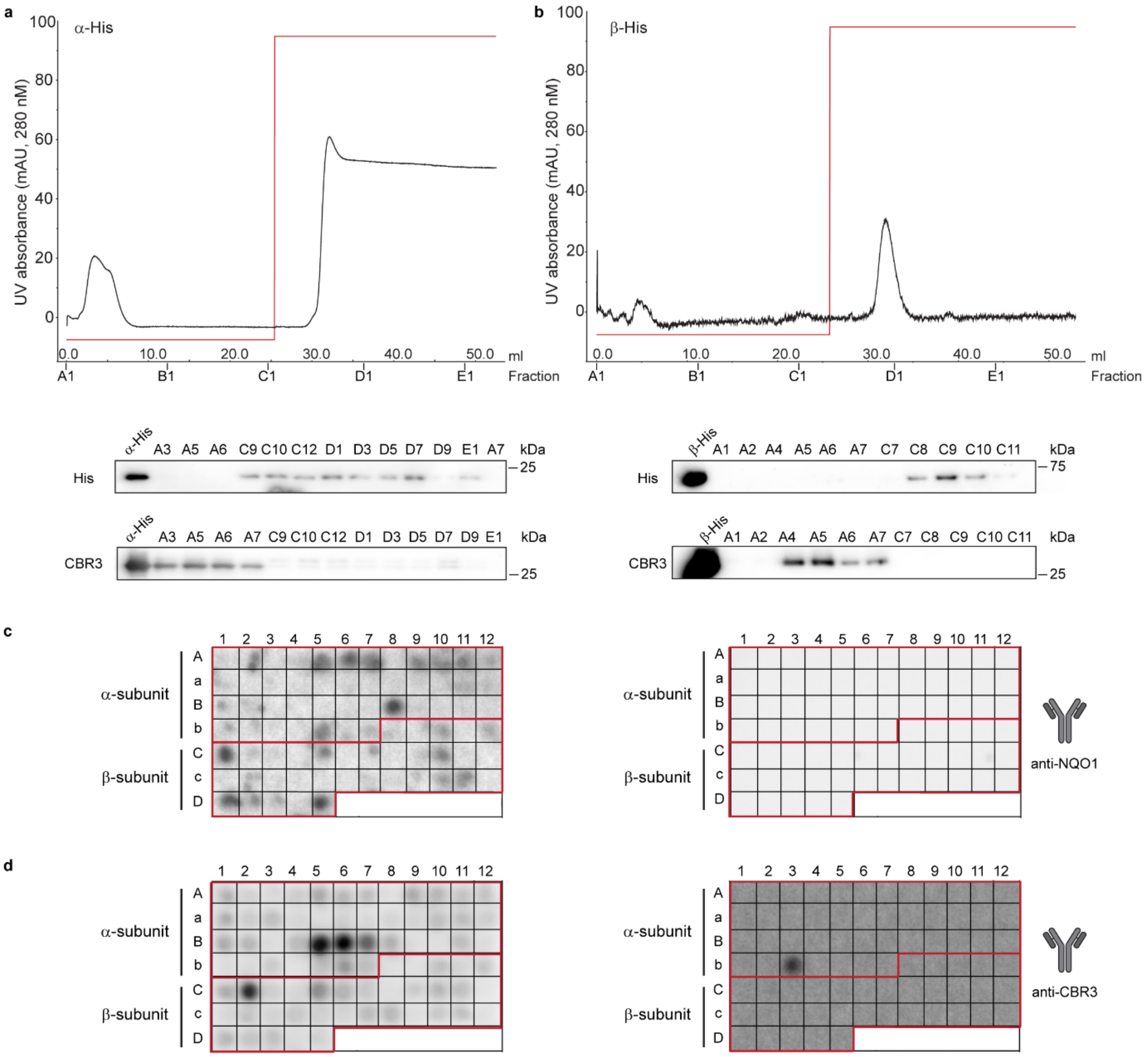


e

| CCRs | Spot | Protein | Residues | Amino acid sequence |
|---|---|---|---|---|
| CBR3 | A6 | α-subunit | 29-43 | EAVKKGSTALGMKFA |
| NQO1 | A5 | α-subunit | 19-34 | GRLFQVEYAREAVKKG |
| NQO1 | A6 | α-subunit | 29-43 | EAVKKGSTALGMKFA |
| NQO1 | A7 | α-subunit | 33-45 | KGSTALGMKFANG |
| CBR3 | B-6 | α-subunit | 144-160 | IGPRLFDCDPAGTINEY |
| CBR3 | B-7 | α-subunit | 148-162 | LFDCDPAGTINEYKA |
| NQO1 | B-8 | α-subunit | 155-169 | GTINEYKATAIGSGK |
| NQO1 | b-5 | α-subunit | 206-220 | ELKAPEIASITVGNK |
| CBR3 | b-6 | α-subunit | 211-225 | EIASITVGNKYRIYD |
| NQO1 | C-1 | β-subunit | 29-43 | TMENFIMHKNGKKLF |
| CBR3 | C-2 | β-subunit | 36-50 | HKNGKKLFQIDTYTG |

**Supplementary Figure 5. Determining the CCR binding sites to the 20S proteasome.** CBR3 was incubated with His-tagged purified (**a**) $\alpha$- and (**b**) $\beta$-subunits of the archaeal 20S proteasome and loaded onto a Ni-NTA column. Eluted fractions were subjected to immunoblot analysis with anti-His and anti-CBR3 antibodies. CBR3 eluted in the flow-through, suggesting that it does not interact with the $\alpha$7 ring and non-assembled $\beta$-subunits. Representative peptide array results demonstrating binding of (**c**) NQO1 and (**d**) CBR3 to the archaeal 20S proteasome. Control experiments were performed with anti-NQO1 and anti-CBR3 antibodies without prior incubation of the respective CCRs (**c** and **d** right panels). (**e**) Peptide spots within top 15% intensity that bound to overlapping sequence (underlined) in both CCRs are presented in the table. Source data are provided with this paper.

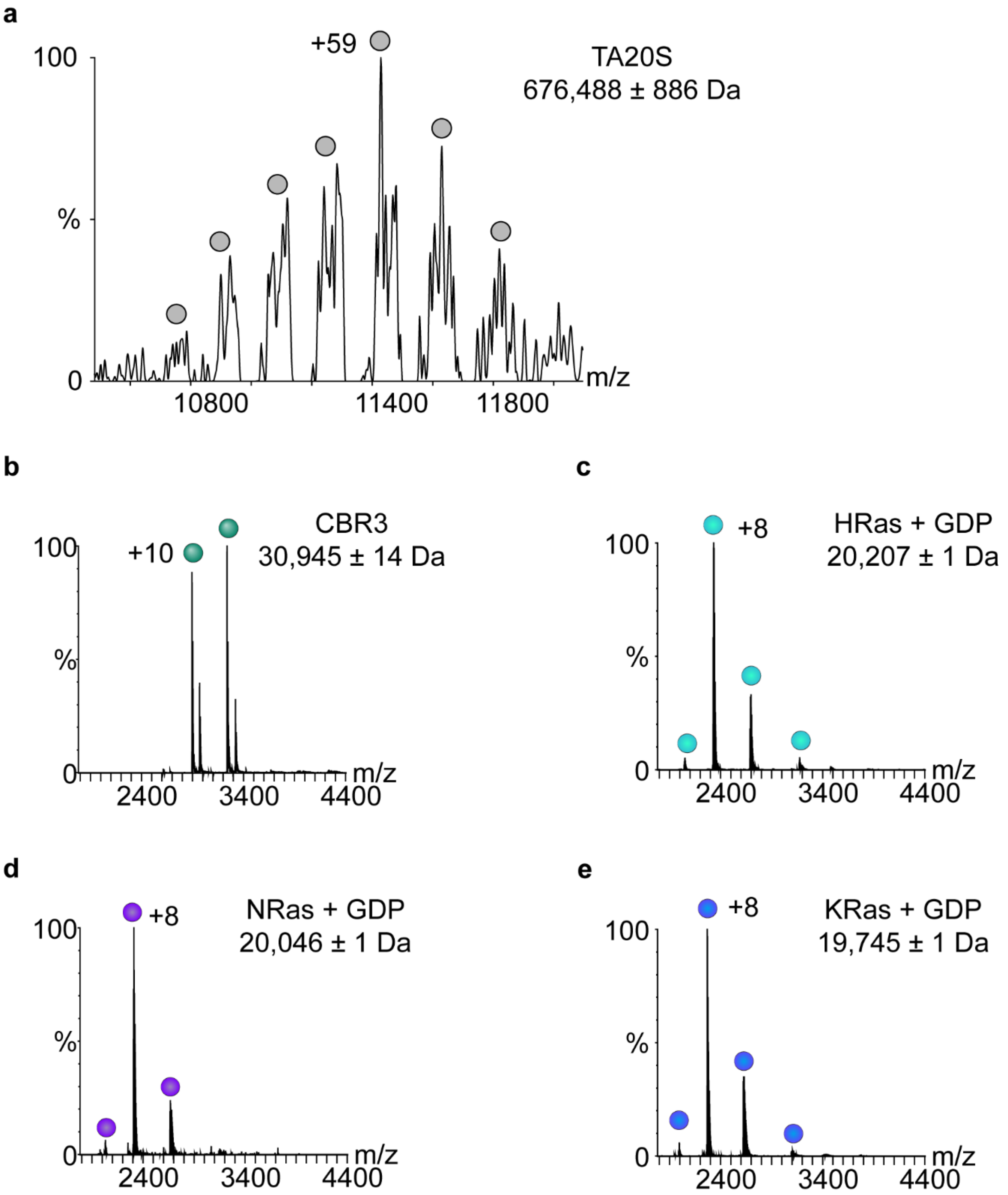


**Supplementary Figure 6. Native MS spectra of the intact *T. acidophilum* 20S proteasome and the free CCRs.** (**a**) A representative spectrum of the free *T. acidophilum* 20S proteasome. Mass measurement was performed using the UniDec algorithm. Standard deviations were calculated from three independent measurements. (**b-e**) Expansion of the low m/z region of the spectra shown in Figure 5 **g-j**, reveals the unbound CCR populations of CBR3, HRas, NRas and KRas.

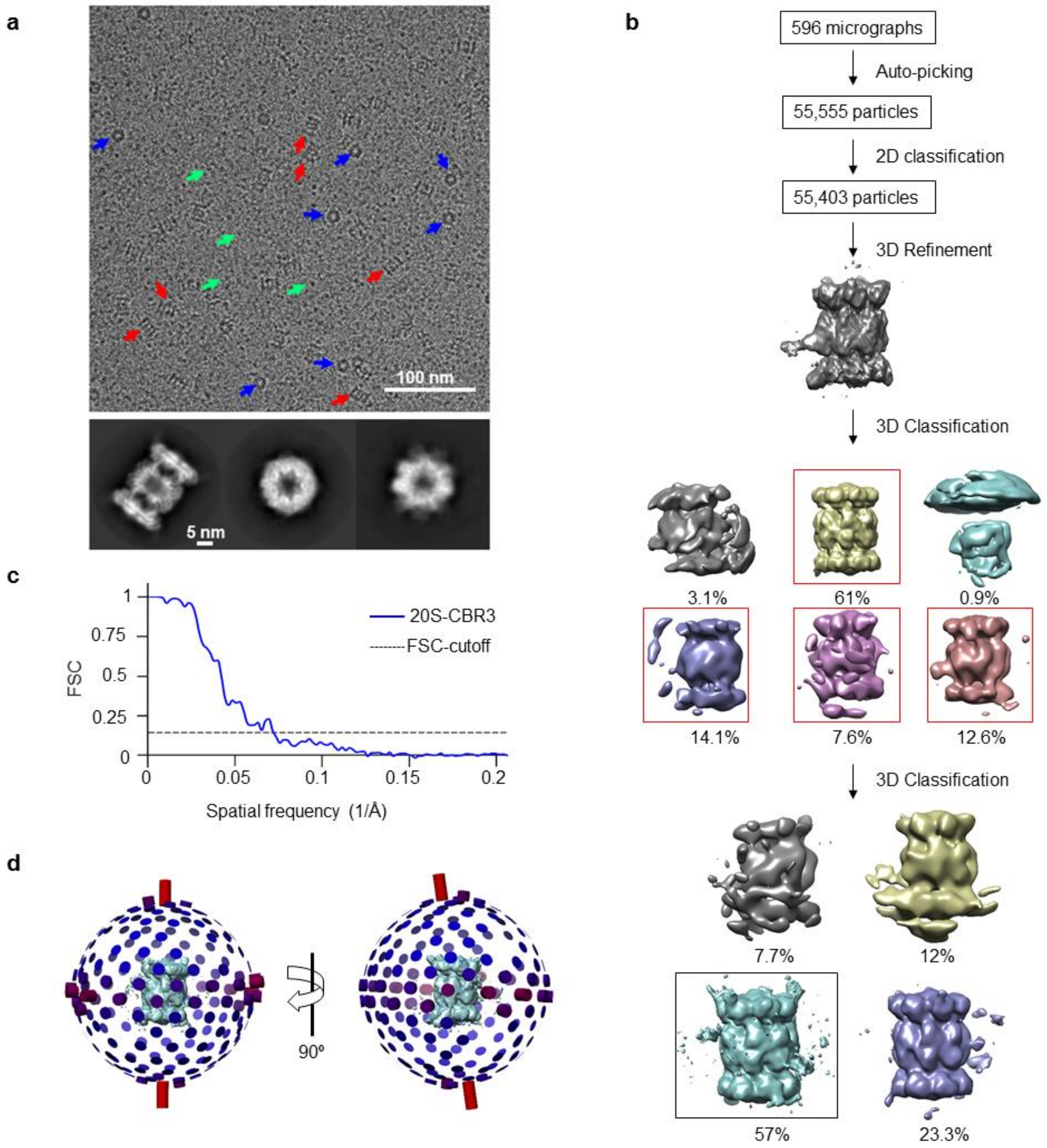


**Supplementary Figure 7: Cryo-EM data processing workflow for the rat 20S proteasome and human CBR3. (a)** A representative cryo-EM micrograph of the 20S proteasome CBR3 mixture. The red and blue arrows represent side and top views of the 20S proteasome, respectively. Green arrows point to CBR3. Selected reference-free 2D class averages are shown on the bottom. (**b**) Single-particle cryo-EM data-processing scheme. Selected 3D classes for further refinement are highlighted in red boxes. 3D class highlighted in the black box displayed extra densities for CBR3 and it is presented with a soft mask in Fig.7a (**c**) Fourier Shell Correlation (FSC) plot for resolution estimation of the 20S proteasome-CBR3 map according to gold standard FSC criterion of 0.143 cut-off (dashed-line). (**d**) Angular distribution plot of the 20S proteasome-CBR3 cryo-EM map that is highlighted with a black box in panel (**b)**.

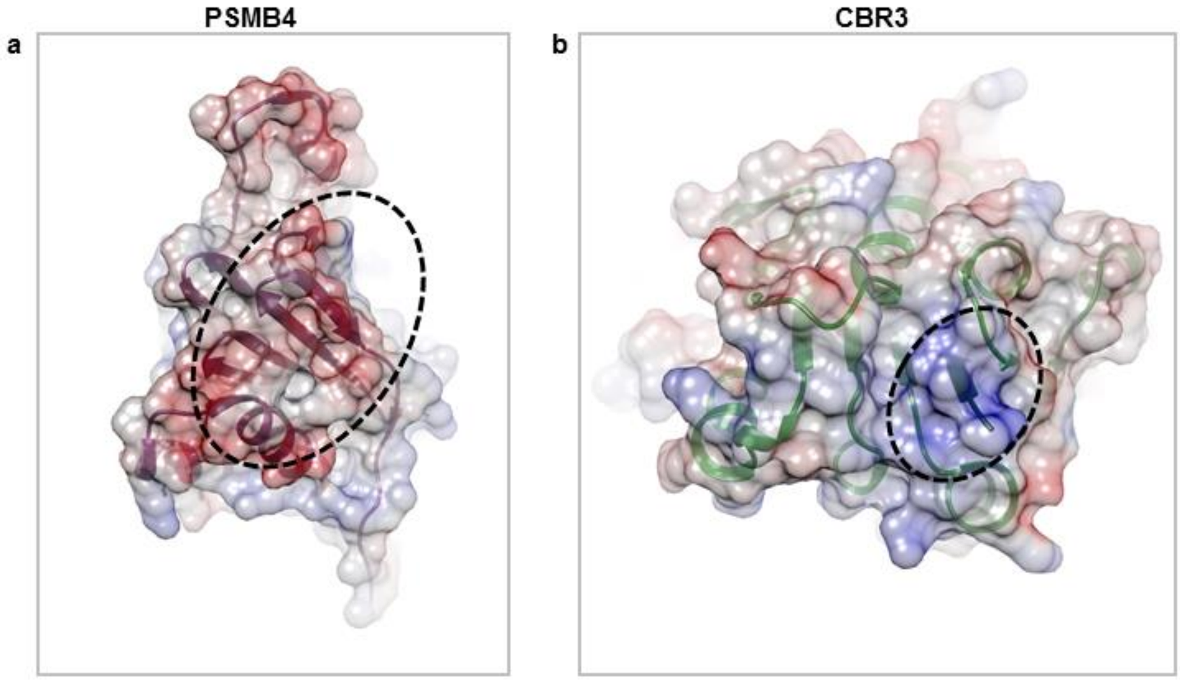


**Supplementary Figure 8. Electrostatic potentials of the PSMB4 and CBR3 interacting regions.** Coulombic surface potentials are displayed and colored by charge (red negative, blue positive) for the **(a)** PSMB4 subunit of the 20S proteasome and **(b)** CBR3. Black dashed circles indicate the region involved in the interaction.

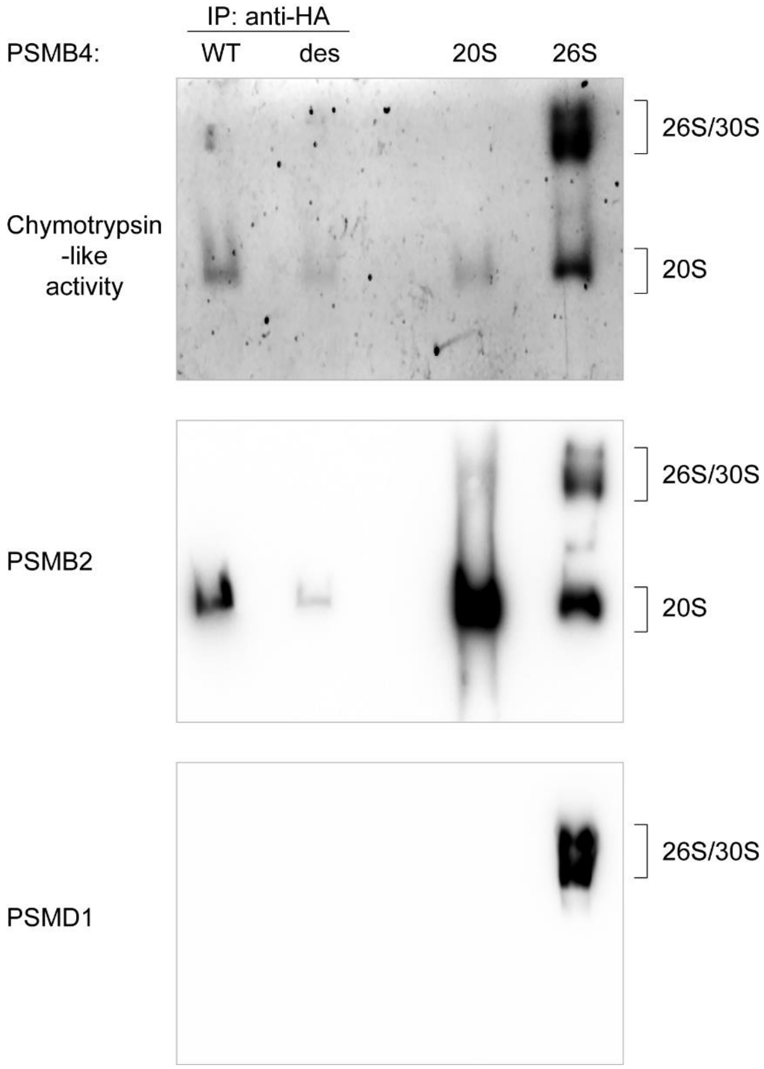


**Supplementary Figure 9. Assembled and active 20S proteasomes are purified by HA-affinity purification from cells overexpressing PSMB4-WT and PSMB4-des.** Proteasome complexes were HA-affinity purified from HEK293T cells, overexpressing either PSMB4-HA WT or des. To verify their composition, the purified complexes were separated on native gels, and probed for the chymotrypsin like catalytic activity and antibodies recognizing PSMB2 (20S subunit) and PSMD1 (19S subunit). Analyses show that only 20S proteasomes were purified. Purified 20S and 26S proteasomes were used as a control (right side of the gels). Source data are provided with this paper.

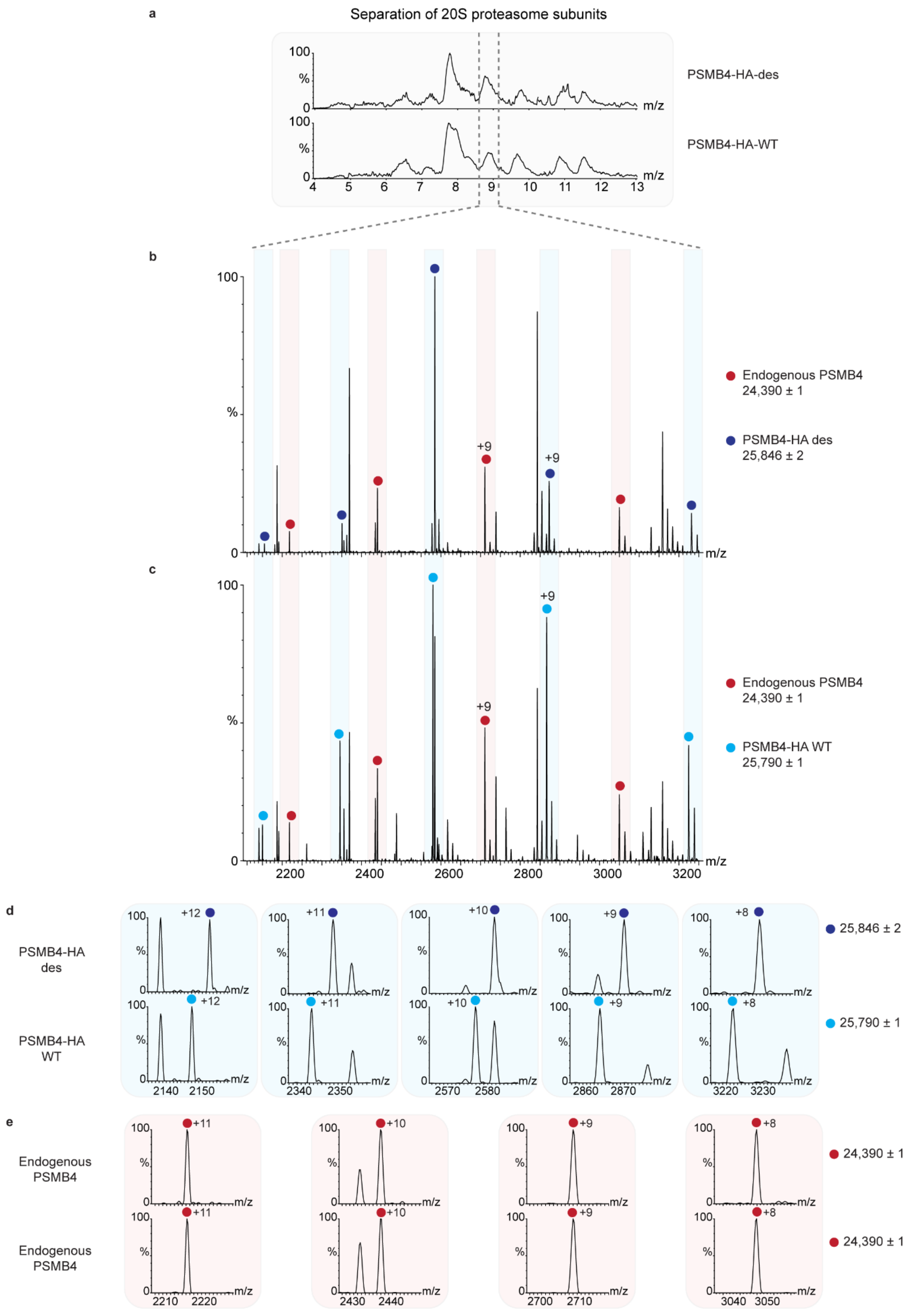

a
Separation of 20S proteasome subunits
PSMB4-HA-des
PSMB4-HA-WT
100
%
0
m/z
4 5 6 7 8 9 10 11 12 13
b
Endogenous PSMB4
24,390 ± 1
PSMB4-HA des
25,846 ± 2
+9
c
PSMB4-HA WT
25,790 ± 1
2200 2400 2600 2800 3000 3200
d
PSMB4-HA des
PSMB4-HA WT
+12 +11 +10 +9 +8
2140 2150
2340 2350
2570 2580
2860 2870
3220 3230
25,846 ± 2
25,790 ± 1
e
Endogenous PSMB4
+11 +10 +9 +8
2210 2220
2430 2440
2700 2710
3040 3050
24,390 ± 1

**Supplementary Figure 10. HA-affinity purified 20S proteasomes contain both endogenous and over-expressed PSMB4-HA subunits**. 20S proteasomes, purified from HEK293T cells, overexpressing either PSMB4-HA WT or des, were separated into their composing subunits on a reversed phase monolithic column and eluted over a gradient of 29%-41% acetonitrile, and directly sprayed into a mass spectrometer for accurate mass analysis. (**a**) Elution chromatograms of the separated proteasome subunits. PSMB4 subunits were eluted at 8.5-9.2 minutes, at ~35-36% acetonitrile, as marked by the dashed lines. (**b-c**) Spectra of the eluted proteins, showing charge state series of different proteasome subunits. Red circles denote the charge state series of endogenous mature PSMB4. Dark blue and light blue circles denote charge state series of PSMB4-des and PSMB4-WT, which were assembled into the complex. (**d-e**) Extended view of the spectra shown in (**b** and **c**), indicate single charge states of the different PSMB4 variants. The results indicate that HA-affinity purified 20S proteasome complexes contain both recombinant and endogenous PSMB4 subunits.

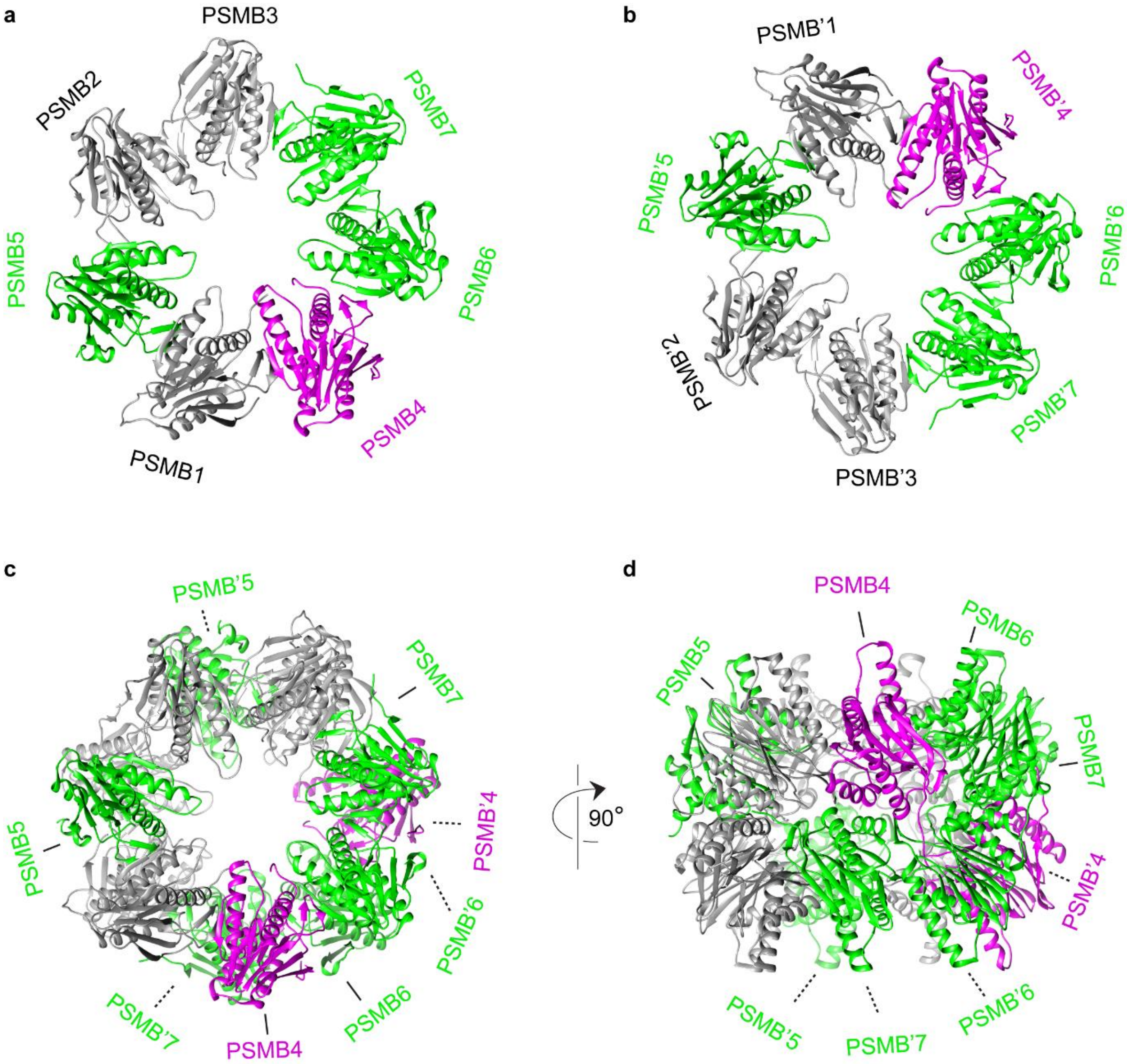


**Supplementary Figure 11. PSMB4 is proximal to the catalytic subunits PSMB6 and PSMB7.** Atomic model of rat 20S proteasome β-ring (PDB:6TU3) displaying the PSMB4 subunit (magenta) and the catalytic active subunits (green) in the cis- and trans-β-rings. (**a-b**) Subunit organization of the β-subunits within the top and bottom β-rings, respectively. (**c-d**) Top and side views of the two assembled β-rings, respectively. Subunits in the trans β-ring are indicated by an apostrophe and dashed line.

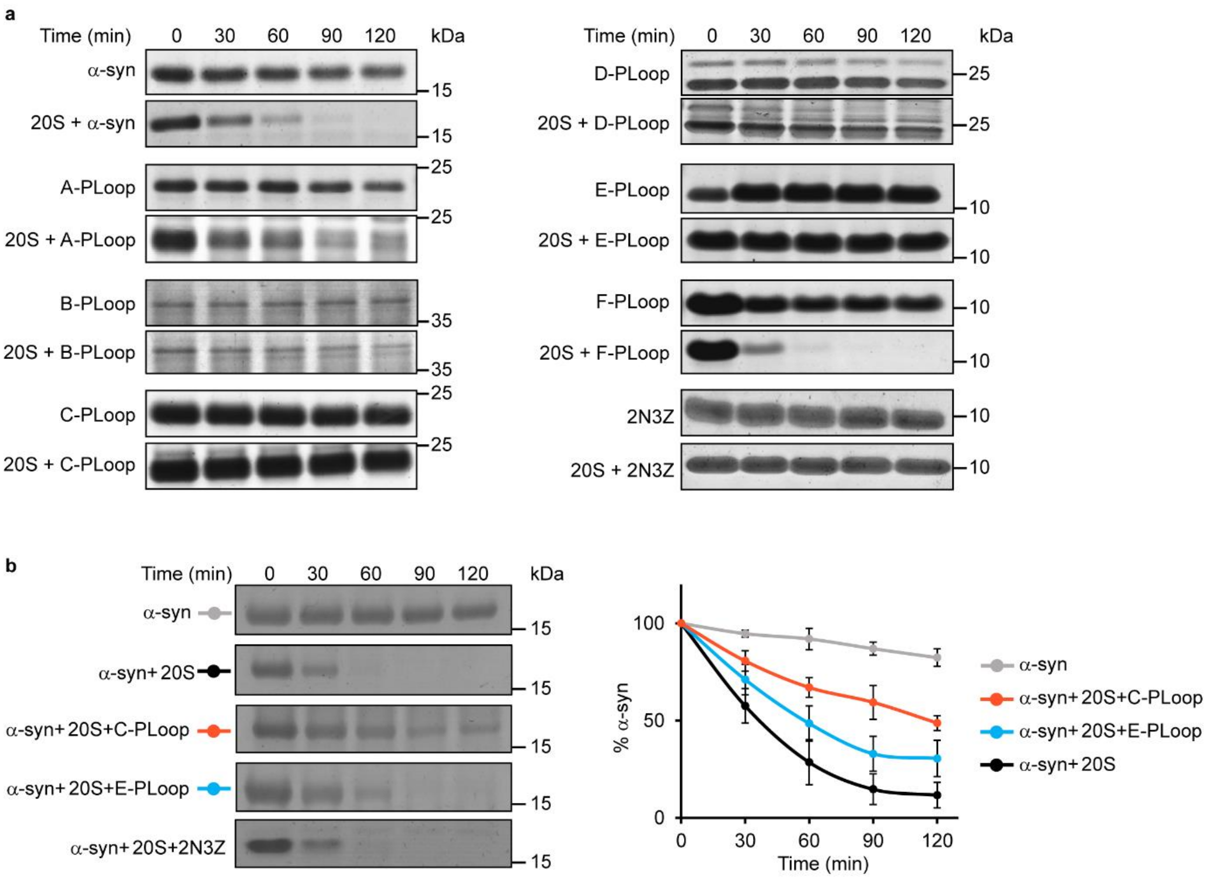


**Supplementary Figure 12. Susceptibility of the P-Loop constructs towards 20S mediated degradation.** Representative time-dependent degradation assays. (**a**) The susceptibility of α-synuclein (α-syn) and P-Loop protein constructs towards 20S proteasome mediated degradation was examined. (**b**) The ability of C-, E-PLoop and 2N3Z to regulate the degradation of α-syn was determined using the rat 20S proteasome. Quantification of three independent experiments is displayed on the right as mean intensities. Error bars represent SD. Source data are provided with this paper.

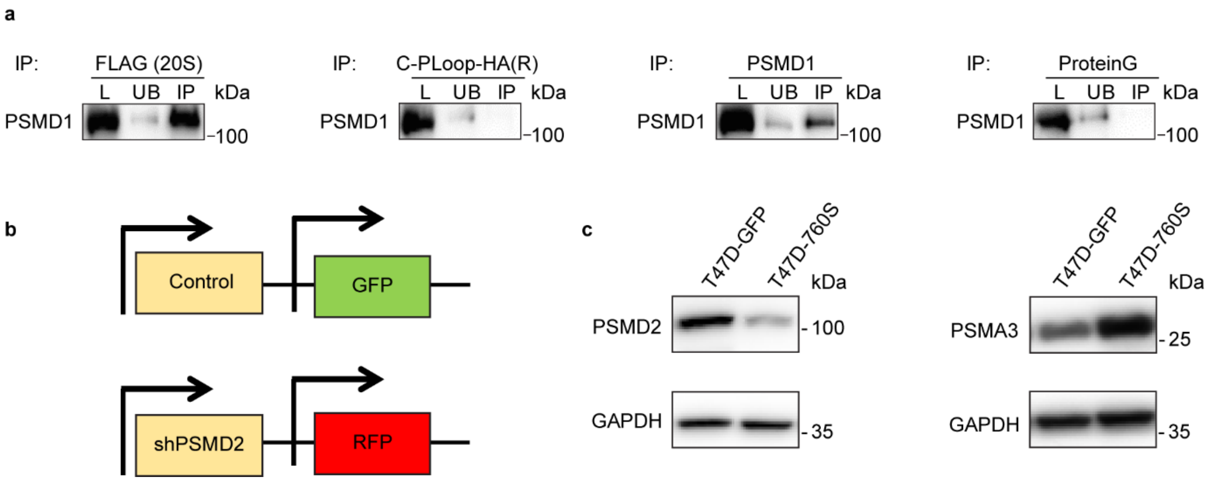


**Supplementary Figure 13. Decoupling the 20S and 26S proteasome activities.** (**a**) A longer exposure of the PSMD1 blot shown in Figure 9d-g indicating a preference for C-PLoop binding to the 20S proteasome. (**b**) Schematic diagram of the doxycycline (dox)-inducible system T47D-760S and the control strain T47D-GFP. (**c**) Protein content in the T47D transformed cells, in the presence or absence of PSMD2 shRNA induction, was analyzed by immunoblot. Upon dox treatment, the PSMD2 level was markedly reduced in parallel to an increase in the 20S proteasome subunit PSMA3 levels. Source data are provided with this paper.

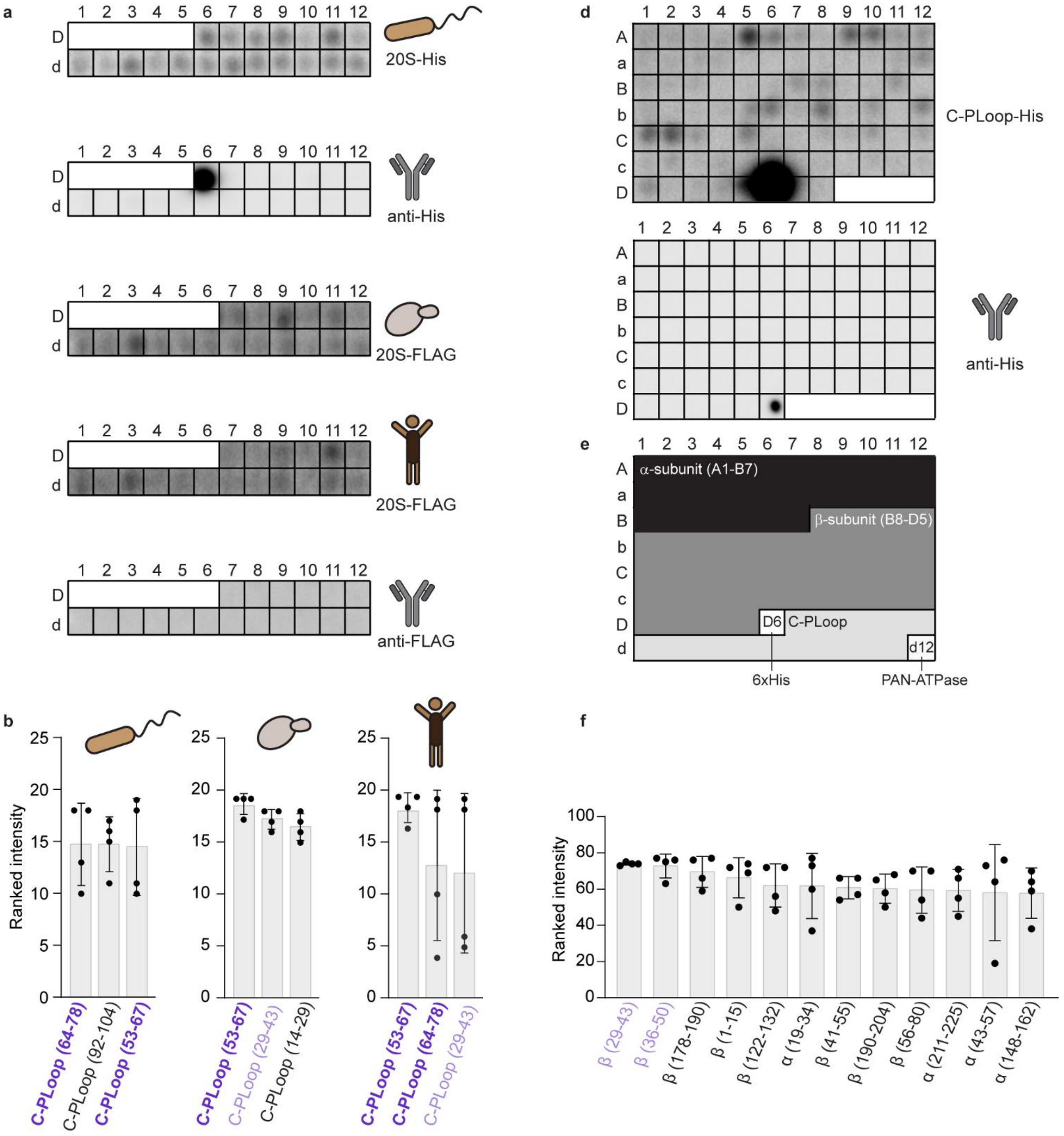


c

| Spot | Residues | Amino acid sequence |
|---|---|---|
| D-11 | 29-43 | VPDAEIRTVTTKEDA |
| d-3 | 53-67 | RNADIVVIVGPSGSG |
| d-5 | 64-78 | SGSGKSTLAKIVKKI |

g

| Spot | Protein | Residues | Amino acid sequence |
|---|---|---|---|
| C-1 | β-subunit | 29-43 | TMENFIMHKNGKKLF |
| C-2 | β-subunit | 36-50 | HKNGKKLFQIDTYTG |

**Supplementary Figure 14. C-PLoop binds the archaeal 20S proteasome at similar positions to CCRs.** (**a**) Representative peptide array results demonstrating binding of C-PLoop peptides to 20S proteasome isolated from archaea, yeast and human. Control experiments were performed by anti-His and anti-FLAG antibodies without prior incubation of the 20S

proteasomes. (**b**) The bar graphs represent averaged data from four independent experiments as ranked intensities for relative binding of the top 15% C-PLoop peptides to 20S proteasomes from different species. Peptides highlighted in lilac and purple are bound by two and three proteasome species respectively. Error bars ±S.D. (**c**) Table presenting amino acid sequences of the highlighted peptides in (**b**). (**d**) Peptide array experiment for determining the C-PLoop interaction sites on the archaeal 20S proteasome was performed by incubating C-PLoop-His, an anti-His antibody was used as control (lower panel). (**e**) Peptide array design of the α- and β-subunits peptides of the archaeal 20S proteasome and C-PLoop. (**f**) The bar graphs present top 15% archaeal 20S proteasome peptides relative binding to C-PLoop as averages of ranked intensities from four independent experiments. Peptides highlighted in lilac represent consensual binding peptides from CCRs CBR3 and NQO1 (Figure **6c**). Error bars represent SD. (**g**) Table presenting amino acid sequences of the highlighted peptides in lilac (**f**), and their overlapping sequences are underlined. Source data are provided with this paper.

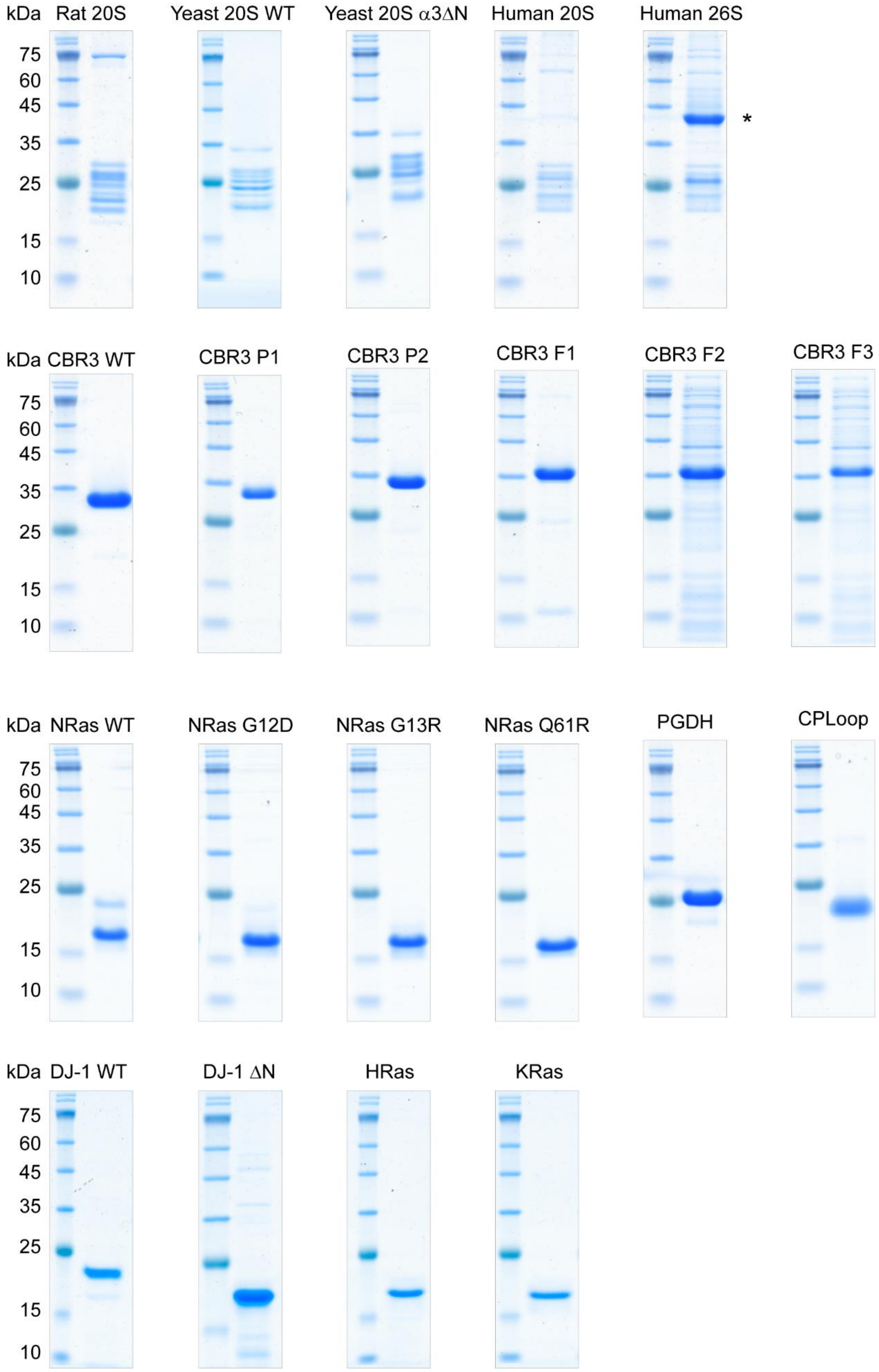
kDa
Rat 20S
Yeast 20S WT
Yeast 20S α3ΔN
Human 20S
Human 26S
*
CBR3 WT
CBR3 P1
CBR3 P2
CBR3 F1
CBR3 F2
CBR3 F3
NRas WT
NRas G12D
NRas G13R
NRas Q61R
PGDH
CPLoop
DJ-1 WT
DJ-1 ΔN
HRas
KRas
75
60
45
35
25
15
10

**Supplementary Figure 15. Purity and integrity of purified 20S proteasomes and CCR proteins.** Purified proteins and protein complexes used in this study were resolved by SDS-PAGE and stained with Coomassie. Asterisk denotes creatine kinase which is used during the purification of the 26S proteasome, together with creatine phosphate, in order to sustain ATP levels that are required for maintaining 26S proteasome integrity.

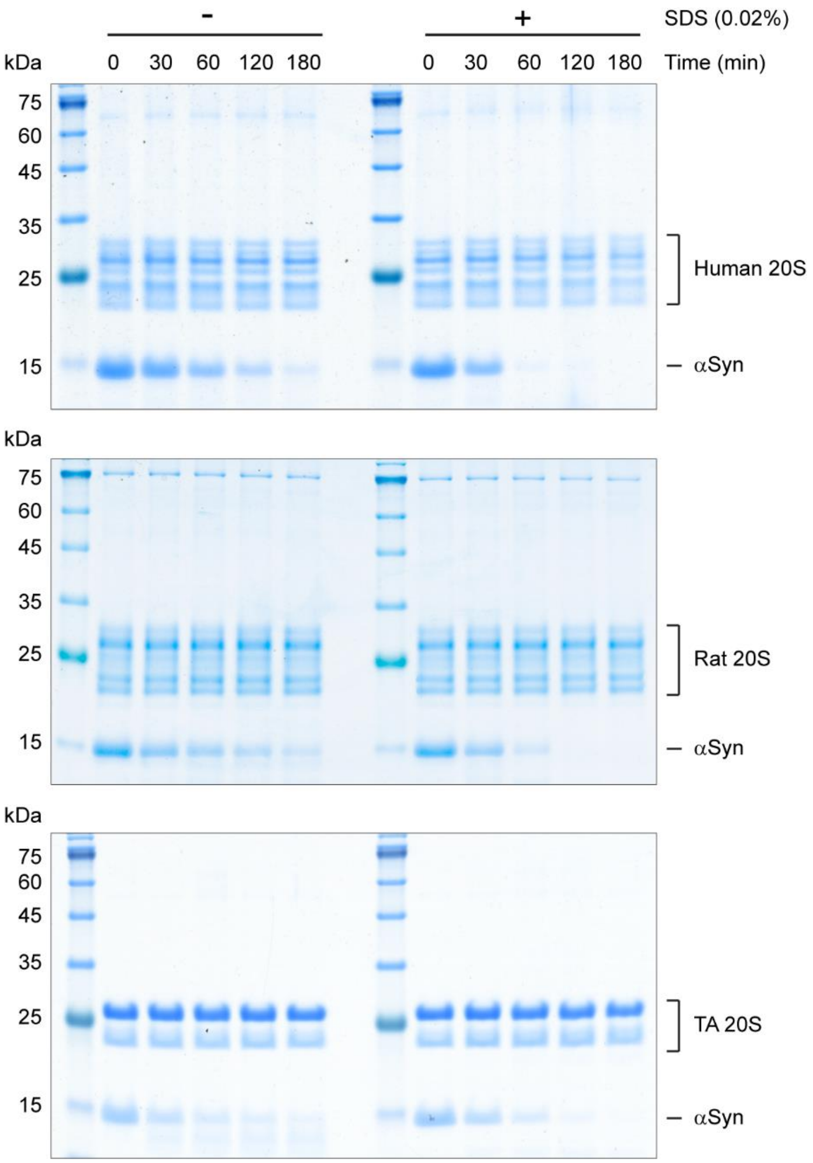


**Supplementary Figure 16. The purified rat, human and bacterial 20S proteasomes are latent.** The rate of α-synuclein degradation was measured in the presence and absence of 0.02% SDS. All three proteasomes exhibited a higher rate of proteolytic activity in the presence of SDS, indicating their latent state. In order to clearly visualize the impact of SDS a three-fold higher concentration of α-synuclein was used in these assays in comparison to the other degradation assays.

**Supplementary Table 1 .** List of P-Loop constructs, their amino acid sequence, folding state and susceptibility towards degradation by the 20S proteasome.

| Name | Sequence | Susceptibility Towards 20S Proteolysis | Folding State |
|---|---|---|---|
| **A-PLoop** | MRLIVVIVGPSGSGKTTLEELAKAAKEAVPEAETREVNTKEEAR KVAEEAEKRNADIVVIIGPSGSGKSTLAEVVKRIIKRAGAKTVDV TTKDEAREAIKRARGSWSLEHHHHHH | ✓ | Dimer |
| **B-PLoop** | MRVIVVIVGPSGSGKTTLEELLKEAKKAAPDAEHREVNTTEEAK KVAEEAKRRNADIVVIIGPNGSGKSTLAEVVRRIIEEAGAEEVRVT TPEEARKAIARARGSWSLEHHHHHH | ✓ | Dimer |
| **C-PLoop** | MRVIVVIVGPSGAGKTTLDELARKAKEEVPDAEIRTVTTKEDAK RVAEEAERRNADIVVIVGPSGSGKSTLAKIVKKIIARAGAKTIEVT TEEELRKAVAKARGSWSLEHHHHHH | ✗ | Dimer |
| **D-PLoop** | MRVIIVIVGPSGAGKTTLLELAKEAKKEVPDAEVRTVTTRDDAK RVAEEAKRRGVDIVVIVGPSGSGKSTLATEIRRIIKEAGARDVEVT TSEELRKAAREARGSWSLEHHHHHH | ✓ | Monomer |
| **E-PLoop** | MNLVVVVVGPSGSGKSTLKENVEKNFPEVEVRLVEDEEDAQEVID QIRNNGVEKVVLVGPSGSGKTTLLEQIKEKAGVQVYRVEDENEL NEVVENQNNGSWGLEHHHHHH | ✗ | Monomer |
| **F-PLoop** | MEKVLMVIGPSGAGKSTLIEEVQKRAENKYPGREIRTATDEEDIQ NIIQEMKNNGKPLVVFVGPSGAGKTTLINEMEQEAQDQGVSYDV LKSNDQEELTQRVRQFLQEDGSWGLEHHHHHH | ✓ | Monomer |
| **2N3Z** | MGRLVVVVVTSEQLKEEVRKKFPQVEVRLVTTEEDAKQVIKEIQK KGVQKVVLVGVSEKLLQKIKQEANVQVYRVTSNDELEQVVKDV KGSGLEHHHHHH | ✗ | Monomer |

**Supplementary Table 2.** Cryo-EM data collection details of the 20S proteasome and CBR3 complex.

| | 20S proteasome & CBR3 complex (EMDB-17118) (PDB-N/A) |
|---|---|
| **Data collection and processing** | |
| Magnification | 47,000x |
| Voltage (kV) | 300 |
| Electron exposure (e–/Å$^2$) | 20 |
| Defocus range (μm) | -1.0-(-2.0) |
| Pixel size (Å) | 1.7 |
| Symmetry imposed | C1 |
| Initial particle images (no.) | 55,555 |
| Final particle images (no.) | 53,138 |
| Map resolution (Å) | ~12 |
| FSC threshold | 0.143 |
| Map resolution range (Å) | 12 - 14 |
| | |
| **Refinement** | |
| Initial model used (PDB code) | 4R3O |
| Model resolution (Å) | 12 |
| FSC threshold | 0.143 |
| Model resolution range (Å) | 12-14 |

**Supplementary Table 3.** Theoretical masses, measured masses and mass errors for all proteins measured by MS.

| Figure | Protein | Theoretical Mass (da) | Measured Mass (Da) | Mass Error (Da) |
|---|---|---|---|---|
| Figure 5b-d | TA 20S ($\alpha_7\beta_7$) | 676,942 | 676,488 | 886.0 |
| | Half TA 20S ($\alpha_7\beta_7$) + propeptides) | 346,819 | 350,120 | 40.0 |
| | TA $\alpha$-ring ($\alpha_7$) | 182,703 | 183,259 | 70.0 |
| | CBR3 | 30,937 | 30,945 | 14.0 |
| | TA $\alpha$-subunit | 26,116 | 26,118 | 15.0 |
| | TA $\beta$-subunit w/o propeptide | 22,272 | 22,275 | 12.0 |
| Figure 5f | Rat 20S | 716,072 | 716,915 | 146.0 |
| Figure 5g | Rat 20S | 716,072 | 716,796 | 324.0 |
| | Rat 20S + CBR3 | 747,009 | 747,912 | 437.2 |
| Figure 5h | Rat 20S | 716,072 | 717,432 | 198.4 |
| | Rat 20S + HRas + GDP | 736,277 | 737,825 | 391.9 |
| Figure 5i | Rat 20S | 716,072 | 717,697 | 161.5 |
| | Rat 20S + NRas + GDP | 736,116 | 737,273 | 930.9 |
| Figure 5j | Rat 20S | 716,072 | 717,787 | 197.1 |
| | Rat 20S + KRas + GDP | 735,815 | 737,822 | 185.2 |
| Figure 9b | TA $\alpha$-subunit | 26,116 | 26,128 | 28.0 |
| | C-Ploop | 12,474 | 12,498 | 19.0 |
| Figure 9c | Rat $\alpha$-subunit | 26,116 | 27,628 | 113.0 |
| | C-Ploop | 12,474 | 12,476 | 37.0 |
| Supplementary Figure 1a | DJ-1 WT | 39,502 | 39,521 | 1.0 |
| | DJ-1 $\Delta$N | 18,966 | 18,968 | 4.0 |
| Supplementary Figure 3b | CBR3 WT | 33,403 | 33,432 | 11.0 |
| | CBR3 WT + NADPH | 34,147 | 34,432 | 13.0 |
| | CBR3 P1 | 33,512 | 33,487 | 32.0 |
| | CBR3 P1 + NADPH | 34,257 | 34,240 | 38.0 |
| | CBR3 P2 | 33,590 | 33,648 | 39.0 |
| | CBR3 P2 + NADPH | 34,335 | 34,440 | 33.0 |
| Supplementary Figure 3c | NRas WT + GDP | 20,044 | 20,046 | 0.2 |
| | NRas G12D + GDP | 20,103 | 20,104 | 0.7 |
| | NRas G12D + GTP + Mg | 20,209 | 20,206 | 0.4 |
| | NRas G13R + GTP | 20,227 | 20,222 | 0.1 |
| | NRas G13R + GDP | 20,144 | 20,142 | 0.1 |
| | NRas Q61R + GTP + Mg | 20,180 | 20,175 | 0.4 |
| Supplementary Figure 3e | CBR3 WT | 33,403 | 33,432 | 11.0 |
| | CBR3 WT + NADPH | 34,147 | 34,432 | 13.0 |
| | CBR3 F1 | 33,319 | 33,377 | 23.0 |
| | CBR3 F2 | 33,394 | 33,450 | 24.0 |
| | CBR3 F3 | 33,442 | 33,496 | 11.0 |
| Supplementary Figure 6a | TA20S | 676,942 | 676,488 | 886.0 |
| Supplementary Figure 6b | CBR3 | 30,937 | 30,945 | 14.0 |
| Supplementary Figure 6c | HRas + GDP | 20,205 | 20,207 | 1.0 |
| Supplementary Figure 6d | NRas + GDP | 20,044 | 20,046 | 1.0 |
| Supplementary Figure 6e | KRas + GDP | 19,743 | 19,745 | 1.0 |
| Supplementary Figure 10b | Endogenous PSMB4 | 24,392 | 24,390 | 1.0 |
| | PSMB4-HA des | 25,849 | 25,846 | 2.0 |
| Supplementary Figure 10c | Endogenous PSMB4 | 24,392 | 24,390 | 1.0 |
| | PSMB4-HA WT | 25,791 | 25,790 | 1.0 |
| Supplementary Figure 10d | PSMB4-HA des | 25,849 | 25,846 | 2.0 |
| | PSMB4-HA WT | 25,791 | 25,790 | 1.0 |
| Supplementary Figure 10e | Endogenous PSMB4 | 24,392 | 24,390 | 1.0 |
| | Endogenous PSMB4 | 24,392 | 24,390 | 1.0 |
