# Supplementary material for: Allosteric regulation of the 20S proteasome by the Catalytic Core Regulators (CCRs) family
Source: Nat Commun. 2023 May 30;14:3126. doi: 10.1038/s41467-023-38404-w (PMC10229617; doi:10.1038/s41467-023-38404-w)
Supplement: Supplementary file 2 — Description of Additional Supplementary Files [file 41467_2023_38404_MOESM2_ESM.pdf]

Supplementary Data 1

List of peptides derived from five CCR proteins, human DJ-1, yeast DJ-1 (Hsp32), archaeal DJ-1, human CBR3 and human NQO1, showing amino acid sequence and residue number corresponding to each spot for the respective proteins on the peptide array. For each peptide mean ranked intensities are indicated, with standard deviation, calculated from three independent experiments. The top 15% of reactive peptides are highlighted in bold. Control peptides are colored with an orange background.

Supplementary Data 2

List of peptides derived from archaeal 20S proteasome α- and β-subunits, showing amino acid sequence and residue number corresponding to each spot for the respective proteins on the peptide array. Alpha and Beta denotes α- and β- subunits of the archaeal T. acidophilum 20S proteasome. For each peptide mean ranked intensities are indicated, with standard deviation, calculated from four independent experiments. The top up to 17% of reactive peptides are highlighted in bold. Control peptides are colored with an orange background.

Supplementary Data 3

Chimera session called "20S-CBR3map.py" presents the 20-CBR3 map "20S-CBR3-complex.mrc" with angular distributions "20S-CBR3-agular-distribution.mrc". The 20S-CBR3 map displaying CBR3 extra density, was obtained from 3D classification presented in Supplementary Figure.7b. CBR3 extra densities were observed at lower contour level and a volume hide dust was applied for the clarity and displayed in Figure. 7A. See methods section for more details.
