# Supplementary material for: Allosteric regulation of the 20S proteasome by the Catalytic Core Regulators (CCRs) family
Source: Nat Commun. 2023 May 30;14:3126. doi: 10.1038/s41467-023-38404-w (PMC10229617; doi:10.1038/s41467-023-38404-w)
Supplement: Supplementary file 6 — Reporting Summary [file 41467_2023_38404_MOESM6_ESM.pdf]

nature portfolio

Corresponding author(s): Michal Sharon

Last updated by author(s): Apr 16, 2023

Reporting Summary

Nature Portfolio wishes to improve the reproducibility of the work that we publish. This form provides structure for consistency and transparency in reporting. For further information on Nature Portfolio policies, see our Editorial Policies and the Editorial Policy Checklist.

Statistics

For all statistical analyses, confirm that the following items are present in the figure legend, table legend, main text, or Methods section.

n/a	Confirmed	
☐	☒	The exact sample size (*n*) for each experimental group/condition, given as a discrete number and unit of measurement
☐	☒	A statement on whether measurements were taken from distinct samples or whether the same sample was measured repeatedly
☐	☒	The statistical test(s) used AND whether they are one- or two-sided<br>*Only common tests should be described solely by name; describe more complex techniques in the Methods section.*
☒	☐	A description of all covariates tested
☒	☐	A description of any assumptions or corrections, such as tests of normality and adjustment for multiple comparisons
☐	☒	A full description of the statistical parameters including central tendency (e.g. means) or other basic estimates (e.g. regression coefficient) AND variation (e.g. standard deviation) or associated estimates of uncertainty (e.g. confidence intervals)
☐	☒	For null hypothesis testing, the test statistic (e.g. *F*, *t*, *r*) with confidence intervals, effect sizes, degrees of freedom and *P* value noted<br>*Give P values as exact values whenever suitable.*
☒	☐	For Bayesian analysis, information on the choice of priors and Markov chain Monte Carlo settings
☒	☐	For hierarchical and complex designs, identification of the appropriate level for tests and full reporting of outcomes
☒	☐	Estimates of effect sizes (e.g. Cohen's *d*, Pearson's *r*), indicating how they were calculated

Our web collection on statistics for biologists contains articles on many of the points above.

Software and code

Policy information about availability of computer code

Data collection	Infinite 200, Tecan Group<br>ThermoScientific MyECL Imager v. 2.2.0.1250<br>Q-Exactive Plus Orbitrap EMR (Thermo Fisher Scientific)<br>UHMR Orbitrap instrument (Thermo Fisher Scientific)<br>nanoDSF Prometheus NT.48 (NanoTemper Technologies GMBH)<br>Q-Star Elite instrument (MDS Sciex)<br>Synapt G1 HDMS (Waters MS Technologies)<br>Titan Krios G3i (Thermo Fisher Scientific)<br>Falcon 3EC direct electron detector (Thermo Fisher Scientific)<br>EPU software v 2.5 (Thermo Fisher Scientific)
Data analysis	ImageJ 1.51k, R v.4<br>MassLynx software (Waters V4.2 SCN982)<br>Xcalibur v. 4.2 (ThermoScientific)<br>Unidec v. 4.1.145<br>GraphPad Prism V6<br>Relion 3.0<br>MotionCor2

CTFFIND4
PR.ThermControl v2.1.1 (NanoTemper Technologies GMBH)
UCSF Chimera 1.16
Microsoft Office Excel 2016

For manuscripts utilizing custom algorithms or software that are central to the research but not yet described in published literature, software must be made available to editors and reviewers. We strongly encourage code deposition in a community repository (e.g. GitHub). See the Nature Portfolio guidelines for submitting code & software for further information.

Data

Policy information about availability of data

All manuscripts must include a data availability statement. This statement should provide the following information, where applicable:

- Accession codes, unique identifiers, or web links for publicly available datasets
- A description of any restrictions on data availability
- For clinical datasets or third party data, please ensure that the statement adheres to our policy

All analyzed data are uploaded with this manuscript as supplementary data files and source data (uncropped images, blots and datapoints underlying in graphs). The datasets generated during the current study are available from the corresponding author on reasonable request. Cryo-EM map determined from the 20S proteasome and CBR3 complex dataset has been deposited at the Electron Microscopy Data Bank with accession codes EMD-17118 [DOI link yet to be released]. There is no PDB entry corresponding to EMD-17118. The human constitutive 20S proteasome structure 4R3O [http://doi.org/10.2210/pdb4R3O/pdb] was used as a reference for initial refinement and rat 20S proteasome structure 6TU3 [http://doi.org/10.2210/pdb6TU3/pdb] was used to superimpose the electron density map of the 20S-CBR3 complex. All constructs (wild type and mutants) used in this study can be obtained on request to corresponding author. Source data are provided with this paper.

Human research participants

Policy information about studies involving human research participants and Sex and Gender in Research.

Reporting on sex and gender	N/A
Population characteristics	N/A
Recruitment	N/A
Ethics oversight	N/A

Note that full information on the approval of the study protocol must also be provided in the manuscript.

Field-specific reporting

Please select the one below that is the best fit for your research. If you are not sure, read the appropriate sections before making your selection.

[x] Life sciences [ ] Behavioural & social sciences [ ] Ecological, evolutionary & environmental sciences

For a reference copy of the document with all sections, see nature.com/documents/nr-reporting-summary-flat.pdf

Life sciences study design

All studies must disclose on these points even when the disclosure is negative.

Sample size	In all experiments, except for one (Figure 8k), we used either 3 or 4 repeats, which are the minimum number of repeats for statistical calculations, and as commonly done in experiments of this type, in our field. We increased the sample size (Figure 8K) since the interactions between 20S and CBR3 are transient and the method is not very sensitive.
Data exclusions	No data was excluded
Replication	All the experiments performed in this study are reliably reproducible. All the in vitro activity assays were performed three times. Further information on the number of replicates is indicated in the relevant figure legends and methods section.
Randomization	No randomization techniques were performed in due to the nature of our study. Treatments/experimental conditions were applied to categorize all the samples and their identity was known before experimental setup.
Blinding	All our analyses are objective measurements in which the outcome is not affected by the fact that the experiment was not performed in a blinded manner.

Reporting for specific materials, systems and methods

We require information from authors about some types of materials, experimental systems and methods used in many studies. Here, indicate whether each material, system or method listed is relevant to your study. If you are not sure if a list item applies to your research, read the appropriate section before selecting a response.

Materials & experimental systems

n/a	Involved in the study
☐	☒ Antibodies
☐	☒ Eukaryotic cell lines
☒	☐ Palaeontology and archaeology
☐	☒ Animals and other organisms
☒	☐ Clinical data
☒	☐ Dual use research of concern

Methods

n/a	Involved in the study
☒	☐ ChIP-seq
☒	☐ Flow cytometry
☒	☐ MRI-based neuroimaging

Antibodies

Antibodies used

anti-HA(R) rabbit (1:6000, ab9110,Abcam)
anti-HA(M) mouse (1:1000, ab18181, Abcam)
anti-PSMD1 (1:1000, ab2941,Abcam)
anti-PSMD2 (1:1000, PAB6715, Abnova)
anti-PSMA3 (1:500, sc-58417, SantaCruz)
anti-PSMA1 (1:1000, ab140499, Abcam)
anti-FLAG (1:2500, F3165, Sigma, Clone M2)
anti-FLAG (1:5000, ab1162, Abcam)
anti-GFP (1:2500, ab290, Abcam)
anti-p53 HRP (1:2500, HAF1355, Biotest)
anti-α-synuclein(1:500, ab51252, Abcam)
anti-GAPDH (1:1000, MAB374, Millipore, Clone 6C5)
anti-CBR3 (1:1000,sc-374393, Santa Cruz)
anti-CBR3 (1:2500, 15619-1-AP, Proteintech) - used in peptide array experiments
anti-NQO1 (1:2500, Ab34173, Abcam)
anti His-HRP (1:1500, sc-8036, Santa Cruz Biotechnology),
anti-His antibody (1:2500, A00174 Genscript),
anti-Rpn2 (PSMD1, ab140682, Abcam) - used for Immunoprecipitation experiments

Validation

Validation reports for all commercial antibodies are provided in the database of manufacturer's website. A representative western blot example is provided in data sheets for all antibodies. Further validation was performed using appropriate controls by Western blot in the lab.

Eukaryotic cell lines

Policy information about cell lines and Sex and Gender in Research

Cell line source(s)

HEK293T cells were purchased from ATCC and maintained in the lab. The T-47D-PSMD2 knock-down stable cell line was obtained from Peter Tsetkov, Broad Institute, USA and maintained in the lab. Yeast strains wild-type (SUB545) and open-gate mutant (SUB544) were obtained from Prof. Michael Glickman, Israel Institute of Technology, Israel

Authentication

We did not authenticat cells by ourselves.

Mycoplasma contamination

All cell lines tested for Mycoplasma were negative.

Commonly misidentified lines (See ICLAC register)

None of the cell lines used in this study were identified in Commonly misidentified line database.

Animals and other research organisms

Policy information about studies involving animals; ARRIVE guidelines recommended for reporting animal research, and Sex and Gender in Research

Laboratory animals

Rattus norvegicus - Norway rat

Wild animals

This study did not involve wild animals.

Reporting on sex	The sex of the animals is insignificant to our study.
Field-collected samples	This study did not involve samples collected from the field.
Ethics oversight	Ethical approval to work with rat livers is not required since livers for proteasome purifications were collected only from redundant rats, that were terminated since they were no longer required for scientific experiments.

Note that full information on the approval of the study protocol must also be provided in the manuscript.
